# *NRAS* mRNA-Degrading Bifunctional Small Molecules Induce Diverse Cellular Morphological Changes in Cancer Cells

**DOI:** 10.1021/jacsau.5c01600

**Published:** 2026-05-11

**Authors:** Mao Jiang, Daniel Hösle, Yang Liu, Sonja Sievers, Peng Wu

**Affiliations:** † Chemical Genomics Centre, Max Planck Institute of Molecular Physiology, Dortmund, 44227, Germany; ‡ Department of Chemical Biology, Max Planck Institute of Molecular Physiology, Dortmund, 44227, Germany; § Faculty of Chemistry and Chemical Biology, TU Dortmund University, Dortmund, 44227, Germany; ∥ Compound Management and Screening Center, Dortmund, 44227, Germany

**Keywords:** RNA degradation, ribonuclease-targeting chimera, NRAS mRNA, anticancer, cellular morphological profile

## Abstract

Therapeutic targeting of the oncogenic NRAS protein, which is constitutively activated in human cancers, with small molecules is a promising yet challenging anticancer strategy. Therefore, targeting the *NRAS* mRNA poses a feasible alternative that will likely yield new biological consequences due to the new mechanism of regulation at the post-transcriptional level. We report herein the first examples of a reversely regulating *NRAS* mRNA-degrading ribonuclease targeting chimeric small molecules (*NRAS*-RIBOTAC), which were assembled by conjugating a reported 4-aminoquinazoline G4-*NRAS* binder with a biphenyl RNase L binder. Among the obtained *NRAS*-RIBOTACs, **5** impacted G4-containing *NRAS* mRNA expression while it did not significantly impact the expression of NRAS protein in MD-MB-231 cells, which could be explained by the fact that the G4-containing *NRAS* transcript only accounts for a trace amount in comparison with the dominant G4-lacking *NRAS* mRNA. Furthermore, we report here for the first time the phenotypic evaluation of RIBOTACs in the unbiased phenotypic profiling method, cell painting assay. In comparison with the monovalent ribonuclease recruiter and G4-*NRAS* mRNA binder, selected RIBOTACs showed significant activities in inducing cellular morphological changes and demonstrated a new biological performance that is reflected by the diverse cellular morphological changes in cancer cells.

## Graphical abstract

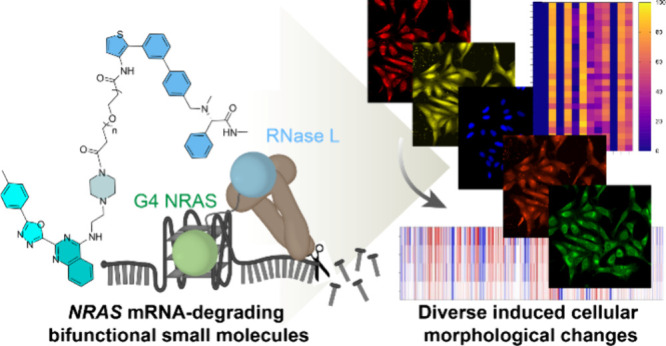

## Introduction

The oncogenic neuroblastoma RAS (NRAS) protein, together with the closely related RAS proteins HRAS and KRAS, is frequently deregulated and constitutively activated in different types of human cancers,^1,2^ including acute myeloid leukemia, melanoma, non-smalll cell lung cancer, and metastatic colorectal cancer. In normal physiological conditions, NRAS proteins switch between the inactive GDP-bound form and active GTP-bound form. In contrast, the hyperactivation of NRAS due to persistent GTP-bound form in cancerous conditions leads to constitutive MAPK and AKT signaling, driving the progression and invasion of cancer cells.^3^ NRAS has therefore been established as a promising target for anticancer therapeutics.

Despite the sustained efforts in developing anti-NRAS therapeutics, it remains a challenging cancer target to be effectively addressed.^4^ The NRAS protein lacks a druggable site apart from the GTP-binding site, and the highly potent binding affinity between GTP and NRAS makes it a daunting task to develop competitive small-molecule inhibitors. Nonetheless, it is noteworthy to mention the encouraging progress achieved in targeting another RAS-family protein KRAS, for which there are currently two FDA-approved small-molecule inhibitors, sotorasib and adagrasib, for non-small cell lung cancer and metastatic colorectal cancer harboring the G12C mutation (Figure 1A).^5–7^

**Figure 1:**
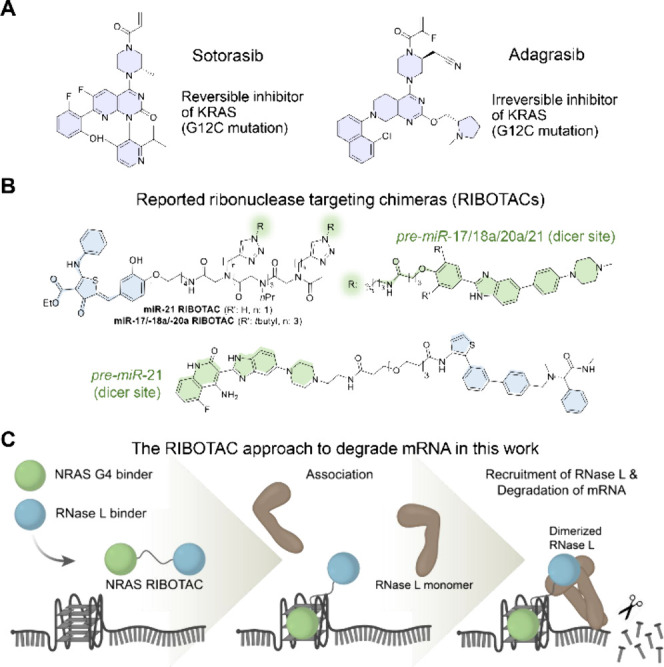
RAS-targeting small molecules and RNA-degrading RIBOTACs. (A) FDA-approved small-molecule inhibitors of KRAS, sotorasib, and adagrasib. (B) Selected examples of oncogenic miRNA-degrading ribonuclease-targeting chimeras (RIBOTACs) by recruiting the latent ribonuclease (RNase L). The RNase L recruiting small molecule is shown with light blue rings; the miRNA-binding small molecule in shown with light green rings. (C) In this study, we demonstrated the first examples of monovalent *NRAS* binder-based RIBOTAC that degrades G4-*NRAS* mRNA, followed by the first demonstration of profiling cellular morphological changes via the cell painting assay.

In this context and as a part of our continued efforts in targeting protein-RNA interactions, a feasible alternative strategy would be to target the *NRAS* mRNA to modulate *NRAS* expression at the transcriptional level. For example, a novel class of small-molecule binders to G4-*NRAS* with submicromolar affinity was recently reported, in which the representative compound JS18 inhibited the translation of *NRAS* mRNA in vitro.^8^ Additionally, other G4-*NRAS* targeting ligands have been recently reported.^9,10^

Apart from monovalent small molecules binding to RNAs,^11–13^ the bifunctional molecule strategy of generating ribonuclease-targeting chimera (RIBOTAC) is emerging as a promising alternative to target RNAs and expand the druggable genome, particularly for disease-associated undruggable or unligandable targets.^14^ The RIBOTAC strategy has been successfully demonstrated to degrade a collection of cancer-associated RNA substrates, such as miR-21, miR17–92 cluster, QXOX1 mRNA, and miR-210 (Figure 1B).^15–20^ Additionally, a proximity-induced nucleic acid degrader strategy was employed to degrade the G-quadruplexes (G4) of the SARS-CoV-2 genome and betacoronaviral pseudoknots.^21^ For mRNA degradation, an inducible RIBOTAC strategy combining a caged latent ribonuclease (RNase L) recruiter and a bivalent G4 binder was demonstrated to knock down G4 RNAs upon activation under biorthogonal and cell-specific stimulus.^22^ Recent work in the related field also includes the degradation of G4 RNA-binding proteins via the proteolysis targeting chimeric strategies.^23,24^ In this work, we demonstrated the utilization of a monovalent G4 binder to assemble the *NRAS* mRNA targeting RIBOTACs to achieve the targeted degradation of *NRAS* mRNA (Figure 1C). Furthermore, we report here for the first time that the cell painting assay,^25,26^ measuring cellular morphological changes based on microscope images, can be a useful phenotypic assay to profile the complex morphological changes induced by RIBOTACs, expanding the downstream analysis and enhancing RIBOTAC discoveries.

## Results and Discussion

### RIBOTAC 5 Degraded G4-*NRAS* mRNA

To initiate the *NRAS*-targeting RIBOTAC synthesis, we envisioned incorporating a biphenyl small-molecule binder MD4 that recruits RNase L with a structurally modified analogue JS18 with appended functional groups as the two main building components to assemble such bifunctional RIBOTAC molecules (Figure 2).

**Figure 2:**
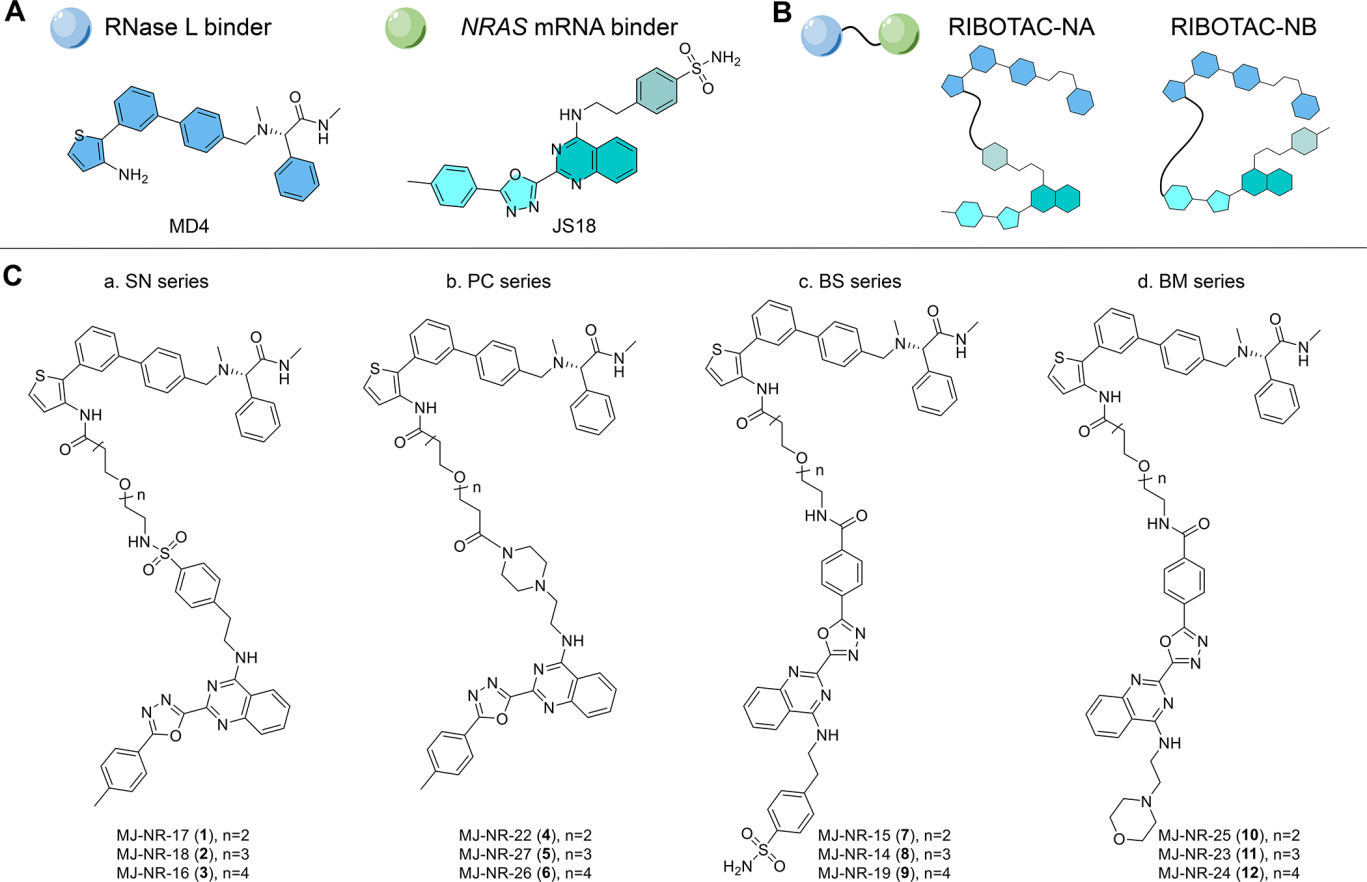
Bifunctional *NRAS* mRNA-targeting RIBOTACs evaluated in this study. (A) Building components for the bifunctional molecules: the reported RNase L recruiting biphenyl phenylacetamide MD4 and the reported *NRAS* mRNA binding aminoquinazolinyl sulfonamide JS18. (B) Different conjugation strategies are used for the construction of the RIBOTACs. (C) RNase L binder linked via the terminal sulfonamide at the 4-amino substituent on the quinazoline core scaffold of the *NRAS* binder (sulfonamide linking series, SN), linked via the terminal piperazinylcarbonyl at the 4-amino substituent of the *NRAS* binder (piperazinylcarbonyl linking series, PC), linked via the terminal benzamide at the 2-oxadiazolyl substituent of the quinazoline core scaffold of the *NRAS* binder with a benzensulfonamide at the other unlinked end (benzamide linking series with sulfonamide series, BS), and linked via the terminal benzamide at the 2-oxadiazolyl substitutent but with a morpholine at the other unlinked end (benzamide linking series with morpholine series, BM).

Given that the exact binding mode for JS18 with the *NRAS* G4 is not yet elucidated, we designed a set of four series of bifunctional *NRAS* RIBOTAC featuring varied appendage positions on the aminoquinazoline score scaffold to probe an optimal linking attachment position that would not lead to diminished G4-*NRAS* binding affinity for the envisioned bifunctional molecules. Specifically, the RNase L binder was linked via the terminal sulfonamide at the 4-amino substituent on the quinazoline core scaffold of the *NRAS* binder JS18 (sulfonamide linking series, SN), linked via the terminal piperazinylcarbonyl at the 4-amino substituent of the *NRAS* binder (piperazinylcarbonyl linking series, PC), linked via the terminal benzamide at the 2-oxadiazolyl substituent of the quinazoline core scaffold of the *NRAS* binder with a benzensulfonamide at the other unlinked end (benzamide linking series with sulfonamide series, BS), and linked via the terminal benzamide at the 2-oxadiazolyl substituent but with a morpholine at the other unlinked end (benzamide linking series with morpholine series, BM). Together with the incorporation of different linker lengths, a collection of 12 RIBOTACs was thus obtained based on the synthetic routes summarized in Scheme 1.

**Scheme 1:**
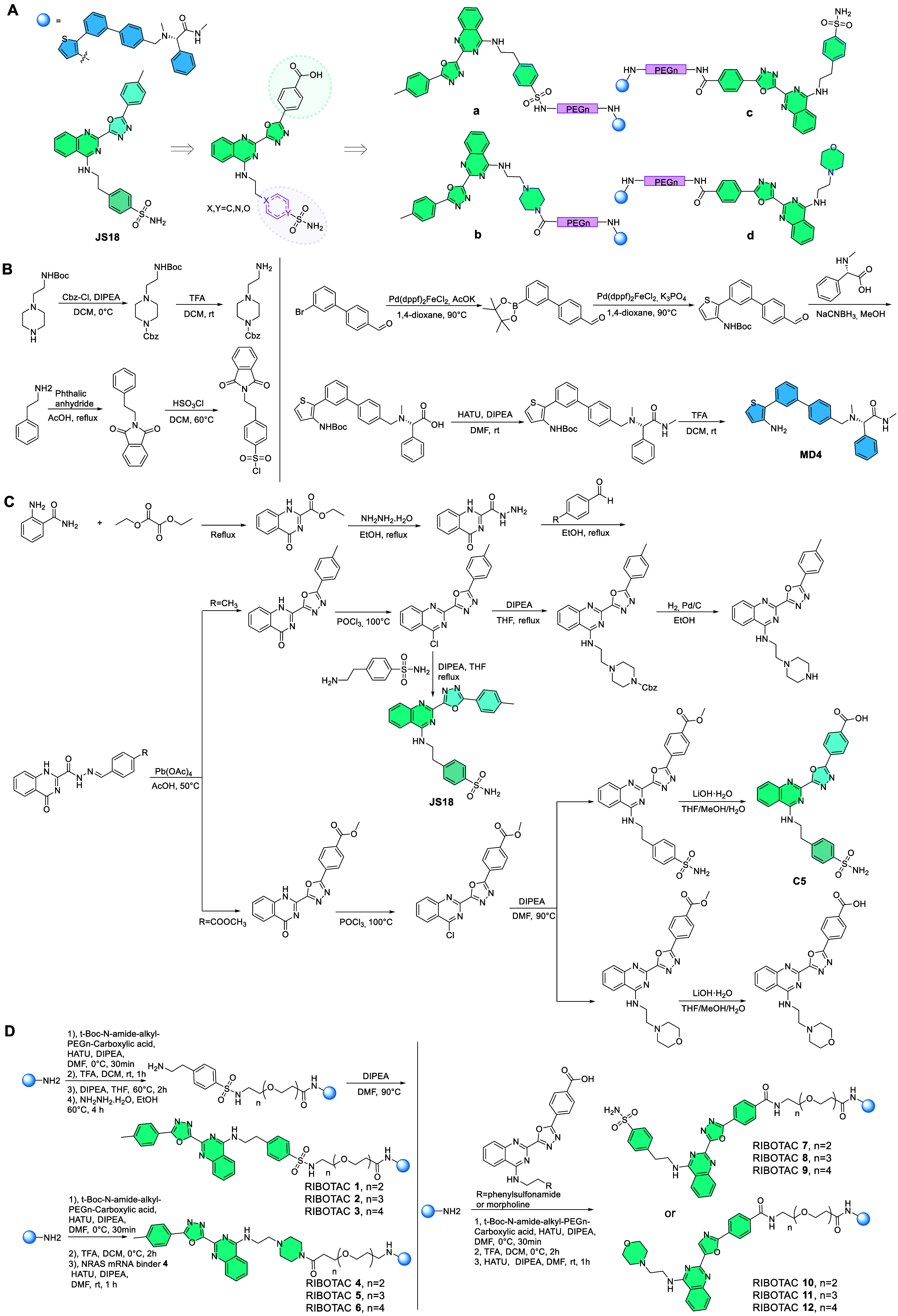
Synthesis of the *NRAS* RIBOTACs **1-12** ^a^

a (A) Overview of the four different linking strategies between the RNase L binder MD4 and the NRAS mRNA binder JS18. (B) Synthesis of the RNase L binder MD4. (C) Synthetic route for the *NRAS* mRNA binders JS18, C5, and analogues. (D) Assembly of the *NRAS* RIBOTACs **1-12**.

We first tested the *NRAS* mRNA expression levels in the MDA-MB-231 cells. As shown in the RT-qPCR results, none of the 12 tested RIBOTACs significantly affected the expression levels of total *NRAS* mRNA (Figure 3A, Table S1). However, the expression of G4-*NRAS* mRNA showed varied results, with RIBOTAC **5** leading to an ∼25% reduction in G4-*NRAS* at 10 μM (Figure 3B, Table S1). Testing of RIBOTAC **5** at concentrations ranging between 10 and 40 μM in MCF-7 cells echoed the marginal degradation activity that was observed in testing in MDA-MB-231 cells (Figure S1, Table S2). To evaluate the selectivity of RIBOTAC **5** among different G4-containing transcripts, we further performed the RT-qPCR testing for RIBOTAC **5** involving selected G4-containing transcripts, including *NRAS, HRAS, KRAS, ADAM10, Bcl2* and *cMyc*. The result showed that RIBOTAC **5** exhibited limited selectivity toward G4-*NRAS* over the other tested G4-transcripts, despite the marginal reduction level of NRAS expression (Figure S2). Given the RT-qPCR results, we proceeded to test the NRAS protein level by Western Blot, expecting to observe the same trend of reduced NRAS expression. However, no significant changes in the NRAS protein level were observed at both 1 and 10 μM (Figure 3C). Intrigued by the surprising result, we checked previous reports on the potential function of the G4 species in modulating mRNA translation and found a possible explanation. Abundance-wise, the G4-containing *NRAS* transcripts were reported to account for less than 1%, while the majority of *NRAS* transcripts lack the presence of the G4 structure at the 5′-UTR.^8^ Therefore, although **5** led to the degradation of the <1% G4-containing transcript, it did not impact the overall NRAS protein expression level. It is noteworthy to mention that function-wise, the G4-containing *NRAS* was reported to function as an inhibiting species for the translation of the predominant non-G4 *NRAS* transcripts.^27^

**Figure 3:**
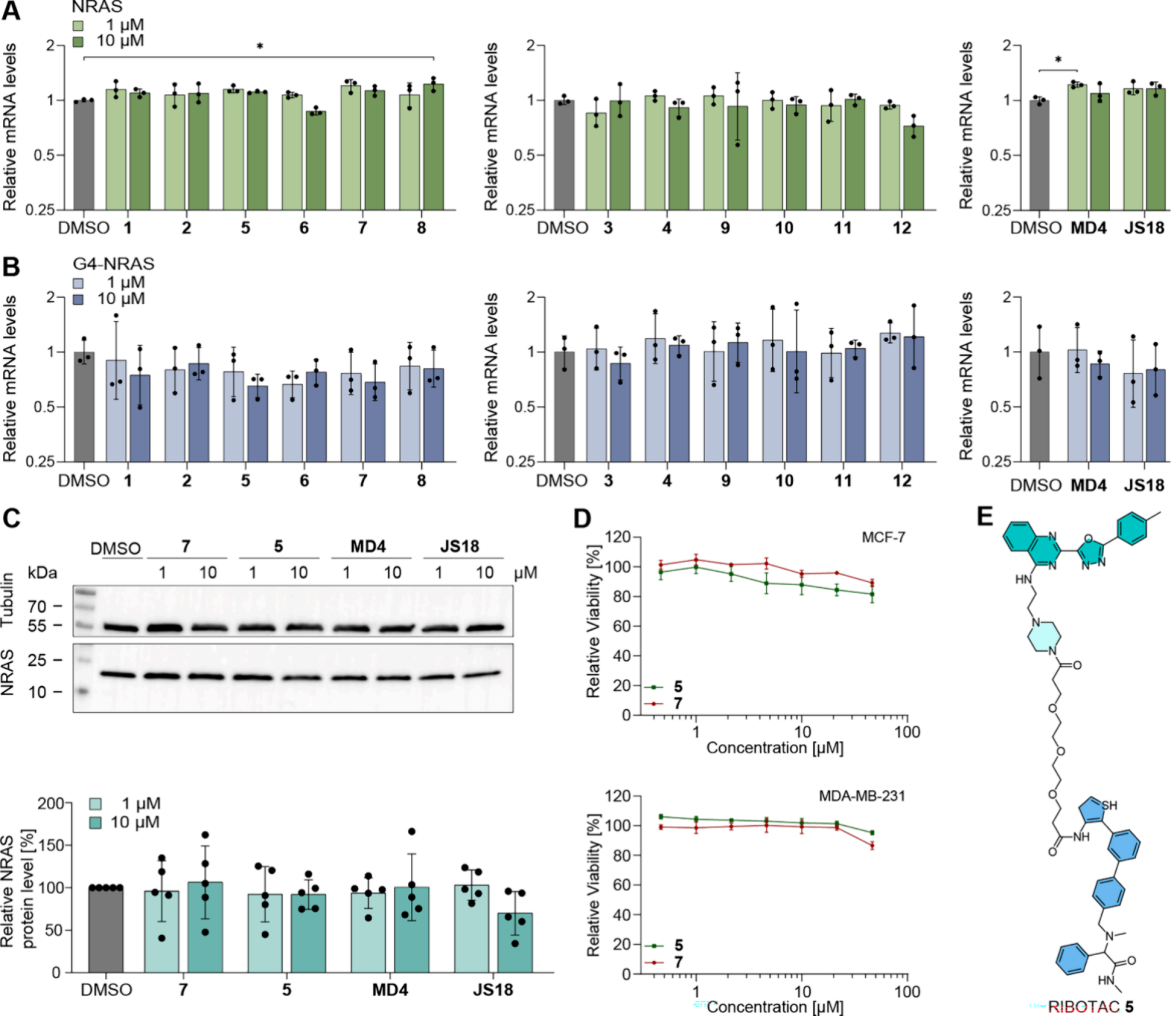
Cellular evaluation of *NRAS*-RIBOTACs. (A) The tested RIBOTACs **1–12** did not impact the expression of the total *NRAS* mRNA levels measured in RT-qPCR. (B) RIBOTAC **5** degraded G4-containing *NRAS* mRNA to 10 μM. (C) RIBOTACs including **5** and **7** did not significantly impact the NRAS protein expression in the Western blot. (D) RIBOTACs **5** and **7** did not show cytotoxicity by measuring the cell viability in the CCK8 assay in MCF-7 and MDA-MB-231 cells. (E) Structure of RIBOTAC **5**. The qPCR data are shown as geometric mean ± geometric SD for the relative mRNA levels in panels A and B (three independent biological replicates; the adjusted p-values are summarized in Table S1). The statistical significance was calculated using a one-way ANOVA followed by Dunnett’s posthoc test on ΔC_t_ values. The Western blot quantification data shown in panel C are presented as mean ± SD (five independent biological replicates). Protein levels were normalized to the loading control and expressed as a percentage of the respective internal DMSO control (set to 100%). Statistical significance was determined by Repeated Measures (RM) One-way ANOVA followed by Dunnett’s posthoc test performed on log-transformed data (*p < 0.05, **p < 0.01). The cell viability data shown in panel D are presented as mean ± SD (three independent biological replicates).

Furthermore, to evaluate the activity of the obtained RIBOTACs in impacting the viability of cancer cells, we tested RIBOTACs **5** and **7** in *NRAS*-expressing cancer cells, MCF-7 and MDA-MB-231, which showed no or weak cytotoxicity up to the highest tested concentration of 46 μM (Figure 3D). The cellular evaluation results showed that RIBOTAC **5** (Figure 3E) did not significantly impact NRAS protein expression or the overall cellular viability.

### *NRAS*-Targeting RIBOTACs Induced Cellular Morphological Changes with Varied Activities

RIBOTACs are a class of emerging new chemical modality for which the mode of action at the cellular level, safety, and toxicity profile, and various other physicochemical and biological properties are understudied. Enlightened by the *NRAS* mRNA degradation and elevated NRAS protein expression results, we were curious if a phenotypic assay measuring cellular morphological changes (i.e., the cell painting assay, CPA) could provide useful information in elucidating the unclarified aspects, especially regarding unrevealed biological targets and mechanisms of action.^28–34^ Previously, the CPA has been demonstrated as a useful assay to unfold “hidden” biological information for bifunctional molecules in the format of PROTACs,^35–37^ while no report of applying CPA analysis on RIBOTAC molecules has been performed yet.

To demonstrate whether such a whole-cell imaging assay can be useful to study the biological profiles of RIBOTACs, the obtained *NRAS*-RIBOTACs of four conjugation series were subjected to the CPA. The assays were performed in the human osteosarcoma U2OS cells in different concentrations, initially ranging between 10 and 50 μM. The resulting cell images obtained after staining eight cellular compartments (including the nucleus, nucleoli, mitochondria, endoplasmic reticulum, Golgi, plasma membrane, actin cytoskeleton, and cytosolic RNA) in six different dyes were used to extract a total of 579 morphological features employing high-content screening and image analysis. This resulted in the generation of morphological fingerprints that were used to compare the biological similarities and differences among the tested compounds. As a quantitative measurement of bioactivity, a quantified induction value based on the generated fingerprints described the fraction of significantly altered parameters in comparison to the vehicle control (in percent). A compound is considered biologically active with an induction value of >5%.^38^

The 12 RIBOTACs showed varied performance in CPA, as summarized in Figure 4. The CPA data for RNase L recruiter MD4 and G4-*NRAS* binder C5 (an analogue of JS18) were performed in parallel for comparison. Overall, among the four series, the RIBOTACs of the PC and BM series (**4**, **5**, **6** and **10**, **11**, and **12**) showed more potent activities than the RIBOTACs of the SN and BS series (**1**, **2**, **3** and **7**, **8**, and **9**). For example, at 10 μM, the RIBOTACs of the PC and BM series showed an induction value of >25% and >36%, respectively; while the RIBOTACs of the SN and BS series were either inactive (<5%) or showed weak induction activity of <9%. The activity disparity between PC/BM and SN/BS series could be associated with the structural differences of incorporating a saturated ring (either a piperazine in the PC series or a morpholine in the BM series), which could lead to more favorable physicochemical properties and permeability of the overall bifunctional molecules. The RIBOTAC **5,** which showed the most potent G4-*NRAS* degrading activity, showed significantly improved activity in comparison with that of the C5 at 10 μM. Additionally, the different linker length among each of the same series was another deciding factor impacting the activity. Take the three RIBOTACs of the BS series as an example; **7** with the shortest linker (PEG unit, n = 2) was inactive in all three tested concentrations, **8** of the intermediate linker length (PEG unit, n = 3) was only weakly active at the highest tested concentration of 50 μM (8.8%), while 9 with the longest linker (PEG unit, n = 4) showed induction of 64.8% at 50 μM. Apart from the varied activities, the CPA results indicated the reduced toxicity of the RIBOTACs in comparison with the parent monovalent RNase L recruiter MD4, which was used to assemble the bifunctional molecules. All tested RIBOTACs showed a cell count value of >84% regardless of the testing concentrations and the different conjugation series. In contrast, MD4 showed a relative cell count of <50% at 50 μM, which was deemed too toxic in the CPA assay.

**Figure 4:**
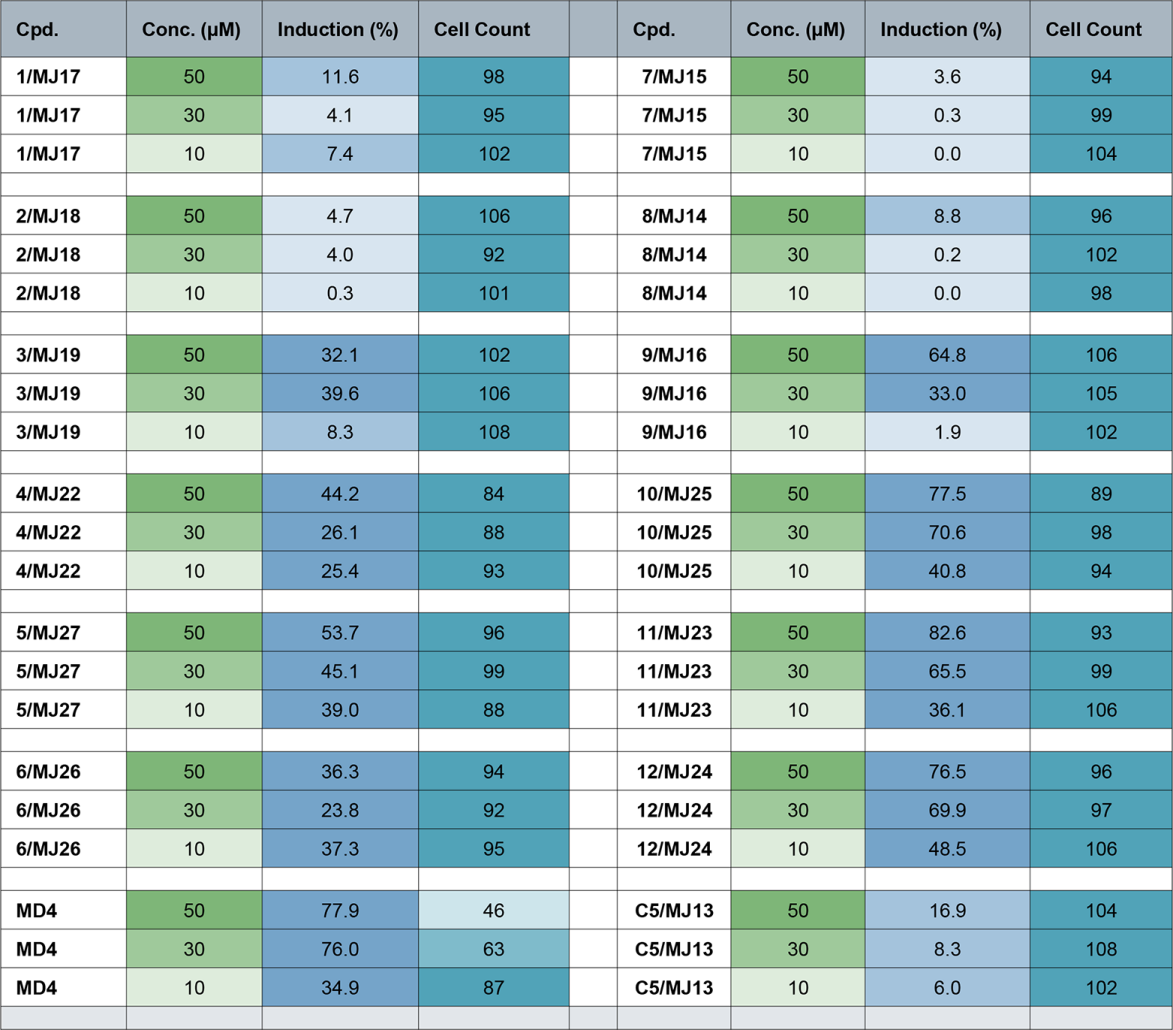
Quantified results of testing the *NRAS*-RIBOTACs in the cell painting assay (CPA). All compounds were tested in three concentrations (50 μM are highlighted in moderate green, #7EB678; 30 μM are highlighted in light green, #79FC99B; 10 μM are highlighted in very light green, #DEECDC). For the Induction: values of more than 20% are highlighted in moderate blue (no. 75A4C9); values of less than 20% but more than 5% are highlighted in light blue (no. A4C3DC); values of less than 5%, highlighted in very light blue (no. D8E5F0), are deemed as biologically inactive in the CPA analysis. For Cell Count: for values of 100 ± 20% are highlighted in moderate cyan/muted aqua (#51A3B8); for values of less than 80% but more than 50% are highlighted in light cyan (#7FBBCB); Values of less than 50%, highlighted in very light cyan (#7CDE5EB), indicate growth arrest. The *NRAS* binder C5 and the RNase L binder MD4 are used in the CPA as the comparison compounds.

The analysis of the biological similarities and differences among the tested compounds was based on the obtained morphological fingerprints and the resulting biosimilarity score, which was calculated based on Person’s correlation distance. Compounds that display similar fingerprints and have a high biosimilarity score are expected to have a high level of biological similarity and share the same biological targets or mode of action. Using the fingerprint of MD4 or C5 as the reference, the resulting biosimilarity score and the fingerprints from the CPA for the RIBOTACs are summarized in Figure 5 and Figure S3, which revealed three general points. First, the RIBOTACs induced significantly different cellular morphological changes from those of the parent monovalent MD4 or C5, with the biosimilarity values <73% (Figure 5A) and <66% (Figure S3), respectively, for all RIBOTACs at the different concentrations. This could be extrapolated to indicate that the RIBOTACs could harbor new modes of action that are not inherited from the monovalent parent molecules. Second, the RIBOTACs induced significantly varied similarity profiles at different concentrations. For example, the CPA fingerprint of **5** shared ∼ 60% similarity with C5 at both 10 and 30 μM, but the similarity was reduced to 28% at the increased concentration to 50 μM. Third, the cytoplasm is more prone to be morphologically changed by the active RIBOTACs, in comparison with the nuclei, as the values were more dramatically changed in the cytoplasm parameters (230–461) than the nuclei parameters (462–579), which is vividly shown by the color contrast shown in Figure 5A between these two regions. Being bulky bifunctional molecules, this can probably be explained that the RIBOTACs likely have a higher concentration in the cytoplasm instead of being penetrable to arrive in the nuclei.

**Figure 5:**
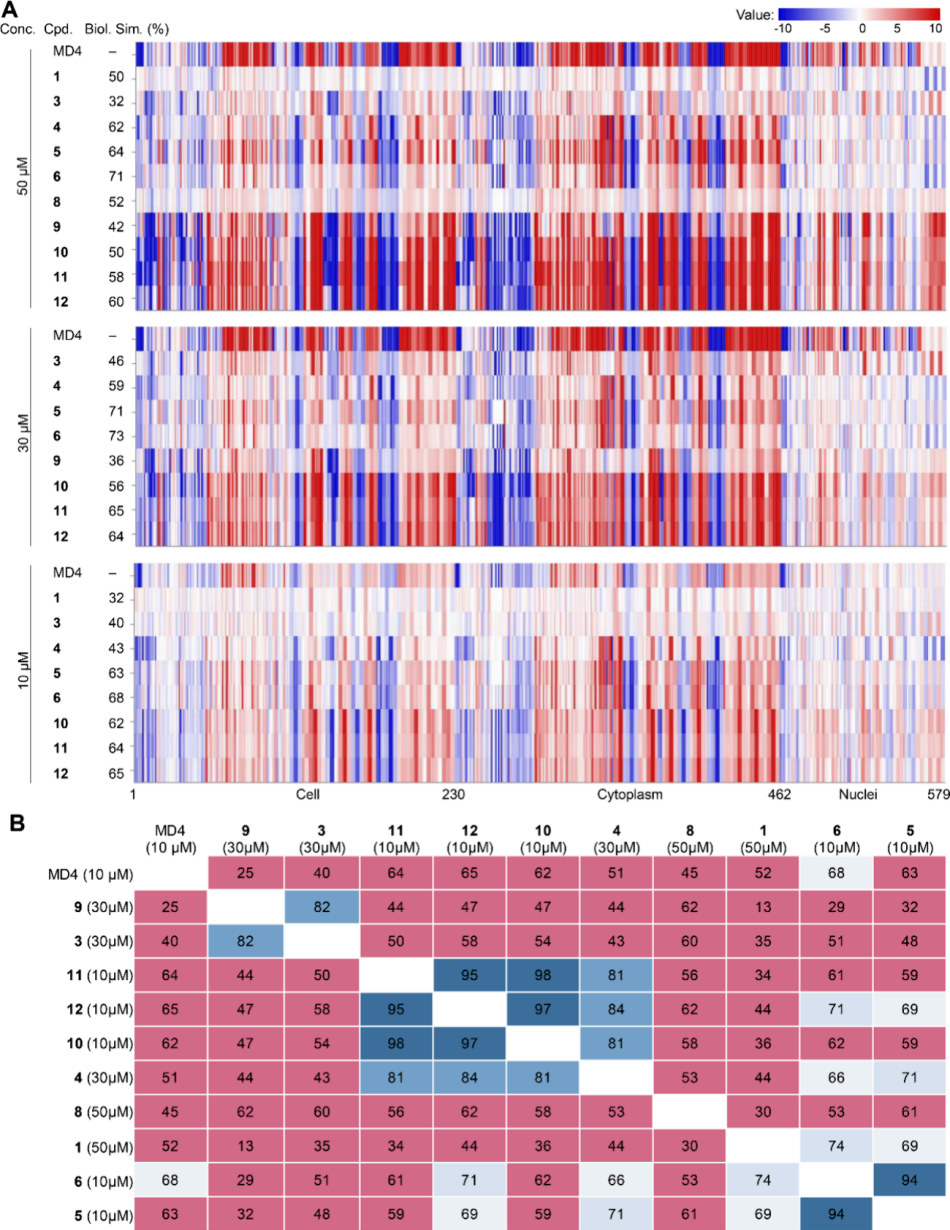
Heatmap showing the cell painting assay results for the RIBOTACs and the cross biosimilarity among the tested compounds. (A) Summarized heatmap for the tested RIBOTACs. The top line of the fingerprint of the heatmap is the result for compound MD4, which is set as a reference fingerprint (100% biological similarity), to which the following RIBOTACs are compared for their biological similarity (Biol. Sim. %). MD4 and each of the RIBOTACs were tested in three concentrations (Conc.): 50 μM, 30 μM, and 10 μM. The fingerprints were generated only for compounds that showed an induction of >5%. The set of 579 parameters is divided into parameters (the *y*-axis) related to the cell (1–229), cytoplasm (230–461), and nuclei (462–579). Decreased parameters are shown in blue, and increased parameters are shown in red. (B) Cross-biosimilarities among the active RIBOTACs of varied concentrations (induction >30%) tested in the CPA analysis, percent values are given. Biosimilarity (%) of more than 90% are highlighted in dark blue (#3D729D); biosimilarities (%) of more than 80% but less than 90% are highlighted in blue (#75A4C9), biosimilarity (%) of more than 70% but less than 80% are highlighted in light blue (#D8E5F0), biosimilarity (%) of more than 65% but less than 70% are highlighted in off-white blue (#EEF3F8), and low biosimilarities of less than 65% are highlighted in light magenta (#D56988).

For the RIBOTACs that showed moderate to potent activities in inducing cellular morphological changes in CPA, we proceeded with cross-biosimilarity analysis. As shown in Figure 5B, the only case of high cross biosimilaritiy within a single conjugation series (>85%) was observed among the RIBOTACs **10–12** of the BM series. In the PC series, RIBOTACs **5** and **6** of varied linker length, differing by only one polyethylene glycol (PEG) unit in the linker composition, showed a high cross biosimilarity (94%), while RIBOTACs 4 and 5, which also differed only by one PEG unit in the linker, did not show a sufficient level of high cross-biosimilarity. The same trend of low cross-biosimilarity was observed for RIBOTACs **1** and **3** of the SN series and **8** and **9** of the BS series, as well as the cross-biosimilarity across the four different series in general, which indicated that they possess different mechanisms of action in inducing the observed cellular morphological changes. Overall, the cross-biosimilarity of the active RIBTOACs demonstrated that even by using the same monovalent building components (RNase L binder MD4 and the NRAS binder JS18), RIBOTACs of diverse biological performance could be obtained by simply diversifying the linker length or applying different conjugation strategies to build the RIBOTACs.

For RIBOTAC **5**, which showed the most promising G4-*NRAS*-degrading activity, we proceeded with testing the CPA at a lowered concentration of 3 μM (Figure 6). Taking the CPA result at 50 μM as the reference fingerprint, it was demonstrated that **5** showed high biosimilarity at concentrations up to 10 μM. At 3 μM, although **5** is active in CPA with an induction of 13%, it is noteworthy that the biosimilarity is reduced to 66% in comparison with that of the 50 μM (Figure 6A), indicating the concentration-dependent variation of biological performance for RIBOTACs. This highlights the importance of selecting an optimal concentration when treating RIBOTACs, echoing the same experience learned from small-molecule modulators and other proximity-inducing modalities, including PROTACs. In the case of **5** at 3 μM, we further scrutinized the line plot (Figure 6B). Among the 579 parameters contributing to the overall CPA profile, the individual parameters that were varied most (value > ±10) include intensity of ER at cytoplasm (value 12.25), radial distribution of Golgi at cytoplasm (value 11.8), inverse difference moment of Golgi at cytoplasm (value −11.20), and Nuclei area shape perimeter (value −10.50) (Table S1). The most varied parameters are the same for RIBOTAC **6** tested at the different concentrations (Figure S4), possibly indicating sharing overlapping targets despite the overall difference in the CPA biological profile (Figure 6C and Figure S5).

**Figure 6:**
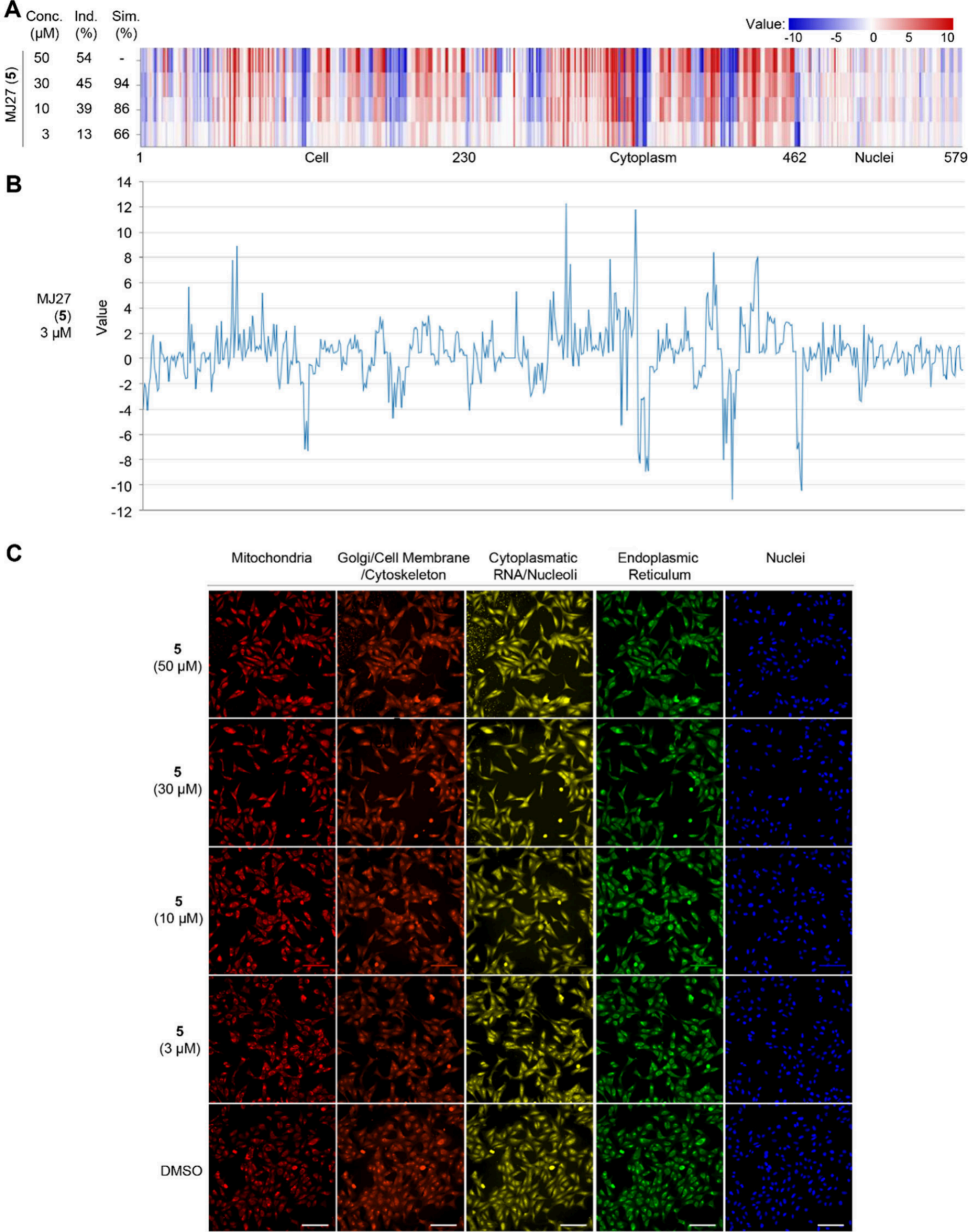
Cell painting results showing the biological similarities of RIBOTAC **5** tested at different concentrations in U2OS cells. (A) The top line of the fingerprint of the heatmap is the result for RIBOTAC **5** tested in 50 μM, which is set as a reference fingerprint (100% biological similarity), to which the following testing results at reduced concentrations (30 μM, 10 μM, and 3 μM) are compared for their biological similarity (Biol. Sim. %). (B) Line plot showing the individual changes of the 579 parameters for RIBOTAC **5** tested in 3 μM. Positive values correspond to the ones shown in red in the fingerprint of the above heatmap (such as parameter No. 299 for cytoplasm intensity of ER, value 12.25; parameter No. 348 for cytoplasm radial distribution of Golgi, value 11.8); negative values correspond to the ones shown in blue in the fingerprint of the above heatmap (such as parameter No. 416 for cytoplasm inverse difference moment of Golgi, value −11.20; parameter No. 465 for Nuclei area shape perimeter, value −10.50). (C) Microscope images from the CPA analysis in U2OS cells upon treating with the RIBOTAC **5** at different concentrations (50, 30, 10, and 3 μM). DMSO was used as the control. Scale bar, 150 μm for all images.

### *NRAS*-RIBOTACs Represent an Unknown Cluster with New Modes of Action

CPA has been successfully applied to identify biological targets and modes of action for unknown compounds by comparing the CPA profiles with reference compounds of annotated bioactivities^28^ or by comparing the CPA profiles with established bioactivity clusters in subprofile analysis.^30^ A high similarity of >85% to a reference compound or an established bioactivity cluster can facilitate the characterization of compounds without prior knowledge on targets and modes of action. We therefore performed the subprofile analysis for the 12 RIBOTACs, each at three tested concentrations. By comparison with the 13 established bioclusters, none of the tested RIBOTACs at any concentration showed a high biosimilarity with any of the established clusters (Figure 7). Take the results for **5** as a representative, it showed no similarity (0%) with the AKT-PI3K-mTOR cluster, Aurora kinase cluster, DNA-synthesis cluster, and pyrimidine-synthesis cluster, and it showed varied low levels of similarities ranging between 9% and 66% with the BET, HDAC, HSP90, LCH, mitrostress, Na_K-ATPase, protein-synthesis, tubulin, and uncoupler clusters (Figure S6). The same summary was concluded by comparing reference compounds that shared the highest similarity with the tested RIBOTACs. Collectively, the subprofile analysis indicated that the RIBOTACs showed low to minimal biosimilarities with the established clusters and probably represent an unknown cluster with new modes of action.

**Figure 7:**
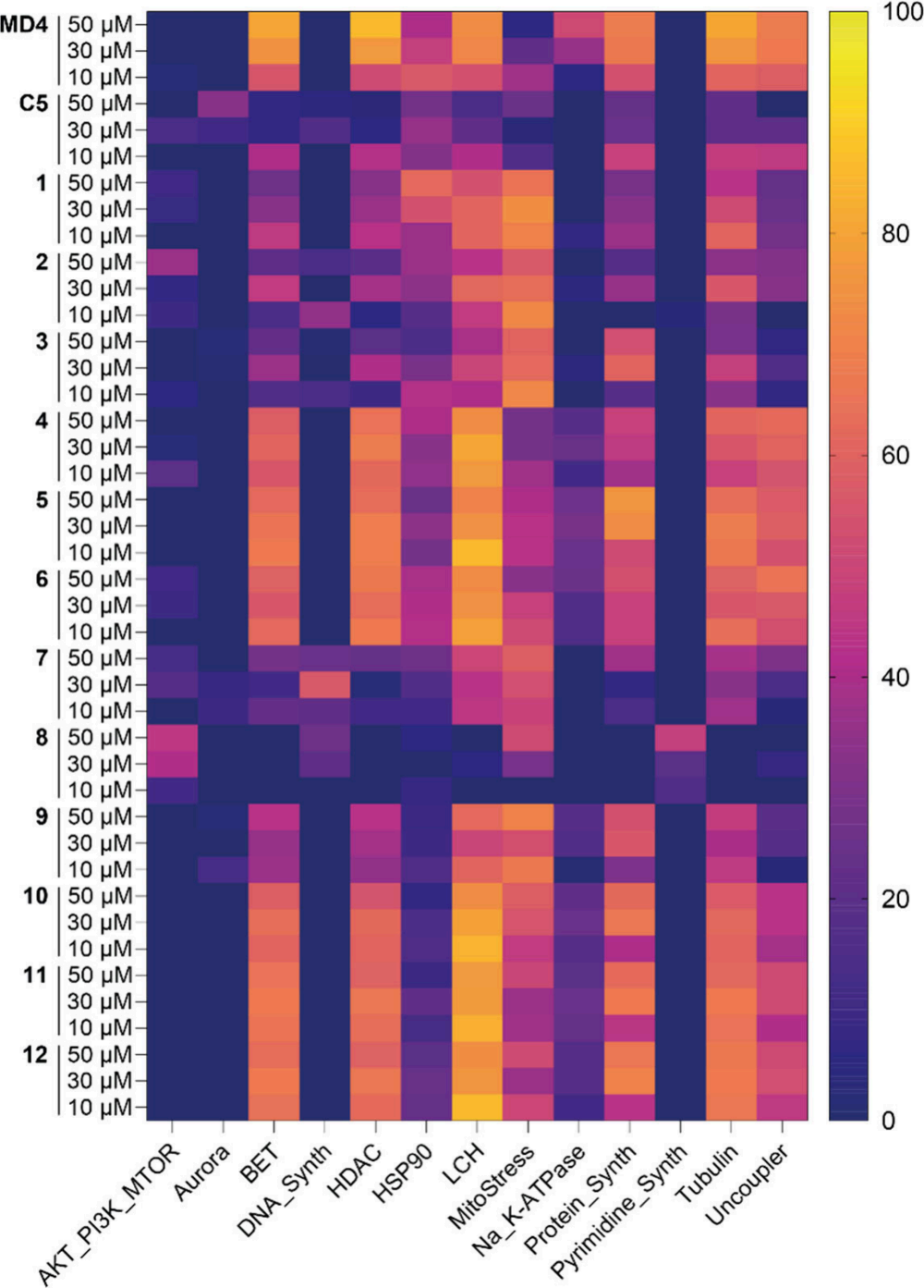
Cluster biosimilarity analysis of the RIBOTACs at different concentrations. The biosimilarity values in percentage are visualized by a gradient series of colors (deep purple blue, 0% – bright yellow, 100%). The 13 established clusters are associated with AKT/PI3K/MTOR, aurora kinase, bromodomain, and extra-terminal domain (BET), DNA synthesis, histone deacetylase (HDAC), heat shock protein 90 (HSP90), lysosomotropism/cholesterol homeostasis (LCH) regulation, mitochondrial stress regulation, Na^+^/K^+^ ATPases, protein synthesis, de novo pyrimidine biosynthesis, tubulin, and uncoupling of the mitochondrial proton gradient.

### Transcriptional Targets of RIBOTAC 5 in RNA-seq

To further probe the potential cellular targets of RIBOTAC **5** at the transcriptional level, we proceeded with RNA sequencing analysis based on the RT-qPCR sample tested in MCF-7 cells (10 μM RIBOTAC **5**). The results showed that 654 genes were present only in the control samples (Figure 8A), and a limited number of genes were significantly up- or downregulated (Figure 8B). For the 654 genes that were present only in the control samples, it could be possible that RIBOTAC **5** treatment led to depletion of the involved genes. In parallel, for the 599 genes that were expressed exclusively in the RIBOTAC **5**-treated cells, an alternative possibility is that RIBOTAC **5** could have a silencing effect on the expression of 654 genes in the control samples. Among the significantly differentiated genes, 5 were upregulated, and 11 were downregulated. The former include the p53 transcription factor family tumor protein p63 (TP63) that mediates tumorigenesis,^39^ the SRC tyrosine kinase family hematopoietic cell kinase (HCK) that has been studied as a cancer target,^40^ the developmental gene chordin (CHRD) that is amplified and expressed in cancers,^41^ the ATP-binding cassette subfamily C member 13 transporter (ABCC13), and gene AP001284.1, whose function is yet to be revealed.

**Figure 8:**
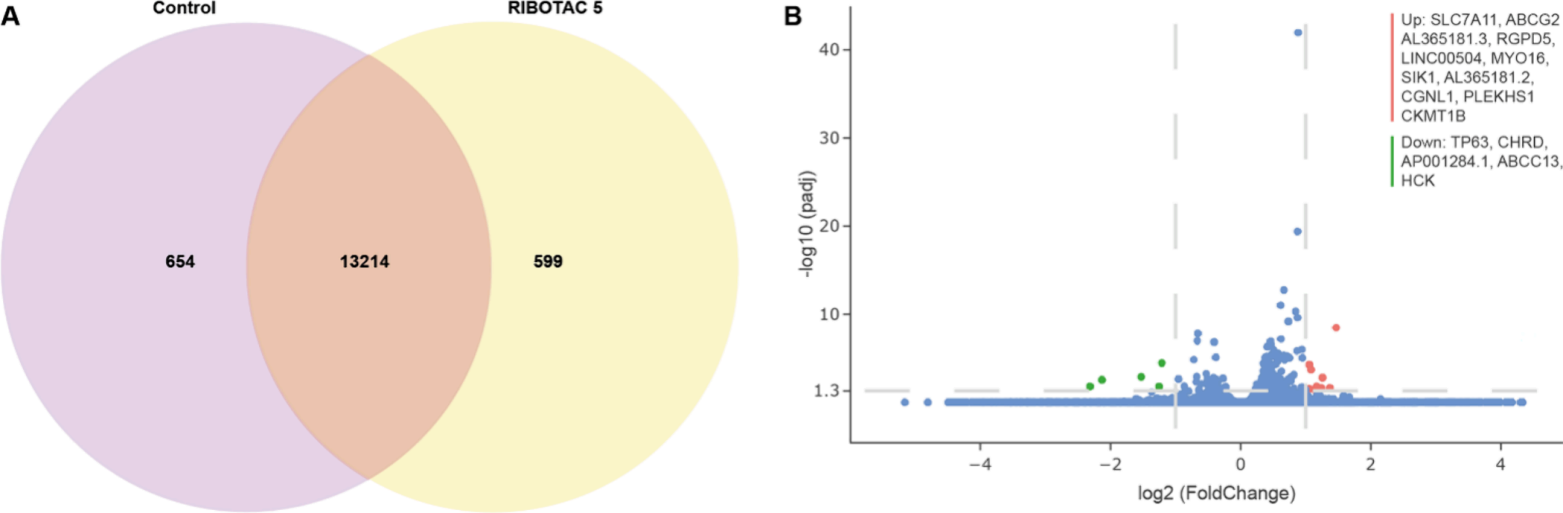
Evaluation of transcriptomic changes in RNaseq upon RIBOTAC 5 treatment. (A) Venn diagram depicting expressed genes in the vehicle control and MCF-7 cells treated with RIBOTAC **5** (10 μM, 48 h). Genes with FPKM ≥ 1 were considered as expressed. Numbers indicate gene counts in each category, with the overlap representing genes detected under both conditions. (B) Volcano plot of differential gene expression between cells treated with RIBOTAC **5** and the vehicle control. Significantly upregulated and downregulated genes were defined using thresholds of |log2 (FoldChange)| ≥ 1 and an adjusted p-value ≤ 0.05 (three biological replicates per condition, *n* = 3). Statistical analysis was performed using the Wald test, adjusting the p-values according to the Benhamini-Hochberg procedure.

Therefore, the clear and diverse cellular morphological changes observed in the cell painting assay could be a collective result of the significantly differentiated genes identified in the RNA-seq analysis, not only for the cancer-associated genes that were significantly downregulated but also for many of the cancer-associated genes that were upregulated. The exact molecular mechanism and involved pathways warrant further investigation and clarification.

## Conclusion

RNA-targeting strategies offer an attractive alternative to the traditional protein-targeting strategies given the expanded targetable genome and the new mechanism of intervention at the post-transcriptional level that will likely yield new biological consequences. In this study, we reported the first example of monovalent G4-*NRAS* binder-based RIBOTAC as *NRAS*-targeting bifunctional molecules to degrade G4-containing *NRAS* mRNA in cancer cells. The following NRAS protein expression level evaluation revealed that the overall NRAS protein expression level was not impacted, which could be explained by the degradation of the low-abundance G4-containing *NRAS* that only accounts for <1% of all *NRAS* transcripts.

RIBOTACs are an emerging class of new bifunctional modality for which limited studies on the cellular modes of action, safety, and toxicity and cellular physicochemical and biological properties are currently available. Therefore, we proceeded with the characterization of the *NRAS*-RIBOTACs in the CPA as an unbiased phenotypic profiling assay. The CPA results measuring cellular morphological changes in the U2OS cells indicated that the RIBOTACs exhibited varied activities based on the different conjugation patterns used to assemble the bifunctional molecules from the monovalent RNase L recruiter MD4 and the monovalent G4-*NRAS* binder JS18 (and the analogous C5). The resulting RIBOTACs showed reduced cytotoxicity in comparison with that of MD4 and improved activity than JS18. Biosimilarity analysis with both established clusters in subprofile analysis and by comparison with reference compounds of annotated targets demonstrated that the RIBOTACs represent a new class of biological cluster with new modes of action.

In parallel with the first demonstration of utilizing the CPA for the fruitful characterization of RIBOTACs, it is acknowledged that it remains a challenge to interpret the high-content CPA data fully to capture all of the covered signals. Apart from the useful information that we deduced from the RIBOTAC CPA data analysis in this study, we expect that with the sophistication of image-based analysis algorithms and the combination with improved machine-learning methods, even more biologically meaningful data can be extracted to further assist the study of new chemical modalities including RIBOTACs and PROTACs.

## Experimental Section

### Cell Lines

MDA-MB-231 cells (derived from 51-year-old white, female with adenocarcinoma) and MCF-7 cells (derived from 69-year-old white, female with adenocarcinoma) were cultivated in high-glucose Dulbecco’s modified Eagle’s medium (DMEM) with preadded GlutaMAX supplement and 10% fetal bovine serum at 37 °C in 5% CO_2_ in a humidified incubator.

### RT-qPCR Measurement

MDA-MB-231 and MCF-7 cells were seeded in a 6-well culture plate at different densitiescorresponding to exposure time frames (MDA-MB-231: 24 h–1·10^5^ cells/mL, 48 h–7·10^4^ cells/mL, 72 h–3·10^4^ cells/mL). After 24 h incubation, fresh medium containing different concentrations of Ribotacs (1 or 10 μM) or DMSO (vehicle control) were added to the cells and incubated for 24 h, 48 h, or 72 h. Isolation of total RNA was performed using the Rneasy Mini Kit (Qiagen) and DNA was removed via the Rnase-Free Dnase Set (Qiagen) according to the manufacturer’s instructions. 500 ng of total RNA were used for cDNA synthesis utilizing the High-Capacity cDNA Reverse Transcription Kit (Applied Biosystems). The reactions were performed for 10 min at 25 °C and 2 h at 37 °C followed by 5 min at 85 °C and then held at 4 °C. Four ng of cDNA were used for RT-qPCR using PowerUp SYBR Green Master Mix (Applied Biosystems) in a 7500 Fast Real-Time PCR System (Applied Biosystems) with appropriate primers (shown in Table S5). The reactions were incubated at 50 °C for 2 min and 95 °C for 2 min, followed by 40 cycles of 95 °C for 15 s and 60 °C for 1 min. Relative gene expression was determined using the 2^–ΔΔC^_T_-method^42^ normalizing the samples to the relative mRNA levels in the vehicle control. The calculation of ΔΔC_T_ between each gene of interest and the mean of control samples was performed as follows: ΔC_T_ = C_T_ (*NRAS*) – mean C_T_ (GAPDH, β-Actin); ΔΔC_T_ = ΔC_T_ (treatment) – ΔC_T_ (control); Rel. Gene Expression = 2^–ΔΔC^_T_.

### RNA-seq

Input samples from RT-qPCR experiments were used to analyze the transcriptional profile of MCF-7 cells treated with either 10 μM of **5** or vehicle control (0.1% DMSO) for 48 h (n = 3). Ribosomal RNA was depleted using the QIAseq FastSelect -rRNA HMR Kit (Qiagen) and total RNA libraries were prepared using the QIAseq Stranded RNA Library UDI Kit (Qiagen) with the QIAseq UDI Y-Adapter Kit (Qiagen) according to the manufacturer’s instructions. Total RNA libraries were sequenced by Novogene using an Illumina NovaSeq 6000 platform according to established protocols. Bioinformatic processing was performed by Novogene and Figures were created using the NovoMagic platform.

### Western Blot

MCF-7 cells were seeded in six-well culture plates at a density of 1 × 10^6^ cells per mL. After 24 h of incubation fresh medium containing different doses of compounds (1, 10 μM) or DMSO (vehicle control) was added to the cells and incubated for 48 h. For protein extraction, cells were lysed using RIPA buffer (25 mM Tris, 150 mM NaCl, 1% (v/v) NP-40, 0.5% (w/v) sodium deoxycholate, 0.1% (w/v) SDS, cOmplete protease inhibitor EDTA free) for 30 min on ice followed by centrifugation at x g and 4 °C for 25 min. The amount of protein was determined by using the Pierce BCA Protein Assay (Thermo Scientific). Twelve μg of protein was loaded into each well of a 10% SDS-acrylamide gel and electrophoresed at 170 V for ∼60 min. The transfer was performed at 4 °C and 90 V for 90 min or at 30 V and 4 °C overnight onto a PVDF-membrane. Blots were blocked with 5% milk in 1x TBST for 1 h at RT or overnight at 4 °C, followed by three washing steps with 1x TBST for 5 min each. NRAS and Tubulin proteins were detected by incubating with an NRAS (Invitrogen 703435; 1:1000) or a Tubulin antibody (Sigma-Aldrich T6199; 1:1000) at RT for 1 h. Following three washing steps with 1x TBST for 5 min, Horseradish peroxidase-conjugated rabbit antimouse IgG for Tubulin (Sigma-Aldrich A9044; 1:10000) or goat antirabbit IgG for NRAS (Sigma-Aldrich A0545; 1:10000). After three washing steps with 1x TBST for 5 min, Visualization of proteins was performed using ECL Prime Western Blotting Detection Reagent (Cytiva) in a Bio-Rad ChemiDoc MP imaging System.

### Cell Viability Assay

MCF-7 cells and MDA-MB-231 cells were seeded in 96-well culture plates at a density of 5·10^4^ cells/mL (for MCF-7 cells) or 3.5·10^4^ cells/mL (for MDA-MB-231 cells). After 24 h incubation, varying concentrations of compounds (ranging between 0.46 and 46 μM) or DMSO (vehicle control) were added to the cells and incubated for 72 h. Spent medium was aspirated and replaced with 110 μL of CCK-8 dilution (10:1 medium: CCK-8 reagent; Vazyme), and incubated again at 37 °C with 5% CO_2_ for 4 h. Following incubation, the absorbance was measured at a wavelength of 450 nm. A blank measurement of the wells with 110 μL of CCK-8 dilution was taken and subtracted. Relative cell viability was calculated by normalizing against an untreated vehicle control.

### Cell Painting Assay

The cell painting assay was performed following the previously described methods.^31,32^ Initially, 5 μL U2OS medium were added to each well of a 384-well plate (Revvity PhenoPlate 384). Subsequently, U2OS cells were seeded at a density of 1600 cells per well in 20 μL medium. The plate was incubated for 10 min at ambient temperature, followed by an additional 4 h of incubation (37 °C, 5% CO2). Compound treatment was performed with the Echo 520 acoustic dispenser (Beckman-Coulter) at final concentrations of 10, 3, or 1 μM. Incubation with compound was performed for 20 h (37 °C, 5% CO2). Subsequently, mitochondria were stained with Mito Tracker Deep Red (Thermo Fisher Scientific, Cat. No. M22426). The Mito Tracker Deep Red stock solution (1 mM) was diluted to a final concentration of 100 nM in a prewarmed medium. The medium was removed from the plate leaving 10 μL residual volume and 25 μL of the Mito Tracker solution were added to each well. The plate was incubated for 30 min in the dark (37 °C, 5% CO2). To fix the cells 7 μL of 18.5% formaldehyde in PBS were added, resulting in a final formaldehyde concentration of 3.7%. Subsequently, the plate was incubated for another 20 min in darkness (RT) and washed three times with 70 μL of PBS. (Agilent Washer Elx405). Cells were permeabilized by addition of 25 μL of 0.1% Triton X-100 to each well, followed by 15 min incubation (RT) in darkness. The cells were washed three times with PBS leaving a final volume of 10 μL. To each well 25 μL of a staining solution were added, which contains 1% BSA, 5 μL/ml Phalloidin (Alexa594 conjugate, Thermo Fisher Scientific, A12381), 25 μg/mL Concanavalin A (Alexa488 conjugate, Thermo Fisher Scientific, Cat. No. C11252), 5 μg/mL Hoechst 33342 (Sigma, Cat. No. B2261–25 mg), 1.5 μg/mL WGA-Alexa594 conjugate (Thermo Fisher Scientific, Cat. No. No. W11262) and 1.5 μM SYTO 14 solution (Thermo Fisher Scientific, Cat. No. S7576). The plate is incubated for 30 min (RT) in darkness and washed three times with 70 μL of PBS. After the final washing step, the PBS was not aspirated. The plates were sealed and centrifuged for 1 min at 50*g*. The plates were prepared in triplicates with shifted layouts to reduce plate effects and imaged using a Micro XL High-Content Screening System (Molecular Devices) in 5 channels (DAPI: Ex350–400/Em410–480; FITC: Ex470–500/Em510–540; Spectrum Gold: Ex520–545/Em560–585; TxRed: Ex535–585/Em600–650; Cy5: Ex605–650/Em670–715) with 9 sites per well and 20× magnification (binning 2). The generated images were processed with the *CellProfiler* package (https://cellprofiler.org/, version 3.0.0) on a computing cluster of the Max Planck Society to extract 1716 cell features per microscope site. The data was then further aggregated as medians per well (9 sites → 1 well), then over the three replicates. Further analysis was performed with custom *Python* (https://www.python.org/) scripts using the *Pandas* (https://pandas.pydata.org/) and *Dask* (https://dask.org/) data processing libraries as well as the *Scientific Python* (https://scipy.org/) package (separate publication to follow).

From the total set of 1716 features, a subset of highly reproducible and robust features was determined using the procedure described by Woehrmann et al.^43^ Two biological repeats of one plate containing reference compounds were analyzed. For every feature, the full profile over each whole plate was calculated. If the profiles from the two repeats showed a similarity of no less than 0.8 (see below), the feature was added to the set. This procedure was performed only once and resulted in a set of 579 robust features out of a total of 1716 that were used for all further analyses. The phenotypic profiles were compiled from the Z-scores of all individual cellular features, where the Z-score is a measure of how far away a data point is from a median value. Specifically, Z-scores of test compounds were calculated relative to the Median values of DMSO controls. The phenotypic compound profile is then determined as the list of Z-scores of all features for one compound. In addition to the phenotypic profile, an induction value was determined for each compound as the fraction of significantly changed features in percent. Similarities of phenotypic profiles (termed *Biosimilarity*) were calculated from the correlation distances (CD) between two profiles (https://docs.scipy.org/doc/scipy/ reference/generated/scipy.spatial.distance.correlation.html). The Biosimilarity is then defined as *Biosimilarity* = 1 – CD. Biosimilarity values smaller than 0 are set to 0, and the Biosimilarity is expressed in percent (0–100).

## Supplementary Material



## Notes

General chemistry information, synthetic procedures, compound characterization data, ^1^H and ^13^C NMR spectra, and UPLC-MS spectra (PDF)
