# Supplementary material for: NRAS mRNA-Degrading Bifunctional Small Molecules Induce Diverse Cellular Morphological Changes in Cancer Cells
Source: JACS Au. 2026 May 11;6(5):2730–42. doi: 10.1021/jacsau.5c01600 (PMC13213484; doi:10.1021/jacsau.5c01600)
Supplement: Supplementary file 1 [file au5c01600_si_001.pdf]

Supporting Information

NRAS mRNA-Degrading Bifunctional Small Molecules Induce Diverse Cellular Morphological Changes in Cancer Cells

Mao Jiang,[1-3,#] Daniel Hösle,[1-3,#] Yang Liu,[1-3] Sonja Sievers,[2,4] and Peng Wu[1-3,*]

[1]Chemical Genomics Centre, Max Planck Institute of Molecular Physiology, Dortmund 44227, Germany

[2]Department of Chemical Biology, Max Planck Institute of Molecular Physiology, Dortmund 44227, Germany

[3]Faculty of Chemistry and Chemical Biology, TU Dortmund University, Dortmund 44227, Germany

[4]Compound Management and Screening Center, Dortmund 44227, Germany

[#]Equally contributed authors

*Corresponding Author:

P. Wu, email: peng.wu@mpi-dortmund.mpg.de

Table of Contents

Supplementary Figures S1-S6…..……………………………………….... S3

Supplementary Tables S1-S5…...………………………………………..... S9

General Chemical Information………………………………………... S31

Synthetic Procedures and Compound Characterization Data ……..… S32

NMR Spectra………………………………………………………………. S64

UPLC-MS Spectra………………………………………………………… S74

References………………………………………………………………… S80

Supplementary Figures

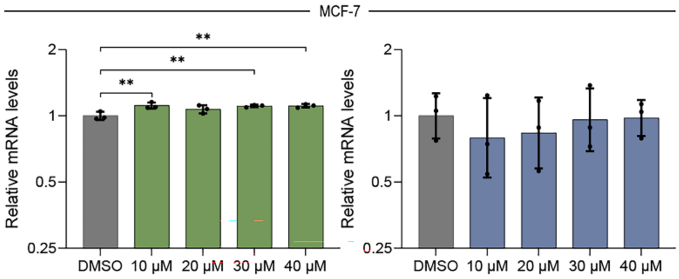

Figure S1. Testing RIBOTAC 5 in RT-qPCR with varied concentrations (10µM and 40 µM) in MCF-7 cells. The RT-qPCR data are shown as geometric mean ± geometric SD (three independent biological replicates). Statistical significance was determined using a one-way ANOVA followed by Dunnett's post-hoc test on ΔC_t values (*$p < 0.05$, **$p < 0.01$).

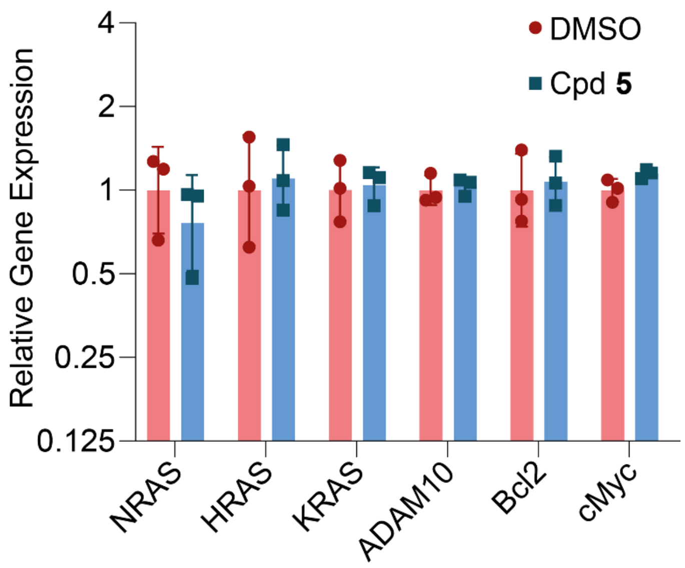

Figure S2. Impact of RIBOTAC **5** on the expression levels of different G4-containing transcripts in MCF-7 cells. Relative mRNA levels were measured by RT-qPCR and normalized to the mean of GAPDH and β-Actin using the $2^{-\Delta\Delta Ct}$ method. Data are expressed as the geometric mean ± geometric SD (three independent biological replicates). Statistical significance was determined by performing multiple unpaired t-tests on the ΔCt values. To account for multiple comparisons, p-values (listed in Table S3) were adjusted using the Holm-Šídák method.

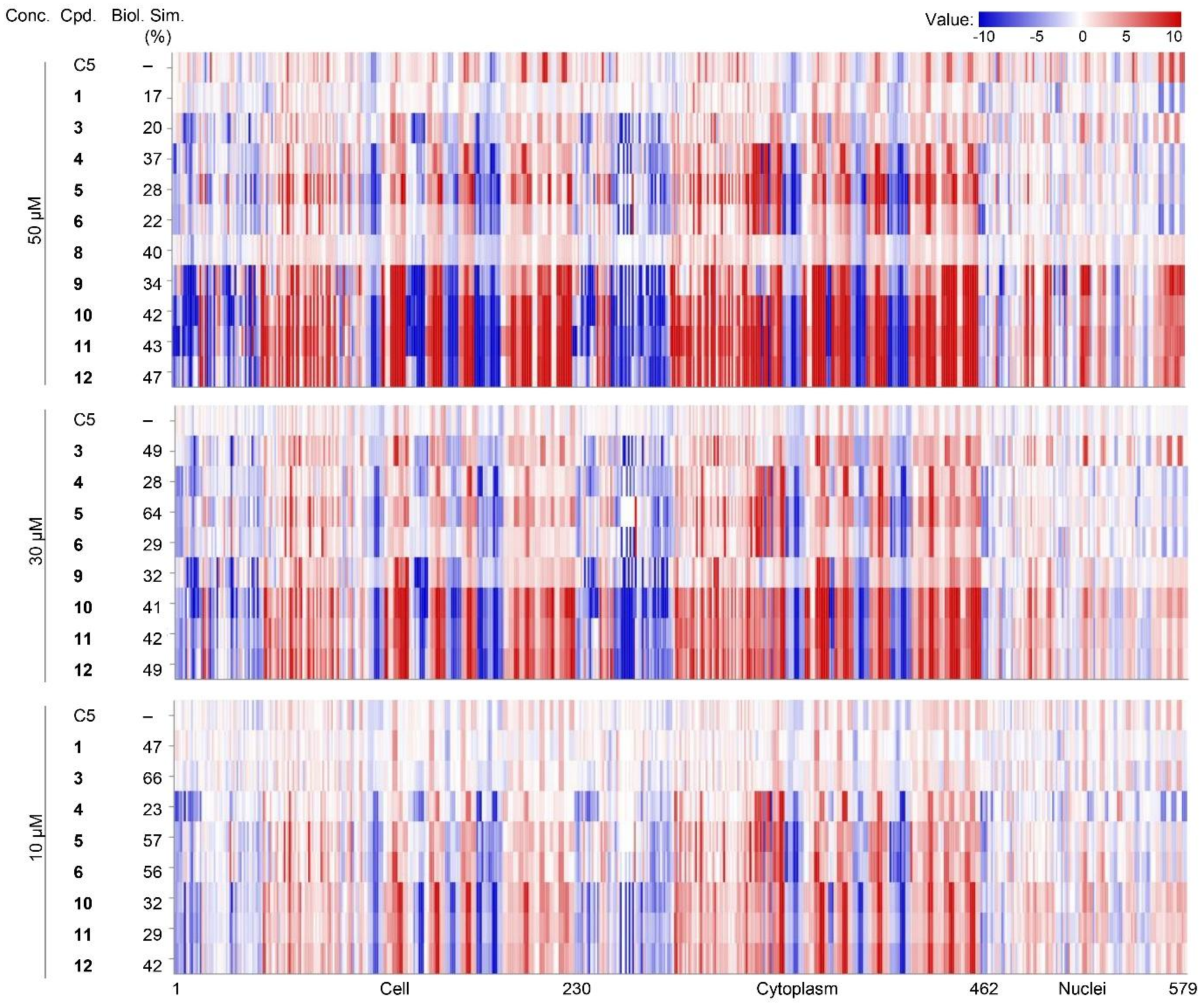

Figure S3. Heatmap show of the cell painting assay results for the RIBOTACs and the biosimilarity of the tested compounds. The top line of the fingerprint of the heatmap is the result for compound C5, which is set as a reference fingerprint (100% biological simiarity), to which the following RIBOTACs are compared for their biosimilarity (Biol. Sim., %). C5 and each of the RIBOTACs were tested in three concentrations (Conc.): 50 µM, 30 µM, and 10 µM. The fingerprints were generated only for compounds that showed an induction of > 5% (Figure 5). The set of 579 parameters is divided into parameters (the y-axis) related to the cell (1-229), cytoplasm (230-461), and nuclei (462-579). Decreased parameters are shown in blue, and increased parameters are shown in red.

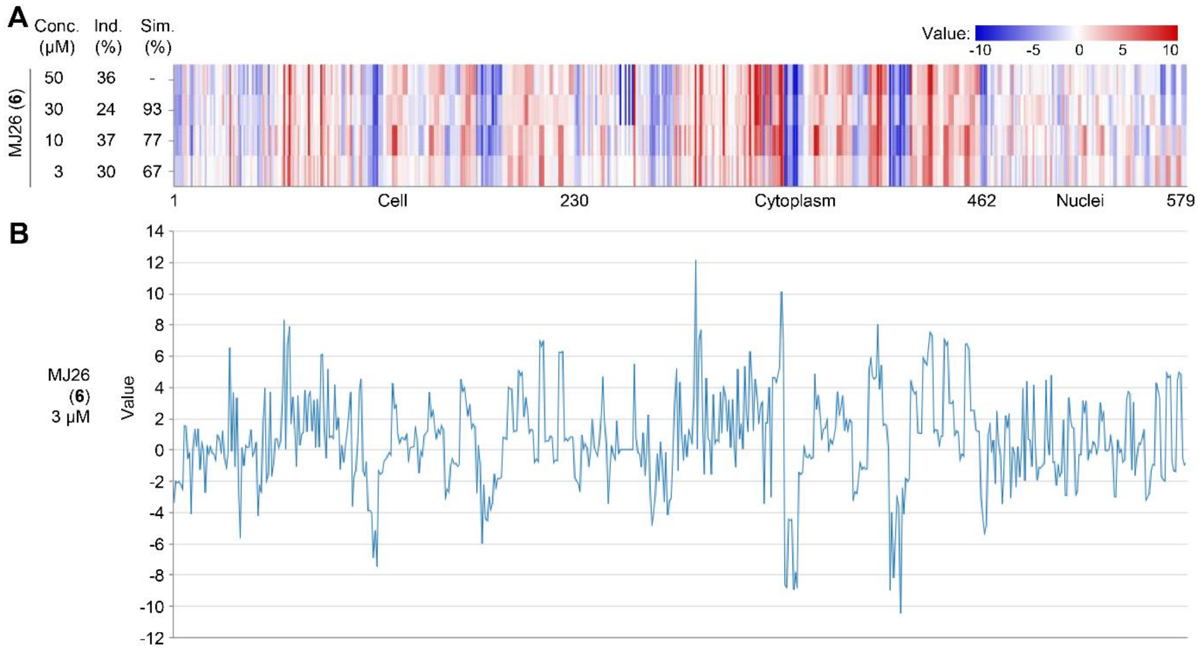

Figure S4. Cell painting results showing the biological similarities of MJ26 (**6**) tested in different concentrations in U2OS cells. (A) The top line of the fingerprint of the heatmap is the result for MJ26 tested in 50 µM, which is set as a reference fingerprint (100% biological similarity), to which the following testing results at reduced concentrations (30 µM, 10 µM, and 3 µM) are compared for their biological similarity (Biol. Sim. %). (B) The line plot showing the individual changes of the 579 parameters for MH26 tested in 3 µM. Positive values correspond to the ones shown in red in the fingerprint of the above heatmap (such as parameter No. 299 for cytoplasm intensity of ER, value 12.14; parameter No. 348 for cytoplasm radial distribution of Golgi, value 10.1); negative values correspond to the ones shown in blue in the fingerprint of the above heatmap (such as parameter No. 416 for cytoplasm inverse difference moment of Golgi, value -10.49).

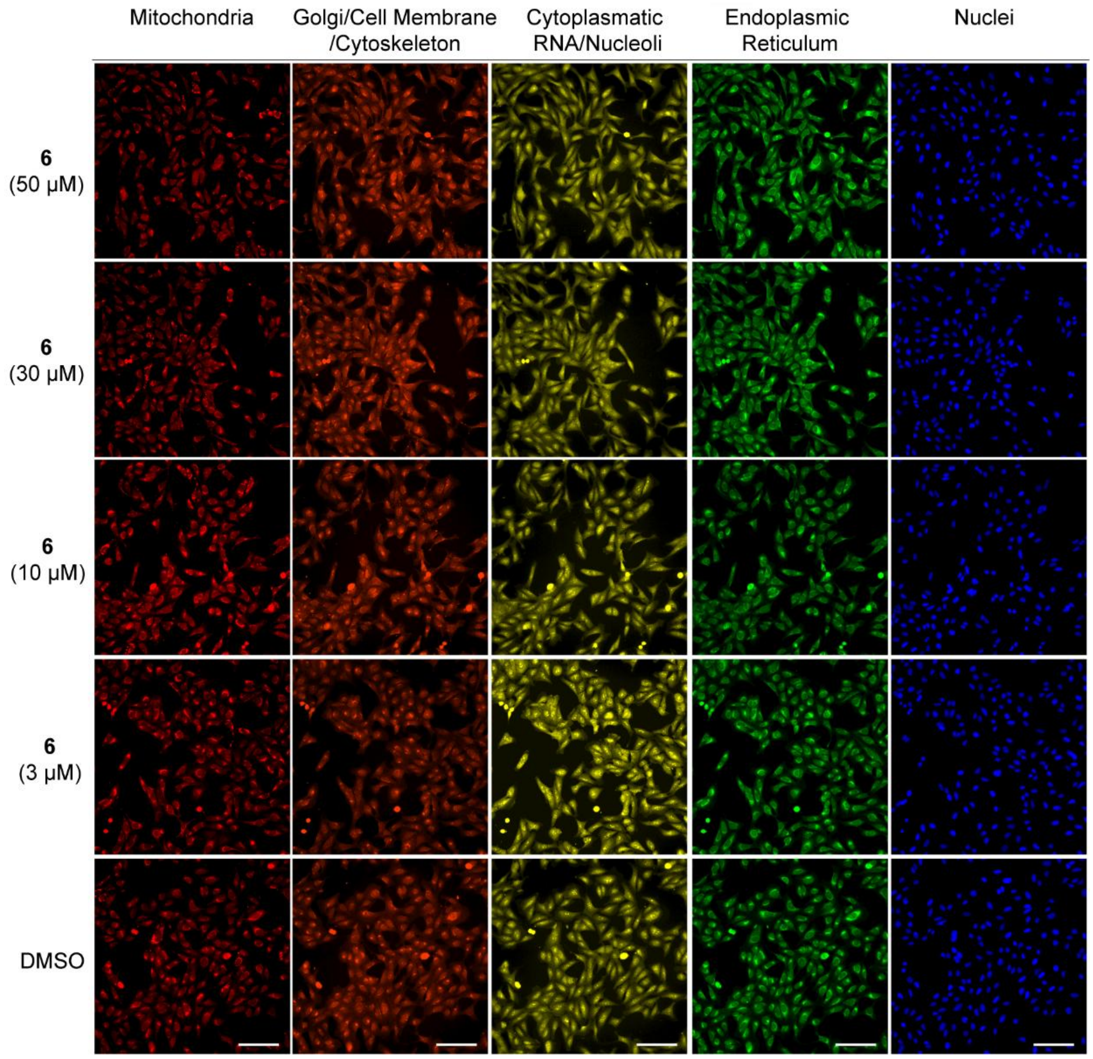

Figure S5. Microscope images from the CPA analysis in U2OS cells upon treating with the RIBOTAC **6** at different concentrations (50 µM, 30 µM, 10 µM, 3 µM). DMSO was used as the control. Scale bar, 150 µm for all images.

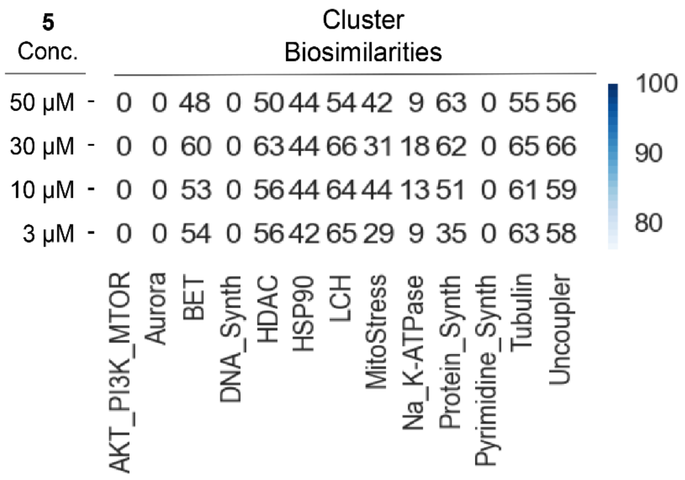

Figure S6. Biosimilarity of RIBOTAC **5** with established clusters in the subprofile analysis at different concentrations. The 13 established clusters are associated with AKT/PI3 K/MTOR, aurora kinase, bromodomain and extra-terminal domain (BET), DNA synthesis, histone deacetylase (HDAC), heat shock protein 90 (HSP90), lysosomotropism/cholesterol homeostasis (LCH) regulation, mitochondrial stress regulation, Na+/K+ ATPases, protein synthesis, de novo pyrimidine biosynthesis, tubulin, and uncoupling of the mitochondrial proton gradient.

Supplementary Tables

Table S1. Adjusted p-values for the RIBOTACs 1–12 in qPCR (associated data for Figure 3A and 3B)

Cpds	Conc.	Adjusted p-values NRAS	Adjusted p-values G4 NRAS
**1**	1 µM	0.3341	0.9999
	10 µM	0.7585	0.7885
**2**	1 µM	0.9417	0.9426
	10 µM	0.8029	0.9974
**3**	1 µM	0.8653	>0.9999
	10 µM	>0.9999	0.9991
**4**	1 µM	>0.9999	0.9954
	10 µM	0.9963	>0.9999
**5**	1 µM	0.3111	0.8973
	10 µM	0.6185	0.3812
**6**	1 µM	0.9494	0.4326
	10 µM	0.3162	0.8887
**7**	1 µM	0.0947	0.8541
	10 µM	0.4659	0.5128
**8**	1 µM	0.9363	0.9882
	10 µM	0.0455	0.9630
**9**	1 µM	>0.9999	>0.9999
	10 µM	0.9994	0.9998
**10**	1 µM	>0.9999	0.9986
	10 µM	>0.9999	>0.9999
**11**	1 µM	0.9998	>0.9999
	10 µM	>0.9999	>0.9999
**12**	1 µM	0.9999	0.9470
	10 µM	0.1745	0.9886
MD4	1 µM	0.0320	>0.9999
	10 µM	0.4502	0.9372
JS18	1 µM	0.1087	0.6840
	10 µM	0.1088	0.8030

Table S2. Adjusted p-values for selected RIBOTACs in qPCR (associated data for Figure S1)

RIBOTAC 5	MCF-7, 48 h	
	NRAS	G4-NRAS
**10 μM**	0.0072	0.7897
**20 μM**	0.0788	0.8931
**30 μM**	0.0095	0.9995
**40 μM**	0.0085	>0.9999

Table S3. Adjusted p-values for RIBOTAC 5 in qPCR evaluating selectivity of different G4 transcripts (associated data for Figure S2)

G4 transcripts	Adjusted p-values
NRAS	0.939308
HRAS	0.984841
KRAS	0.984841
ADAM10	0.943431
Bcl2	0.984841
cMyc	0.562201

Table S4. The 579 parameters measured in CPA for RIBOTAC 5 (associated data for Figure 6B)

Number of Parameter, Feature Description, Value showing the individual change of the parameter

1,Median_Cells_AreaShape_Area,-4.094

2,Median_Cells_AreaShape_MaximumRadius,-2.039

3,Median_Cells_AreaShape_MeanRadius,-2.326

4,Median_Cells_AreaShape_MinFeretDiameter,-4.165

5,Median_Cells_AreaShape_MinorAxisLength,-2.121

6,Median_Cells_AreaShape_Perimeter,-1.478

7,Median_Cells_Correlation_Correlation_ER_Ph_golgi,1.114

8,Median_Cells_Correlation_Correlation_ER_Syto,1.762

9,Median_Cells_Correlation_Correlation_Hoechst_ER,-0.729

10,Median_Cells_Correlation_Correlation_Hoechst_Mito,-1.377

11,Median_Cells_Correlation_Correlation_Hoechst_Ph_golgi,-2.594

12,Median_Cells_Correlation_Correlation_Hoechst_Syto,-2.406

13,Median_Cells_Correlation_Correlation_Mito_ER,1.313

14,Median_Cells_Correlation_Correlation_Mito_Ph_golgi,0.357

15,Median_Cells_Correlation_Correlation_Mito_Syto,0.258

16,Median_Cells_Correlation_Correlation_Syto_Ph_golgi,-0.259

17,Median_Cells_Correlation_K_ER_Syto,-0.101

18,Median_Cells_Correlation_K_Hoechst_Syto,0.087

19,Median_Cells_Correlation_K_Mito_Hoechst,-1.867

20,Median_Cells_Correlation_K_Ph_golgi_Syto,-0.187

21,Median_Cells_Correlation_K_Syto_ER,0.166

22,Median_Cells_Correlation_K_Syto_Hoechst,-0.197

23,Median_Cells_Correlation_K_Syto_Ph_golgi,0.164

24,Median_Cells_Correlation_Manders_ER_Hoechst,-0.686

25,Median_Cells_Correlation_Manders_ER_Syto,0.0

26,Median_Cells_Correlation_Manders_Mito_Hoechst,0.437

27,Median_Cells_Correlation_Manders_Ph_golgi_Hoechst,0.459

28,Median_Cells_Correlation_Manders_Syto_Hoechst,0.069

29,Median_Cells_Correlation_Overlap_Hoechst_ER,0.359

30,Median_Cells_Correlation_Overlap_Hoechst_Mito,-1.181

31,Median_Cells_Correlation_Overlap_Hoechst_Ph_golgi,-1.591

32,Median_Cells_Correlation_Overlap_Hoechst_Syto,-1.548

33,Median_Cells_Correlation_Overlap_Mito_ER,5.65

34,Median_Cells_Correlation_Overlap_Mito_Ph_golgi,-0.427

35,Median_Cells_Correlation_Overlap_Mito_Syto,2.72

36,Median_Cells_Correlation_Overlap_Syto_Ph_golgi,0.681

37,Median_Cells_Correlation_RWC_ER_Mito,1.216

38,Median_Cells_Correlation_RWC_Hoechst_ER,-2.474

39,Median_Cells_Correlation_RWC_Hoechst_Mito,-0.819

40,Median_Cells_Correlation_RWC_Hoechst_Ph_golgi,-0.844

41,Median_Cells_Correlation_RWC_Hoechst_Syto,-1.042

42,Median_Cells_Correlation_RWC_Mito_Hoechst,0.262

43,Median_Cells_Correlation_RWC_Mito_Syto,0.417

44,Median_Cells_Correlation_RWC_Ph_golgi_Hoechst,0.442

45,Median_Cells_Correlation_RWC_Ph_golgi_Mito,-0.326

46,Median_Cells_Correlation_RWC_Ph_golgi_Syto,0.016

47,Median_Cells_Correlation_RWC_Syto_Hoechst,-0.062

48,Median_Cells_Correlation_RWC_Syto_Mito,0.385

49,Median_Cells_Granularity_1_Mito,-2.693

50,Median_Cells_Granularity_1_Ph_golgi,-1.333

51,Median_Cells_Granularity_1_Syto,-1.12

52,Median_Cells_Granularity_2_Mito,1.221

53,Median_Cells_Granularity_2_Ph_golgi,2.591

54,Median_Cells_Granularity_2_Syto,-0.44

55,Median_Cells_Granularity_3_ER,-0.406

56,Median_Cells_Granularity_3_Mito,2.951

57,Median_Cells_Granularity_3_Ph_golgi,-0.101

58,Median_Cells_Granularity_3_Syto,-0.059

59,Median_Cells_Granularity_4_Mito,0.952

60,Median_Cells_Granularity_5_Mito,1.552

61,Median_Cells_Intensity_IntegratedIntensityEdge_Hoechst,-0.73

62,Median_Cells_Intensity_IntegratedIntensity_Syto,-0.19

63,Median_Cells_Intensity_LowerQuartileIntensity_Mito,2.021

64,Median_Cells_Intensity_MADIntensity_ER,7.783

65,Median_Cells_Intensity_MADIntensity_Hoechst,0.0

66,Median_Cells_Intensity_MADIntensity_Mito,2.802

67,Median_Cells_Intensity_MADIntensity_Ph_golgi,8.897

68,Median_Cells_Intensity_MADIntensity_Syto,0.606

69,Median_Cells_Intensity_MaxIntensityEdge_Mito,1.945

70,Median_Cells_Intensity_MaxIntensityEdge_Syto,0.647

71,Median_Cells_Intensity_MaxIntensity_Hoechst,-0.232

72,Median_Cells_Intensity_MaxIntensity_Mito,2.308

73,Median_Cells_Intensity_MaxIntensity_Ph_golgi,0.102

74,Median_Cells_Intensity_MeanIntensityEdge_Hoechst,0.569

75,Median_Cells_Intensity_MeanIntensity_Hoechst,0.82

76,Median_Cells_Intensity_MeanIntensity_Mito,2.493

77,Median_Cells_Intensity_MeanIntensity_Syto,0.502

78,Median_Cells_Intensity_MedianIntensity_ER,3.384

79,Median_Cells_Intensity_MedianIntensity_Mito,2.33

80,Median_Cells_Intensity_MedianIntensity_Syto,0.561

81,Median_Cells_Intensity_MinIntensityEdge_Mito,0.922

82,Median_Cells_Intensity_MinIntensityEdge_Syto,0.68

83,Median_Cells_Intensity_MinIntensity_Mito,0.996

84,Median_Cells_Intensity_MinIntensity_Syto,0.706

85,Median_Cells_Intensity_StdIntensityEdge_ER,5.166

86,Median_Cells_Intensity_StdIntensityEdge_Mito,2.326

87,Median_Cells_Intensity_StdIntensityEdge_Syto,0.544

88,Median_Cells_Intensity_StdIntensity_Hoechst,-0.347

89,Median_Cells_Intensity_StdIntensity_Mito,1.748

90,Median_Cells_Intensity_StdIntensity_Ph_golgi,0.784

91,Median_Cells_Intensity_StdIntensity_Syto,0.18

92,Median_Cells_Intensity_UpperQuartileIntensity_Hoechst,1.19

93,Median_Cells_Intensity_UpperQuartileIntensity_Mito,2.727

94,Median_Cells_Intensity_UpperQuartileIntensity_Syto,0.528

95,Median_Cells_RadialDistribution_FracAtD_Mito_3of4,2.672

96,Median_Cells_RadialDistribution_FracAtD_Mito_4of4,-1.407

97,Median_Cells_RadialDistribution_FracAtD_Ph_golgi_1of4,-2.418

98,Median_Cells_RadialDistribution_FracAtD_Ph_golgi_2of4,-1.327

99,Median_Cells_RadialDistribution_FracAtD_Ph_golgi_4of4,0.157

100,Median_Cells_RadialDistribution_MeanFrac_Mito_1of4,1.158

101,Median_Cells_RadialDistribution_MeanFrac_Mito_2of4,1.321

102,Median_Cells_RadialDistribution_MeanFrac_Mito_3of4,2.204

103,Median_Cells_RadialDistribution_MeanFrac_Mito_4of4,-2.082

104,Median_Cells_RadialDistribution_MeanFrac_Ph_golgi_1of4,-0.986

105,Median_Cells_RadialDistribution_MeanFrac_Ph_golgi_2of4,-0.204

106,Median_Cells_RadialDistribution_MeanFrac_Ph_golgi_4of4,-0.495

107,Median_Cells_RadialDistribution_RadialCV_Mito_3of4,0.749

108,Median_Cells_RadialDistribution_RadialCV_Mito_4of4,2.572

109,Median_Cells_RadialDistribution_RadialCV_Ph_golgi_1of4,-0.362

110,Median_Cells_RadialDistribution_RadialCV_Ph_golgi_2of4,-0.231

111,Median_Cells_RadialDistribution_RadialCV_Ph_golgi_3of4,-0.353

112,Median_Cells_Texture_AngularSecondMoment_Mito_10_00,-2.081

113,Median_Cells_Texture_AngularSecondMoment_Mito_3_00,-1.878

114,Median_Cells_Texture_AngularSecondMoment_Mito_5_00,-1.995

115,Median_Cells_Texture_AngularSecondMoment_Ph_golgi_10_00,-7.228

116,Median_Cells_Texture_AngularSecondMoment_Ph_golgi_3_00,-4.963

117,Median_Cells_Texture_AngularSecondMoment_Ph_golgi_5_00,-7.371

118,Median_Cells_Texture_AngularSecondMoment_Syto_10_00,-0.496

119,Median_Cells_Texture_AngularSecondMoment_Syto_3_00,-0.552

120,Median_Cells_Texture_AngularSecondMoment_Syto_5_00,-0.603

121,Median_Cells_Texture_Contrast_ER_3_00,-1.27

122,Median_Cells_Texture_Contrast_ER_5_00,-1.232

123,Median_Cells_Texture_Contrast_Hoechst_10_00,-0.051

124,Median_Cells_Texture_Contrast_Hoechst_3_00,-0.594

125,Median_Cells_Texture_Contrast_Hoechst_5_00,-0.675

126,Median_Cells_Texture_Contrast_Mito_10_00,1.542

127,Median_Cells_Texture_Contrast_Mito_3_00,0.734

128,Median_Cells_Texture_Contrast_Mito_5_00,0.714

129,Median_Cells_Texture_Contrast_Ph_golgi_10_00,1.354

130,Median_Cells_Texture_Contrast_Ph_golgi_3_00,0.534

131,Median_Cells_Texture_Contrast_Ph_golgi_5_00,0.807

132,Median_Cells_Texture_Contrast_Syto_10_00,0.453

133,Median_Cells_Texture_Contrast_Syto_3_00,0.965

134,Median_Cells_Texture_Contrast_Syto_5_00,1.059

135,Median_Cells_Texture_Correlation_ER_10_00,0.409

136,Median_Cells_Texture_Correlation_ER_3_00,0.425

137,Median_Cells_Texture_Correlation_ER_5_00,0.514

138,Median_Cells_Texture_Correlation_Mito_10_00,0.601

139,Median_Cells_Texture_Correlation_Mito_3_00,1.389

140,Median_Cells_Texture_Correlation_Mito_5_00,1.064

141,Median_Cells_Texture_Correlation_Ph_golgi_10_00,-0.991

142,Median_Cells_Texture_Correlation_Ph_golgi_3_00,0.089

143,Median_Cells_Texture_Correlation_Ph_golgi_5_00,-0.384

144,Median_Cells_Texture_Correlation_Syto_10_00,-0.505

145,Median_Cells_Texture_Correlation_Syto_5_00,0.938

146,Median_Cells_Texture_DifferenceEntropy_Hoechst_10_00,1.179

147,Median_Cells_Texture_DifferenceEntropy_Mito_10_00,1.529

148,Median_Cells_Texture_DifferenceEntropy_Mito_3_00,0.737

149,Median_Cells_Texture_DifferenceEntropy_Mito_5_00,0.798

150,Median_Cells_Texture_DifferenceEntropy_Ph_golgi_10_00,1.793

151,Median_Cells_Texture_DifferenceEntropy_Ph_golgi_3_00,0.824

152,Median_Cells_Texture_DifferenceEntropy_Ph_golgi_5_00,1.41

153,Median_Cells_Texture_DifferenceEntropy_Syto_10_00,0.438

154,Median_Cells_Texture_DifferenceEntropy_Syto_3_00,0.657

155,Median_Cells_Texture_DifferenceEntropy_Syto_5_00,0.597

156,Median_Cells_Texture_DifferenceVariance_Mito_10_00,-2.559

157,Median_Cells_Texture_DifferenceVariance_Mito_3_00,-2.012

158,Median_Cells_Texture_DifferenceVariance_Mito_5_00,-2.308

159,Median_Cells_Texture_DifferenceVariance_Ph_golgi_10_00,-1.196

160,Median_Cells_Texture_DifferenceVariance_Ph_golgi_3_00,-0.602

161,Median_Cells_Texture_DifferenceVariance_Ph_golgi_5_00,-1.025

162,Median_Cells_Texture_DifferenceVariance_Syto_10_00,0.0

163,Median_Cells_Texture_DifferenceVariance_Syto_3_00,-0.585

164,Median_Cells_Texture_DifferenceVariance_Syto_5_00,-0.577

165,Median_Cells_Texture_Entropy_Mito_10_00,2.148

166,Median_Cells_Texture_Entropy_Mito_3_00,2.0

167,Median_Cells_Texture_Entropy_Mito_5_00,2.06

168,Median_Cells_Texture_Entropy_Ph_golgi_10_00,3.237

169,Median_Cells_Texture_Entropy_Ph_golgi_3_00,2.38

170,Median_Cells_Texture_Entropy_Ph_golgi_5_00,2.956

171,Median_Cells_Texture_Entropy_Syto_10_00,0.367

172,Median_Cells_Texture_Entropy_Syto_3_00,0.512

173,Median_Cells_Texture_Entropy_Syto_5_00,0.451

174,Median_Cells_Texture_InfoMeas2_Ph_golgi_10_00,-3.537

175,Median_Cells_Texture_InfoMeas2_Ph_golgi_3_00,0.47

176,Median_Cells_Texture_InfoMeas2_Ph_golgi_5_00,-0.793

177,Median_Cells_Texture_InverseDifferenceMoment_ER_10_00,-4.778

178,Median_Cells_Texture_InverseDifferenceMoment_ER_3_00,-1.936

179,Median_Cells_Texture_InverseDifferenceMoment_ER_5_00,-3.937

180,Median_Cells_Texture_InverseDifferenceMoment_Mito_10_00,-2.13

181,Median_Cells_Texture_InverseDifferenceMoment_Mito_3_00,-0.718

182,Median_Cells_Texture_InverseDifferenceMoment_Mito_5_00,-1.132

183,Median_Cells_Texture_InverseDifferenceMoment_Ph_golgi_10_00,-3.975

184,Median_Cells_Texture_InverseDifferenceMoment_Ph_golgi_3_00,-1.754

185,Median_Cells_Texture_InverseDifferenceMoment_Ph_golgi_5_00,-2.974

186,Median_Cells_Texture_InverseDifferenceMoment_Syto_10_00,-0.771

187,Median_Cells_Texture_InverseDifferenceMoment_Syto_3_00,-0.697

188,Median_Cells_Texture_InverseDifferenceMoment_Syto_5_00,-0.718

189,Median_Cells_Texture_SumAverage_Hoechst_10_00,1.059

190,Median_Cells_Texture_SumAverage_Hoechst_3_00,0.694

191,Median_Cells_Texture_SumAverage_Hoechst_5_00,1.03

192,Median_Cells_Texture_SumAverage_Mito_10_00,2.339

193,Median_Cells_Texture_SumAverage_Mito_3_00,2.408

194,Median_Cells_Texture_SumAverage_Mito_5_00,2.275

195,Median_Cells_Texture_SumAverage_Syto_10_00,0.423

196,Median_Cells_Texture_SumAverage_Syto_3_00,0.447

197,Median_Cells_Texture_SumAverage_Syto_5_00,0.45

198,Median_Cells_Texture_SumEntropy_Mito_10_00,2.879

199,Median_Cells_Texture_SumEntropy_Mito_3_00,2.562

200,Median_Cells_Texture_SumEntropy_Mito_5_00,2.762

201,Median_Cells_Texture_SumEntropy_Ph_golgi_10_00,1.709

202,Median_Cells_Texture_SumEntropy_Ph_golgi_3_00,3.312

203,Median_Cells_Texture_SumEntropy_Ph_golgi_5_00,2.575

204,Median_Cells_Texture_SumEntropy_Syto_10_00,0.261

205,Median_Cells_Texture_SumEntropy_Syto_3_00,0.347

206,Median_Cells_Texture_SumEntropy_Syto_5_00,0.22

207,Median_Cells_Texture_SumVariance_Hoechst_10_00,-0.534

208,Median_Cells_Texture_SumVariance_Hoechst_3_00,-0.212

209,Median_Cells_Texture_SumVariance_Hoechst_5_00,-0.499

210,Median_Cells_Texture_SumVariance_Mito_10_00,2.511

211,Median_Cells_Texture_SumVariance_Mito_3_00,2.235

212,Median_Cells_Texture_SumVariance_Mito_5_00,2.254

213,Median_Cells_Texture_SumVariance_Ph_golgi_10_00,0.39

214,Median_Cells_Texture_SumVariance_Ph_golgi_3_00,0.63

215,Median_Cells_Texture_SumVariance_Ph_golgi_5_00,0.613

216,Median_Cells_Texture_SumVariance_Syto_3_00,0.121

217,Median_Cells_Texture_SumVariance_Syto_5_00,0.111

218,Median_Cells_Texture_Variance_Hoechst_10_00,-0.3

219,Median_Cells_Texture_Variance_Hoechst_3_00,-0.244

220,Median_Cells_Texture_Variance_Hoechst_5_00,-0.252

221,Median_Cells_Texture_Variance_Mito_10_00,1.823

222,Median_Cells_Texture_Variance_Mito_3_00,2.106

223,Median_Cells_Texture_Variance_Mito_5_00,1.992

224,Median_Cells_Texture_Variance_Ph_golgi_10_00,0.506

225,Median_Cells_Texture_Variance_Ph_golgi_3_00,0.721

226,Median_Cells_Texture_Variance_Ph_golgi_5_00,0.657

227,Median_Cells_Texture_Variance_Syto_10_00,0.223

228,Median_Cells_Texture_Variance_Syto_3_00,0.221

229,Median_Cells_Texture_Variance_Syto_5_00,0.227

230,Median_Cytoplasm_AreaShape_Area,-2.646

231,Median_Cytoplasm_AreaShape_MinFeretDiameter,-4.165

232,Median_Cytoplasm_AreaShape_MinorAxisLength,-2.046

233,Median_Cytoplasm_AreaShape_Perimeter,-2.052

234,Median_Cytoplasm_Correlation_Correlation_ER_Ph_golgi,1.16

235,Median_Cytoplasm_Correlation_Correlation_ER_Syto,0.698

236,Median_Cytoplasm_Correlation_Correlation_Hoechst_ER,0.317

237,Median_Cytoplasm_Correlation_Correlation_Hoechst_Mito,-0.789

238,Median_Cytoplasm_Correlation_Correlation_Hoechst_Ph_golgi,-1.314

239,Median_Cytoplasm_Correlation_Correlation_Hoechst_Syto,-1.957

240,Median_Cytoplasm_Correlation_Correlation_Mito_ER,2.106

241,Median_Cytoplasm_Correlation_Correlation_Mito_Ph_golgi,0.648

242,Median_Cytoplasm_Correlation_Correlation_Mito_Syto,0.045

243,Median_Cytoplasm_Correlation_Correlation_Syto_Ph_golgi,0.962

244,Median_Cytoplasm_Correlation_K_ER_Syto,0.004

245,Median_Cytoplasm_Correlation_K_Hoechst_ER,1.421

246,Median_Cytoplasm_Correlation_K_Hoechst_Mito,1.333

247,Median_Cytoplasm_Correlation_K_Hoechst_Ph_golgi,3.029

248,Median_Cytoplasm_Correlation_K_Hoechst_Syto,0.306

249,Median_Cytoplasm_Correlation_K_Mito_Hoechst,-1.001

250,Median_Cytoplasm_Correlation_K_Mito_Syto,-0.358

251,Median_Cytoplasm_Correlation_K_Ph_golgi_Syto,-0.207

252,Median_Cytoplasm_Correlation_K_Syto_ER,0.116

253,Median_Cytoplasm_Correlation_K_Syto_Hoechst,-0.317

254,Median_Cytoplasm_Correlation_K_Syto_Mito,0.453

255,Median_Cytoplasm_Correlation_K_Syto_Ph_golgi,0.169

256,Median_Cytoplasm_Correlation_Manders_ER_Hoechst,0.0

257,Median_Cytoplasm_Correlation_Manders_ER_Syto,0.0

258,Median_Cytoplasm_Correlation_Manders_Hoechst_Syto,0.0

259,Median_Cytoplasm_Correlation_Manders_Mito_Hoechst,0.0

260,Median_Cytoplasm_Correlation_Manders_Mito_Syto,0.0

261,Median_Cytoplasm_Correlation_Manders_Ph_golgi_Hoechst,0.0

262,Median_Cytoplasm_Correlation_Manders_Ph_golgi_Syto,0.0

263,Median_Cytoplasm_Correlation_Manders_Syto_Hoechst,0.0

264,Median_Cytoplasm_Correlation_Overlap_ER_Syto,5.299

265,Median_Cytoplasm_Correlation_Overlap_Hoechst_ER,0.552

266,Median_Cytoplasm_Correlation_Overlap_Hoechst_Mito,-0.109

267,Median_Cytoplasm_Correlation_Overlap_Hoechst_Ph_golgi,-0.349

268,Median_Cytoplasm_Correlation_Overlap_Mito_Ph_golgi,-0.694

269,Median_Cytoplasm_Correlation_Overlap_Mito_Syto,1.756

270,Median_Cytoplasm_Correlation_Overlap_Syto_Ph_golgi,0.324

271,Median_Cytoplasm_Correlation_RWC_ER_Hoechst,-0.032

272,Median_Cytoplasm_Correlation_RWC_ER_Mito,0.635

273,Median_Cytoplasm_Correlation_RWC_Hoechst_Mito,-1.773

274,Median_Cytoplasm_Correlation_RWC_Hoechst_Ph_golgi,-3.036

275,Median_Cytoplasm_Correlation_RWC_Hoechst_Syto,-2.776

276,Median_Cytoplasm_Correlation_RWC_Mito_Hoechst,-2.379

277,Median_Cytoplasm_Correlation_RWC_Mito_Syto,-0.681

278,Median_Cytoplasm_Correlation_RWC_Ph_golgi_Hoechst,-2.128

279,Median_Cytoplasm_Correlation_RWC_Ph_golgi_Mito,-0.462

280,Median_Cytoplasm_Correlation_RWC_Ph_golgi_Syto,-0.564

281,Median_Cytoplasm_Correlation_RWC_Syto_Hoechst,-1.785

282,Median_Cytoplasm_Correlation_RWC_Syto_Mito,-0.355

283,Median_Cytoplasm_Granularity_1_Mito,-2.746

284,Median_Cytoplasm_Granularity_1_Ph_golgi,-2.481

285,Median_Cytoplasm_Granularity_1_Syto,-1.637

286,Median_Cytoplasm_Granularity_2_ER,-0.3

287,Median_Cytoplasm_Granularity_2_Mito,2.24

288,Median_Cytoplasm_Granularity_2_Ph_golgi,4.629

289,Median_Cytoplasm_Granularity_3_ER,1.358

290,Median_Cytoplasm_Granularity_3_Mito,5.323

291,Median_Cytoplasm_Granularity_3_Ph_golgi,3.186

292,Median_Cytoplasm_Granularity_3_Syto,2.217

293,Median_Cytoplasm_Granularity_4_Mito,1.465

294,Median_Cytoplasm_Granularity_4_Ph_golgi,0.994

295,Median_Cytoplasm_Granularity_5_Mito,1.42

296,Median_Cytoplasm_Granularity_5_Ph_golgi,1.781

297,Median_Cytoplasm_Intensity_IntegratedIntensity_Syto,-0.107

298,Median_Cytoplasm_Intensity_LowerQuartileIntensity_Mito,1.805

299,Median_Cytoplasm_Intensity_MADIntensity_ER,12.252

300,Median_Cytoplasm_Intensity_MADIntensity_Hoechst,0.0

301,Median_Cytoplasm_Intensity_MADIntensity_Mito,4.548

302,Median_Cytoplasm_Intensity_MADIntensity_Ph_golgi,7.444

303,Median_Cytoplasm_Intensity_MADIntensity_Syto,0.688

304,Median_Cytoplasm_Intensity_MaxIntensityEdge_Hoechst,-0.677

305,Median_Cytoplasm_Intensity_MaxIntensityEdge_Mito,2.807

306,Median_Cytoplasm_Intensity_MaxIntensityEdge_Ph_golgi,0.684

307,Median_Cytoplasm_Intensity_MaxIntensityEdge_Syto,0.317

308,Median_Cytoplasm_Intensity_MaxIntensity_Hoechst,-0.677

309,Median_Cytoplasm_Intensity_MaxIntensity_Mito,2.361

310,Median_Cytoplasm_Intensity_MaxIntensity_Ph_golgi,0.293

311,Median_Cytoplasm_Intensity_MaxIntensity_Syto,0.38

312,Median_Cytoplasm_Intensity_MeanIntensityEdge_Hoechst,1.537

313,Median_Cytoplasm_Intensity_MeanIntensity_Mito,2.474

314,Median_Cytoplasm_Intensity_MeanIntensity_Syto,0.546

315,Median_Cytoplasm_Intensity_MedianIntensity_ER,3.855

316,Median_Cytoplasm_Intensity_MedianIntensity_Mito,2.387

317,Median_Cytoplasm_Intensity_MedianIntensity_Syto,0.579

318,Median_Cytoplasm_Intensity_MinIntensityEdge_Mito,0.922

319,Median_Cytoplasm_Intensity_MinIntensityEdge_Syto,0.68

320,Median_Cytoplasm_Intensity_MinIntensity_Mito,0.996

321,Median_Cytoplasm_Intensity_MinIntensity_Syto,0.706

322,Median_Cytoplasm_Intensity_StdIntensityEdge_Hoechst,0.639

323,Median_Cytoplasm_Intensity_StdIntensityEdge_Mito,2.079

324,Median_Cytoplasm_Intensity_StdIntensityEdge_Ph_golgi,0.86

325,Median_Cytoplasm_Intensity_StdIntensityEdge_Syto,0.186

326,Median_Cytoplasm_Intensity_StdIntensity_Hoechst,1.255

327,Median_Cytoplasm_Intensity_StdIntensity_Mito,2.381

328,Median_Cytoplasm_Intensity_StdIntensity_Ph_golgi,2.13

329,Median_Cytoplasm_Intensity_StdIntensity_Syto,0.411

330,Median_Cytoplasm_Intensity_UpperQuartileIntensity_ER,7.88

331,Median_Cytoplasm_Intensity_UpperQuartileIntensity_Mito,3.249

332,Median_Cytoplasm_Intensity_UpperQuartileIntensity_Syto,0.549

333,Median_Cytoplasm_RadialDistribution_MeanFrac_ER_1of4,5.126

334,Median_Cytoplasm_RadialDistribution_MeanFrac_ER_2of4,5.161

335,Median_Cytoplasm_RadialDistribution_MeanFrac_Mito_1of4,2.989

336,Median_Cytoplasm_RadialDistribution_MeanFrac_Mito_2of4,3.885

337,Median_Cytoplasm_RadialDistribution_MeanFrac_Mito_3of4,3.721

338,Median_Cytoplasm_RadialDistribution_MeanFrac_Mito_4of4,-5.355

339,Median_Cytoplasm_RadialDistribution_MeanFrac_Ph_golgi_1of4,2.197

340,Median_Cytoplasm_RadialDistribution_MeanFrac_Ph_golgi_2of4,2.443

341,Median_Cytoplasm_RadialDistribution_MeanFrac_Ph_golgi_3of4,4.079

342,Median_Cytoplasm_RadialDistribution_MeanFrac_Ph_golgi_4of4,-4.17

343,Median_Cytoplasm_RadialDistribution_RadialCV_Mito_1of4,3.84

344,Median_Cytoplasm_RadialDistribution_RadialCV_Mito_2of4,3.163

345,Median_Cytoplasm_RadialDistribution_RadialCV_Mito_3of4,1.714

346,Median_Cytoplasm_RadialDistribution_RadialCV_Mito_4of4,2.317

347,Median_Cytoplasm_RadialDistribution_RadialCV_Ph_golgi_1of4,5.611

348,Median_Cytoplasm_RadialDistribution_RadialCV_Ph_golgi_2of4,11.8

349,Median_Cytoplasm_RadialDistribution_RadialCV_Ph_golgi_3of4,6.736

350,Median_Cytoplasm_Texture_AngularSecondMoment_ER_3_00,-7.376

351,Median_Cytoplasm_Texture_AngularSecondMoment_ER_5_00,-8.346

352,Median_Cytoplasm_Texture_AngularSecondMoment_Mito_10_00,-3.258

353,Median_Cytoplasm_Texture_AngularSecondMoment_Mito_3_00,-3.268

354,Median_Cytoplasm_Texture_AngularSecondMoment_Mito_5_00,-3.121

355,Median_Cytoplasm_Texture_AngularSecondMoment_Ph_golgi_10_00,-8.994

356,Median_Cytoplasm_Texture_AngularSecondMoment_Ph_golgi_3_00,-7.887

357,Median_Cytoplasm_Texture_AngularSecondMoment_Ph_golgi_5_00,-8.939

358,Median_Cytoplasm_Texture_AngularSecondMoment_Syto_10_00,-0.692

359,Median_Cytoplasm_Texture_AngularSecondMoment_Syto_3_00,-0.681

360,Median_Cytoplasm_Texture_AngularSecondMoment_Syto_5_00,-0.697

361,Median_Cytoplasm_Texture_Contrast_ER_10_00,-1.114

362,Median_Cytoplasm_Texture_Contrast_ER_3_00,-0.945

363,Median_Cytoplasm_Texture_Contrast_ER_5_00,-0.858

364,Median_Cytoplasm_Texture_Contrast_Hoechst_10_00,2.3

365,Median_Cytoplasm_Texture_Contrast_Hoechst_3_00,-0.284

366,Median_Cytoplasm_Texture_Contrast_Hoechst_5_00,0.553

367,Median_Cytoplasm_Texture_Contrast_Mito_10_00,1.453

368,Median_Cytoplasm_Texture_Contrast_Mito_3_00,0.6

369,Median_Cytoplasm_Texture_Contrast_Mito_5_00,0.783

370,Median_Cytoplasm_Texture_Contrast_Ph_golgi_10_00,2.785

371,Median_Cytoplasm_Texture_Contrast_Ph_golgi_3_00,0.802

372,Median_Cytoplasm_Texture_Contrast_Ph_golgi_5_00,1.475

373,Median_Cytoplasm_Texture_Contrast_Syto_10_00,0.67

374,Median_Cytoplasm_Texture_Contrast_Syto_5_00,0.761

375,Median_Cytoplasm_Texture_Correlation_Ph_golgi_10_00,-0.454

376,Median_Cytoplasm_Texture_Correlation_Ph_golgi_3_00,0.617

377,Median_Cytoplasm_Texture_Correlation_Ph_golgi_5_00,0.406

378,Median_Cytoplasm_Texture_DifferenceEntropy_ER_3_00,0.337

379,Median_Cytoplasm_Texture_DifferenceEntropy_ER_5_00,0.828

380,Median_Cytoplasm_Texture_DifferenceEntropy_Mito_10_00,1.497

381,Median_Cytoplasm_Texture_DifferenceEntropy_Mito_3_00,0.56

382,Median_Cytoplasm_Texture_DifferenceEntropy_Mito_5_00,0.777

383,Median_Cytoplasm_Texture_DifferenceEntropy_Ph_golgi_10_00,4.097

384,Median_Cytoplasm_Texture_DifferenceEntropy_Ph_golgi_3_00,1.316

385,Median_Cytoplasm_Texture_DifferenceEntropy_Ph_golgi_5_00,2.213

386,Median_Cytoplasm_Texture_DifferenceEntropy_Syto_10_00,0.617

387,Median_Cytoplasm_Texture_DifferenceEntropy_Syto_3_00,0.804

388,Median_Cytoplasm_Texture_DifferenceEntropy_Syto_5_00,0.833

389,Median_Cytoplasm_Texture_DifferenceVariance_Mito_10_00,-2.398

390,Median_Cytoplasm_Texture_DifferenceVariance_Mito_3_00,-2.163

391,Median_Cytoplasm_Texture_DifferenceVariance_Mito_5_00,-2.4

392,Median_Cytoplasm_Texture_DifferenceVariance_Ph_golgi_10_00,-2.055

393,Median_Cytoplasm_Texture_DifferenceVariance_Ph_golgi_3_00,-0.999

394,Median_Cytoplasm_Texture_DifferenceVariance_Ph_golgi_5_00,-1.455

395,Median_Cytoplasm_Texture_DifferenceVariance_Syto_10_00,-0.68

396,Median_Cytoplasm_Texture_DifferenceVariance_Syto_3_00,-0.57

397,Median_Cytoplasm_Texture_DifferenceVariance_Syto_5_00,-0.684

398,Median_Cytoplasm_Texture_Entropy_ER_3_00,4.763

399,Median_Cytoplasm_Texture_Entropy_ER_5_00,5.154

400,Median_Cytoplasm_Texture_Entropy_Mito_10_00,2.846

401,Median_Cytoplasm_Texture_Entropy_Mito_3_00,2.315

402,Median_Cytoplasm_Texture_Entropy_Mito_5_00,2.273

403,Median_Cytoplasm_Texture_Entropy_Ph_golgi_10_00,8.399

404,Median_Cytoplasm_Texture_Entropy_Ph_golgi_3_00,4.129

405,Median_Cytoplasm_Texture_Entropy_Ph_golgi_5_00,5.828

406,Median_Cytoplasm_Texture_Entropy_Syto_10_00,0.689

407,Median_Cytoplasm_Texture_Entropy_Syto_3_00,0.73

408,Median_Cytoplasm_Texture_Entropy_Syto_5_00,0.738

409,Median_Cytoplasm_Texture_InfoMeas2_Syto_10_00,-0.317

410,Median_Cytoplasm_Texture_InverseDifferenceMoment_ER_10_00,-8.084

411,Median_Cytoplasm_Texture_InverseDifferenceMoment_ER_3_00,-3.199

412,Median_Cytoplasm_Texture_InverseDifferenceMoment_ER_5_00,-6.771

413,Median_Cytoplasm_Texture_InverseDifferenceMoment_Mito_10_00,-2.487

414,Median_Cytoplasm_Texture_InverseDifferenceMoment_Mito_3_00,-0.891

415,Median_Cytoplasm_Texture_InverseDifferenceMoment_Mito_5_00,-1.298

416,Median_Cytoplasm_Texture_InverseDifferenceMoment_Ph_golgi_10_00,-11.202

417,Median_Cytoplasm_Texture_InverseDifferenceMoment_Ph_golgi_3_00,-2.728

418,Median_Cytoplasm_Texture_InverseDifferenceMoment_Ph_golgi_5_00,-4.843

419,Median_Cytoplasm_Texture_InverseDifferenceMoment_Syto_10_00,-1.013

420,Median_Cytoplasm_Texture_InverseDifferenceMoment_Syto_3_00,-0.9

421,Median_Cytoplasm_Texture_InverseDifferenceMoment_Syto_5_00,-0.952

422,Median_Cytoplasm_Texture_SumAverage_ER_10_00,4.104

423,Median_Cytoplasm_Texture_SumAverage_Mito_10_00,2.748

424,Median_Cytoplasm_Texture_SumAverage_Mito_3_00,2.489

425,Median_Cytoplasm_Texture_SumAverage_Mito_5_00,2.653

426,Median_Cytoplasm_Texture_SumAverage_Syto_10_00,0.47

427,Median_Cytoplasm_Texture_SumAverage_Syto_3_00,0.488

428,Median_Cytoplasm_Texture_SumAverage_Syto_5_00,0.449

429,Median_Cytoplasm_Texture_SumEntropy_Mito_10_00,4.31

430,Median_Cytoplasm_Texture_SumEntropy_Mito_3_00,3.782

431,Median_Cytoplasm_Texture_SumEntropy_Mito_5_00,3.748

432,Median_Cytoplasm_Texture_SumEntropy_Ph_golgi_10_00,6.323

433,Median_Cytoplasm_Texture_SumEntropy_Ph_golgi_3_00,7.598

434,Median_Cytoplasm_Texture_SumEntropy_Ph_golgi_5_00,7.934

435,Median_Cytoplasm_Texture_SumEntropy_Syto_10_00,0.399

436,Median_Cytoplasm_Texture_SumEntropy_Syto_3_00,0.501

437,Median_Cytoplasm_Texture_SumEntropy_Syto_5_00,0.487

438,Median_Cytoplasm_Texture_SumVariance_Hoechst_10_00,1.097

439,Median_Cytoplasm_Texture_SumVariance_Hoechst_3_00,0.411

440,Median_Cytoplasm_Texture_SumVariance_Hoechst_5_00,0.705

441,Median_Cytoplasm_Texture_SumVariance_Mito_10_00,3.704

442,Median_Cytoplasm_Texture_SumVariance_Mito_3_00,2.977

443,Median_Cytoplasm_Texture_SumVariance_Mito_5_00,3.288

444,Median_Cytoplasm_Texture_SumVariance_Ph_golgi_10_00,2.879

445,Median_Cytoplasm_Texture_SumVariance_Ph_golgi_3_00,2.748

446,Median_Cytoplasm_Texture_SumVariance_Ph_golgi_5_00,3.118

447,Median_Cytoplasm_Texture_SumVariance_Syto_10_00,0.333

448,Median_Cytoplasm_Texture_SumVariance_Syto_3_00,0.43

449,Median_Cytoplasm_Texture_SumVariance_Syto_5_00,0.434

450,Median_Cytoplasm_Texture_Variance_Hoechst_10_00,1.149

451,Median_Cytoplasm_Texture_Variance_Hoechst_3_00,1.075

452,Median_Cytoplasm_Texture_Variance_Hoechst_5_00,1.27

453,Median_Cytoplasm_Texture_Variance_Mito_10_00,2.579

454,Median_Cytoplasm_Texture_Variance_Mito_3_00,2.846

455,Median_Cytoplasm_Texture_Variance_Mito_5_00,2.866

456,Median_Cytoplasm_Texture_Variance_Ph_golgi_10_00,2.792

457,Median_Cytoplasm_Texture_Variance_Ph_golgi_3_00,2.573

458,Median_Cytoplasm_Texture_Variance_Ph_golgi_5_00,2.644

459,Median_Cytoplasm_Texture_Variance_Syto_10_00,0.513

460,Median_Cytoplasm_Texture_Variance_Syto_3_00,0.53

461,Median_Cytoplasm_Texture_Variance_Syto_5_00,0.541

462,Median_Nuclei_AreaShape_Area,-7.118

463,Median_Nuclei_AreaShape_MajorAxisLength,-6.726

464,Median_Nuclei_AreaShape_MaxFeretDiameter,-9.46

465,Median_Nuclei_AreaShape_Perimeter,-10.499

466,Median_Nuclei_AreaShape_Solidity,0.896

467,Median_Nuclei_Correlation_Correlation_Hoechst_Syto,0.196

468,Median_Nuclei_Correlation_Correlation_Mito_Ph_golgi,-0.674

469,Median_Nuclei_Correlation_Correlation_Mito_Syto,-1.04

470,Median_Nuclei_Correlation_K_Mito_Ph_golgi,-0.842

471,Median_Nuclei_Correlation_K_Ph_golgi_Mito,0.922

472,Median_Nuclei_Correlation_K_Ph_golgi_Syto,-0.119

473,Median_Nuclei_Correlation_K_Syto_Ph_golgi,0.08

474,Median_Nuclei_Correlation_Overlap_Hoechst_Mito,0.37

475,Median_Nuclei_Correlation_Overlap_Hoechst_Ph_golgi,0.0

476,Median_Nuclei_Correlation_Overlap_Hoechst_Syto,0.562

477,Median_Nuclei_Correlation_RWC_Mito_Syto,1.17

478,Median_Nuclei_Correlation_RWC_Syto_Mito,0.57

479,Median_Nuclei_Granularity_1_Mito,-1.839

480,Median_Nuclei_Granularity_2_Hoechst,2.869

481,Median_Nuclei_Granularity_3_Mito,0.208

482,Median_Nuclei_Granularity_3_Ph_golgi,-1.368

483,Median_Nuclei_Granularity_4_Mito,-0.25

484,Median_Nuclei_Intensity_LowerQuartileIntensity_Hoechst,0.893

485,Median_Nuclei_Intensity_MADIntensity_Hoechst,-1.186

486,Median_Nuclei_Intensity_MADIntensity_Mito,0.953

487,Median_Nuclei_Intensity_MaxIntensityEdge_Hoechst,0.374

488,Median_Nuclei_Intensity_MaxIntensityEdge_Mito,2.677

489,Median_Nuclei_Intensity_MaxIntensityEdge_Ph_golgi,0.61

490,Median_Nuclei_Intensity_MaxIntensityEdge_Syto,0.19

491,Median_Nuclei_Intensity_MaxIntensity_Hoechst,-0.21

492,Median_Nuclei_Intensity_MaxIntensity_Mito,2.628

493,Median_Nuclei_Intensity_MaxIntensity_Ph_golgi,0.165

494,Median_Nuclei_Intensity_MeanIntensityEdge_Hoechst,1.353

495,Median_Nuclei_Intensity_MeanIntensity_Hoechst,0.651

496,Median_Nuclei_Intensity_MedianIntensity_Hoechst,0.861

497,Median_Nuclei_Intensity_MinIntensityEdge_Hoechst,1.136

498,Median_Nuclei_Intensity_MinIntensity_Hoechst,1.069

499,Median_Nuclei_Intensity_StdIntensityEdge_Mito,0.898

500,Median_Nuclei_Intensity_StdIntensityEdge_Ph_golgi,0.0

501,Median_Nuclei_Intensity_StdIntensityEdge_Syto,-0.565

502,Median_Nuclei_Intensity_StdIntensity_Mito,0.871

503,Median_Nuclei_Intensity_StdIntensity_Ph_golgi,-0.792

504,Median_Nuclei_Intensity_UpperQuartileIntensity_Hoechst,0.841

505,Median_Nuclei_RadialDistribution_FracAtD_Ph_golgi_3of4,-0.597

506,Median_Nuclei_RadialDistribution_MeanFrac_Mito_1of4,-3.309

507,Median_Nuclei_RadialDistribution_MeanFrac_Mito_2of4,-3.435

508,Median_Nuclei_RadialDistribution_MeanFrac_Mito_3of4,-1.188

509,Median_Nuclei_RadialDistribution_MeanFrac_Mito_4of4,2.658

510,Median_Nuclei_RadialDistribution_MeanFrac_Ph_golgi_2of4,-2.212

511,Median_Nuclei_RadialDistribution_MeanFrac_Ph_golgi_3of4,-1.744

512,Median_Nuclei_RadialDistribution_MeanFrac_Ph_golgi_4of4,1.839

513,Median_Nuclei_Texture_AngularSecondMoment_Hoechst_10_00,1.402

514,Median_Nuclei_Texture_AngularSecondMoment_Hoechst_3_00,1.222

515,Median_Nuclei_Texture_AngularSecondMoment_Hoechst_5_00,1.614

516,Median_Nuclei_Texture_AngularSecondMoment_Mito_3_00,-0.774

517,Median_Nuclei_Texture_AngularSecondMoment_Mito_5_00,-0.675

518,Median_Nuclei_Texture_Contrast_Hoechst_3_00,0.091

519,Median_Nuclei_Texture_Contrast_Hoechst_5_00,0.019

520,Median_Nuclei_Texture_Contrast_Mito_10_00,0.888

521,Median_Nuclei_Texture_Contrast_Mito_3_00,0.999

522,Median_Nuclei_Texture_Contrast_Mito_5_00,1.041

523,Median_Nuclei_Texture_Contrast_Ph_golgi_10_00,-0.79

524,Median_Nuclei_Texture_Contrast_Ph_golgi_3_00,-0.291

525,Median_Nuclei_Texture_Contrast_Ph_golgi_5_00,-0.239

526,Median_Nuclei_Texture_Correlation_Ph_golgi_3_00,-0.088

527,Median_Nuclei_Texture_DifferenceEntropy_Hoechst_10_00,-0.527

528,Median_Nuclei_Texture_DifferenceEntropy_Hoechst_3_00,-0.174

529,Median_Nuclei_Texture_DifferenceEntropy_Hoechst_5_00,0.247

530,Median_Nuclei_Texture_DifferenceEntropy_Mito_10_00,0.555

531,Median_Nuclei_Texture_DifferenceEntropy_Mito_3_00,0.99

532,Median_Nuclei_Texture_DifferenceEntropy_Mito_5_00,1.266

533,Median_Nuclei_Texture_DifferenceEntropy_Ph_golgi_3_00,-0.36

534,Median_Nuclei_Texture_DifferenceEntropy_Ph_golgi_5_00,-0.066

535,Median_Nuclei_Texture_DifferenceVariance_Hoechst_10_00,0.0

536,Median_Nuclei_Texture_DifferenceVariance_Hoechst_3_00,0.54

537,Median_Nuclei_Texture_DifferenceVariance_Hoechst_5_00,0.382

538,Median_Nuclei_Texture_DifferenceVariance_Mito_3_00,-1.399

539,Median_Nuclei_Texture_DifferenceVariance_Mito_5_00,-1.289

540,Median_Nuclei_Texture_DifferenceVariance_Ph_golgi_10_00,0.434

541,Median_Nuclei_Texture_DifferenceVariance_Ph_golgi_3_00,0.241

542,Median_Nuclei_Texture_DifferenceVariance_Ph_golgi_5_00,0.248

543,Median_Nuclei_Texture_Entropy_Hoechst_3_00,-0.681

544,Median_Nuclei_Texture_Entropy_Hoechst_5_00,-1.186

545,Median_Nuclei_Texture_Entropy_Mito_10_00,0.829

546,Median_Nuclei_Texture_Entropy_Mito_3_00,1.025

547,Median_Nuclei_Texture_Entropy_Mito_5_00,1.013

548,Median_Nuclei_Texture_InfoMeas2_Hoechst_10_00,0.509

549,Median_Nuclei_Texture_InfoMeas2_Mito_10_00,1.698

550,Median_Nuclei_Texture_InfoMeas2_Ph_golgi_10_00,-0.786

551,Median_Nuclei_Texture_InfoMeas2_Ph_golgi_3_00,-0.476

552,Median_Nuclei_Texture_InfoMeas2_Ph_golgi_5_00,-0.339

553,Median_Nuclei_Texture_InverseDifferenceMoment_Hoechst_10_00,0.696

554,Median_Nuclei_Texture_InverseDifferenceMoment_Hoechst_3_00,0.36

555,Median_Nuclei_Texture_InverseDifferenceMoment_Hoechst_5_00,0.561

556,Median_Nuclei_Texture_InverseDifferenceMoment_Mito_10_00,-2.297

557,Median_Nuclei_Texture_InverseDifferenceMoment_Mito_3_00,-0.479

558,Median_Nuclei_Texture_InverseDifferenceMoment_Mito_5_00,-0.392

559,Median_Nuclei_Texture_SumAverage_Hoechst_10_00,-0.299

560,Median_Nuclei_Texture_SumAverage_Hoechst_3_00,1.006

561,Median_Nuclei_Texture_SumAverage_Hoechst_5_00,0.807

562,Median_Nuclei_Texture_SumEntropy_Mito_10_00,0.491

563,Median_Nuclei_Texture_SumEntropy_Mito_3_00,0.967

564,Median_Nuclei_Texture_SumEntropy_Mito_5_00,0.653

565,Median_Nuclei_Texture_SumEntropy_Ph_golgi_10_00,-1.394

566,Median_Nuclei_Texture_SumEntropy_Ph_golgi_3_00,-1.331

567,Median_Nuclei_Texture_SumEntropy_Ph_golgi_5_00,-1.62

568,Median_Nuclei_Texture_SumVariance_Mito_10_00,0.459

569,Median_Nuclei_Texture_SumVariance_Mito_3_00,0.908

570,Median_Nuclei_Texture_SumVariance_Mito_5_00,0.833

571,Median_Nuclei_Texture_SumVariance_Ph_golgi_10_00,-1.011

572,Median_Nuclei_Texture_SumVariance_Ph_golgi_3_00,-1.287

573,Median_Nuclei_Texture_SumVariance_Ph_golgi_5_00,-1.421

574,Median_Nuclei_Texture_Variance_Mito_10_00,0.769

575,Median_Nuclei_Texture_Variance_Mito_3_00,0.93

576,Median_Nuclei_Texture_Variance_Mito_5_00,0.971

577,Median_Nuclei_Texture_Variance_Ph_golgi_10_00,-0.784

578,Median_Nuclei_Texture_Variance_Ph_golgi_3_00,-0.958

579,Median_Nuclei_Texture_Variance_Ph_golgi_5_00,-0.892

Table S5. Sequence information of primers used for gene expression analysis via RT-qPCR

Primer Name	Sequence (5’ to 3’)
GAPDH FW	GCA CCG TCA AGG CTG AGA AC
GAPDH RV	TGG TGA AGA CGC CAG TGG A
β-Actin FW	CCA ACC GCG AGA AGA TGA
β-Actin RV	CCA GAG GCG TAC AGG GAT AG
NRAS FW	CCT ATA CAA TGT ATG TAA TTT GTT TCC
NRAS RV	CAA TGC ACC AAA GTT TTA CAA TAT TTG AAC
G4-NRAS FW	AAC GTC CCG TGT GGG AGG GG
G4-NRAS RV	AGA CCC CGG AAC CGC CAT GA

General Chemical Information

The commercially available reagents were used without any further purification, unless otherwise mentioned. Solvents used for silica gel column chromatography were in laboratory grade. Dry solvents were purchased from Acros, Fischer Scientific and/or VWR, and used without further treatment. Oxygen and/or moisture sensitive solutions were transferred under inert gas atmosphere using cannulas and syringes. Analytical thin-layer chromatography (TLC) was performed on silica coated aluminum plates (Merck 60 F254) and visualization of products proceeded with UV irradiation (254 nm and/or 356 nm) or through potassium permanganate stain (1.5 g $KMnO_4$, 10 g K_2CO_3 in 1.25 mL of 10% aq. NaOH and 200 mL water). Analytical UHPLC-MS was performed on an Agilent 1260 II Infinity system equipped with a mass detector (UHPLC column: Zorbax Eclipse C18 Rapid Resolution 2.1x1x50 mm 1.8µm; LC-MS column: InfinityLab Poroshell 120 EC-C18, 2.1x150, 2.7 µm). Appropriate gradient systems were applied by water (+ 0.1% TFA) and mixing Acetonitrile (+ 0.1% TFA). The purification of crude products was performed by silica gel column chromatography (Merck 60, particle size 0.040-0.063 mm) using indicated solvents. ^{1}H NMR and ^{13}C NMR spectra were recorded with either a Bruker AV 400 Avance III HD (NanoBay), Agilent Technologies DD2, Bruker AV 500 Avance III HD (Prodigy), Bruker Avance NEO – 500 MHz, Bruker AV 600 Avance III HD (CryoProbe) or a Bruker AV 700 Avance III HD (CryoProbe) spectrometers. Data is reported in parts per million (ppm) with reference to the used deuterated solvent ($CDCl_3$: 7.26 ppm, 77.16 ppm; DMSO-*d*$_6$: 2.50 ppm, 39.52 ppm). Chemical shift values are reported in ppm, multiplicity (s = singlet, d = doublet, t = triplet, dd = double doublet, m = multiplet, br = broad signal, q = quintet, dt = double of triplets, td = triplet of doubles), integration values, and coupling constant values in Hz. Signals were assigned to their corresponding hydrogens or carbons based on 2D NMR correlations (^{1}H/^{1}H COSY, ^{1}H/^{1}H NOESY, ^{1}H/^{13}C HSQC, ^{1}H/^{13}C HMBC). High-resolution mass spectrometry (HRMS) was performed on an LTQ Orbitrap mass spectrometer coupled to an Accela HPLC-System (HPLC column: Hypersyl GOLD, 50 mm x 1 mm, particle size 1.9 µm, ionization method: electron spray ionization, ESI).

Synthetic Procedures and Compound Characterization

The purification using preparative HPLC was performed in a BUCHI C-850 prep-flash system equipped with VP 125/21 NUCIEODUR C18 Gravity 5 µm column. Absorbance was monitored at 210nm, 254nm, 280nm, and 345 nm. A linear gradient with a flowrate of 20 mL/min from 0-100% Acetonitrile in water with 0.1% (v/v) TFA over 120 min was used for small molecule purification. Purity was assessed by analytical HPLC using a Zorbax Eclipse C18 Rapid Resolution 2.1 x 1 x 50 mm 1.8µm with a flow rate of 1 mL/min and a linear gradient from 0-100% acetonitrile in water with 0.1% (v/v) TFA over 6 min.

Synthesis of the RNase L binder MD4

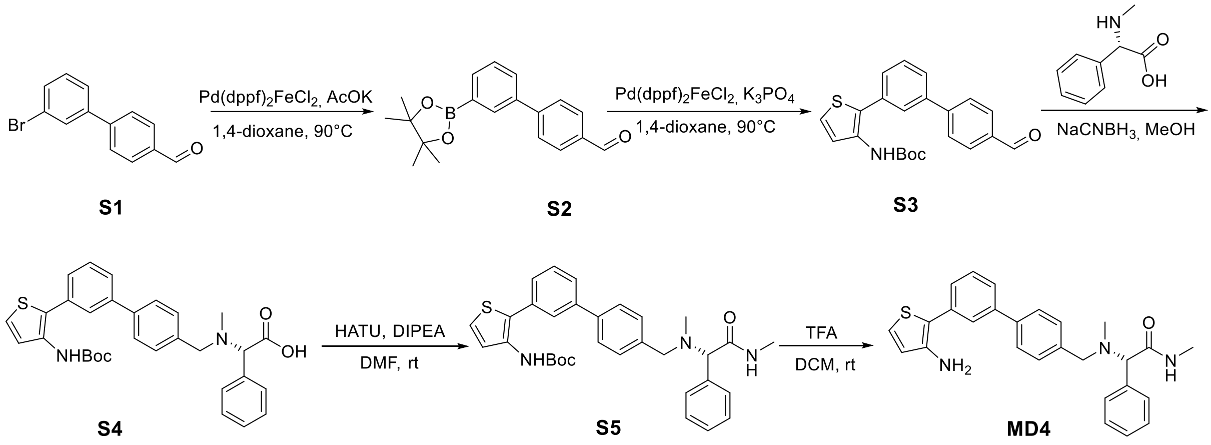

3'-(4,4,5,5-Tetramethyl-1,3,2-dioxaborolan-2-yl)-[1,1'-biphenyl]-4-carbaldehyde **(S2)**

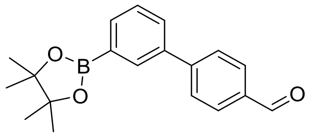

To a stirred solution of 3’-Bromo-[1,1’-biphenyl]-4-carbaldehyde (**S1**, 2.0 g, 7.66 mmol) and bis(pinacolato)diboron (2.92 g, 11.49 mmol) in anhydrous 1,4-dioxane (30 mL) was added potassium acetate (1.13 g, 11.49 mmol). Nitrogen gas was bubbled through the reaction mixture for 2 min before the addition of 1,1'-[bis(diphenylphosphino) ferrocene] dichloropalladium (II) dichloride (0.85 g, 1.53 mmol). The reaction mixture was then stirred at 90 °C overnight. Upon completion of the reaction monitored by LC-MS, the solvent was evaporated and the crude product was purified using column chromatography (PE: EA, 40:1) to afford compound **S2** (2.32 g, 7.46 mmol, 97.4% yield) as a white solid.

LC-MS (ESI+) *m/z* calculated for $[C_{19}H_{22}BO_3]^+$: 309.2; found, 309.4.

^{1}H NMR (600 MHz, DMSO-d_6) δ 10.06 (s, 1H), 8.00 (d, *J* = 8.3 Hz, 2H), 7.92 (d, *J* = 8.2 Hz, 2H), 7.79 (q, *J* = 8.2 Hz, 4H), 1.31 (s, 12H). **^{13}C NMR** (151 MHz, DMSO-d_6) δ 192.70, 145.40, 141.49, 135.36, 135.15, 130.16, 127.46, 126.54, 83.78, 82.81, 24.66.

tert-Butyl (2-(4'-formyl-[1,1'-biphenyl]-3-yl) thiophen-3-yl) carbamate **(S3)**

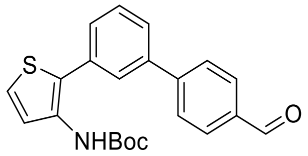

To a stirred solution of 3'-(4,4,5,5-tetramethyl-1,3,2-dioxaborolan-2-yl)-[1,1'-biphenyl]-4-carbaldehyde (**S2**, 500 mg, 1.62 mmol) in anhydrous 1,4-dioxane (10 mL) were *tert*-butyl (2-bromothiophen-3-yl) carbamate (451.3 mg, 1.62 mmol) and tripotassium phosphate (1.0 g, 4.87 mmol). Nitrogon gas was bubbled through the reaction mixture for 3 min, followed by the addition of 1,1'-[bis(diphenylphosphino) ferrocene] dichloropalladium (II) dichloride (90.0 mg, 0.16 mmol). The reaction mixture was then stirred at 90 °C overnight. Upon completion of the reaction monitored by LC-MS, the solvent was evaporated and the crude product was purified using column chromatography (PE: EA, 40:1) to afford compound **S3** (0.43 g, 1.13 mmol, 69.8% yield) as a yellow oil.

LC-MS (ESI+) *m/z* calculated for $[C_{22}H_{22}NO_3S]^+$: 380.1; found, 380.3.

^{1}H NMR (700 MHz, Chloroform-*d*) δ 10.10 – 10.06 (m, 1H), 8.00 – 7.96 (m, 2H), 7.80 – 7.77 (m, 2H), 7.72 (t, *J* = 1.8 Hz, 1H), 7.64 – 7.61 (m, 1H), 7.57 (t, *J* = 7.7 Hz, 1H), 7.52 (dt, *J* = 7.6, 1.4 Hz, 1H), 7.28 (d, *J* = 5.5 Hz, 1H), 1.49 (s, 9H). **^{13}C NMR** (176 MHz, $CDCl_3$) δ 191.99, 153.09, 146.59, 140.95, 135.65, 133.90, 132.40, 130.50, 130.09, 128.74, 127.99, 127.93, 126.87, 123.97, 67.24, 60.55, 28.46.

(*S*)-2-(((3'-(3-((tert-Butoxycarbonyl) amino) thiophen-2-yl)-[1,1'-biphenyl]-4-yl) methyl) (methyl)amino)-2-phenylacetic acid **(S4)**

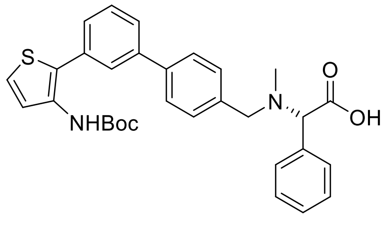

To a stirred solution of tert-butyl (2-(4'-formyl-[1,1'-biphenyl]-3-yl) thiophen-3-yl) carbamate (**S3**, 1.0 g, 2.64 mmol) in MeOH (10 mL) were added (S)-2-(methylamino)-2-phenylacetic acid (0.44 g, 2.64 mmol) and one drop of acetate acid. The reaction mixture was stirred at room temperature for 30 min, followed by the addition of sodium cyanoborohydride (0.25 g, 3.95mmol) portionwise. The reaction was stirred at room temperature for extra 3 hours. Upon completion of the reaction monitored by LC-MS, a portion of H_2O was added to quench the excess reducing reagent. The solvent was then evaporated and the crude product was purified by silica gel column chromatography (DCM: MeOH, 20:1) to obtain the title product **S4** (0.96 g, 1.82 mmol, 68.9% yield) as a white solid.

LC-MS (ESI+) *m/z* calculated for $[C_{31}H_{33}N_2O_4S]^+$: 529.2; found, 529.0.

tert-Butyl (*S*)-(2-(4'-((methyl(2-(methylamino)-2-oxo-1-phenylethyl) amino) methyl)-[1,1'-biphenyl]-3-yl) thiophen-3-yl) carbamate **(S5)**

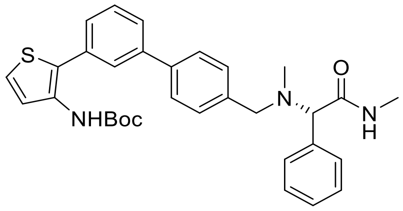

To a solution of 1-[bis(dimethylamino) methylene]-1H-1,2,3-triazolo[4,5-b] pyridinium 3-oxide hexafluorophosphate (HATU, 539.4 mg, 1.42 mmol), *N, N*-diisopropylethylamine (DIPEA, 366.7 mg, 2.84 mmol) in anhydrous DMF (5 mL) was added **S4** (500 mg, 0.95 mmol). The mixture was stirred at room temperature for 15 min, followed by the addition of N-methylamine in 2 M THF solution (945 µL, 1.89 mmol). The reaction mixture was stirred at room temperature for 6 hours. Upon completion of the reaction monitored by LC-MS, the reaction mixture was diluted with water (30 mL) and extracted with ethyl acetate (3 × 20 mL). The combined organic phases were dried with $MgSO_4$ and concentrated under the vacuum. The residue was purified using silica gel with DCM: MeOH (100:1-20:1) to afford the **S5** (324 mg, 0.6 mmol, 63.2% yield) as a colorless solid.

LC-MS (ESI+) *m/z* calculated for $[C_{32}H_{36}N_2O_4S]^+$: 542.2; found, 542.3.

^{1}H NMR (500 MHz, DMSO-d_6) δ 7.66 (t, *J* = 1.8 Hz, 1H), 7.60 – 7.55 (m, 3H), 7.53 (t, *J* = 7.6 Hz, 1H), 7.44 (dt, *J* = 7.5, 1.5 Hz, 1H), 7.41 – 7.36 (m, 6H), 7.34 – 7.29 (m, 1H), 7.25 (m, 1H), 7.17 (q, *J* = 5.0 Hz, 1H), 4.12 (s, 1H), 3.62 (d, *J* = 13.5 Hz, 1H),

3.44 (d, J = 13.5 Hz, 1H), 2.89 (d, J = 5.0 Hz, 3H), 2.15 (s, 3H), 1.49 (s, 9H). **^{13}C NMR** (126 MHz, $CDCl_3$) δ 172.40, 162.67, 153.09, 142.02, 139.70, 138.18, 135.94, 133.55, 132.26, 129.84, 129.32, 129.29, 128.65, 128.25, 127.75, 127.60, 127.42, 126.59, 123.72, 80.87, 74.88, 59.42, 40.15, 28.45, 26.26.

(*S*)-2-(((3'-(3-Aminothiophen-2-yl)-[1,1'-biphenyl]-4-yl) methyl) (methyl) amino)-N-methyl-2-phenylacetamide **(MD4)**

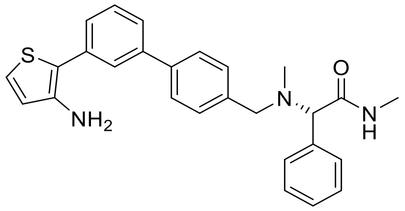

To a solution of **S5** (300 mg, 0.55 mmol) in DCM (10 mL) was added TFA (2 mL). The mixture was stirred at room temperature for 3 hours. Upon completion of the reaction monitored by LC-MS, the solvent was evaporated under the vacuum to afford the desired product **MD4** (239.7 mg, 0.54 mmol, 98% yield).

LC-MS (ESI+) *m/z* calculated for $[C_{27}H_{28}N_2O_2S]^+$: 442.2; found, 442.4.

^{1}H NMR (500 MHz, DMSO-d_6) δ 10.37 (s, 2H), 9.06 (s, 1H), 8.04 (s, 1H), 7.94 (d, J = 7.6 Hz, 2H), 7.77 (d, J = 7.2 Hz, 1H), 7.73 – 7.56 (m, 7H), 7.55 – 7.43 (m, 3H), 7.23 (d, J = 5.4 Hz, 1H), 5.11 (s, 1H), 4.42 (d, J = 67.6 Hz, 2H), 2.65 (d, J = 4.5 Hz, 3H), 2.38 (s, 3H). **^{13}C NMR** (126 MHz, DMSO) δ 166.44, 140.57, 140.13, 132.40, 131.97, 130.90, 130.13, 129.96, 129.59, 129.15, 128.84, 128.79, 127.86, 127.26, 126.60, 126.29, 126.06, 124.87, 70.14, 66.35, 58.66, 39.86, 25.85.

Synthesis of linker-attached RNase L binders (L1-L9)

General Procedure A (acid-amine coupling and Boc group de-protection):

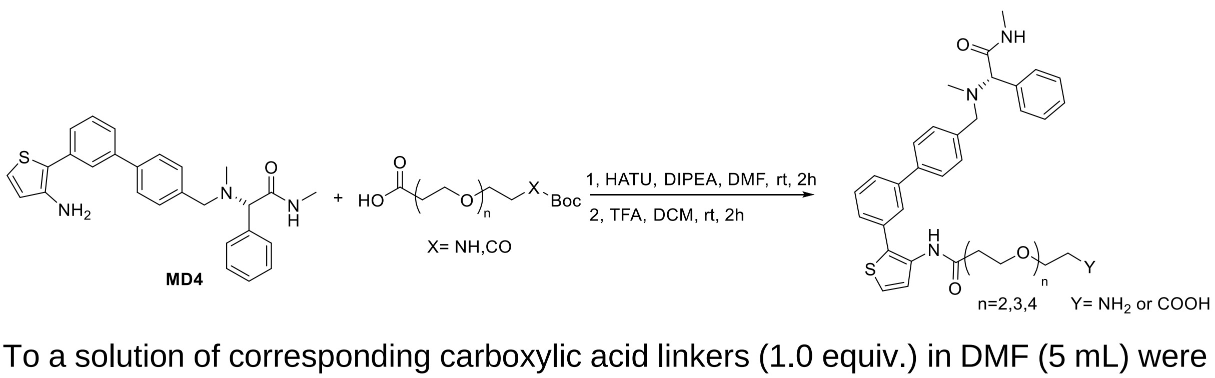

To a solution of corresponding carboxylic acid linkers (1.0 equiv.) in DMF (5 mL) were added HATU (38.7 mg, 0.10 mmol, 1.5 equiv.) and DIPEA (43.9 mg, 0.34 mmol, 5.0

equiv.). The mixture was stirred at room temperature for 5 min, followed by the addition of **MD4** (30.0 mg, 67.9 µmol, 1.0 equiv.). The reaction mixture was stirred for extra 3 hours. Upon completion of the reaction monitored by LC-MS, the solvent was evaporated under the high vacuum and residue was purified by the flash column chromatograph (PE: EA= 4:1 to 1 :1) to afford the desired intermediate. The intermediate was dissolved in DCM (5 mL), followed by the addition of TFA (1.5 mL). The reaction mixture was stirred at room temperature for 3 hours. Upon completion of the reaction monitored by LC-MS, the solvent was evaporated under the vacuum to afford the desired product, which was used in the next step without further purification.

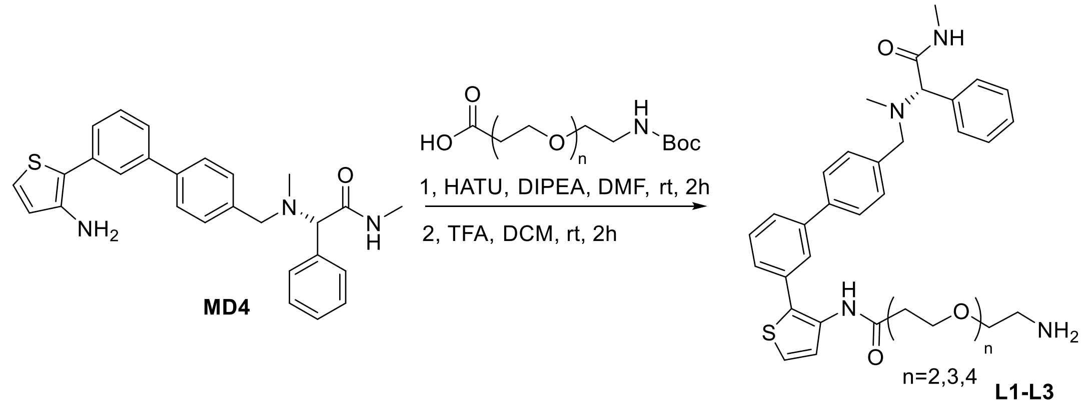

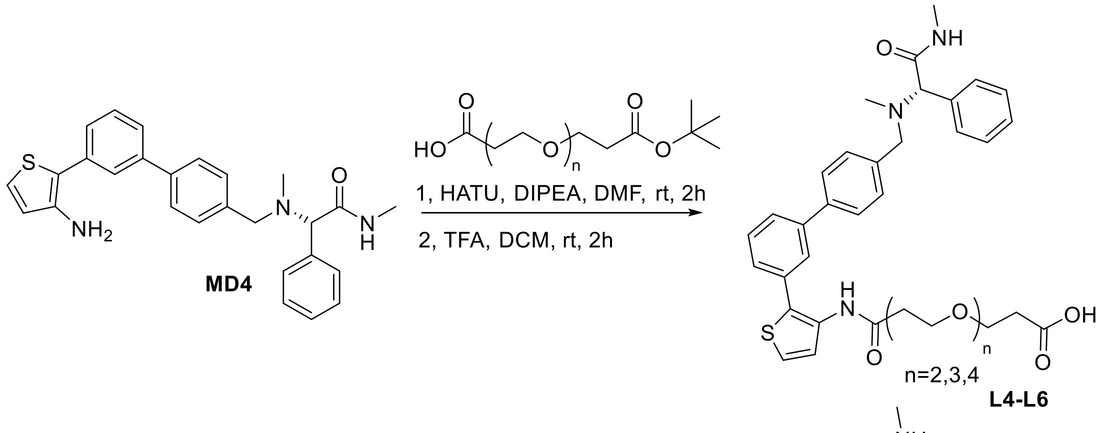

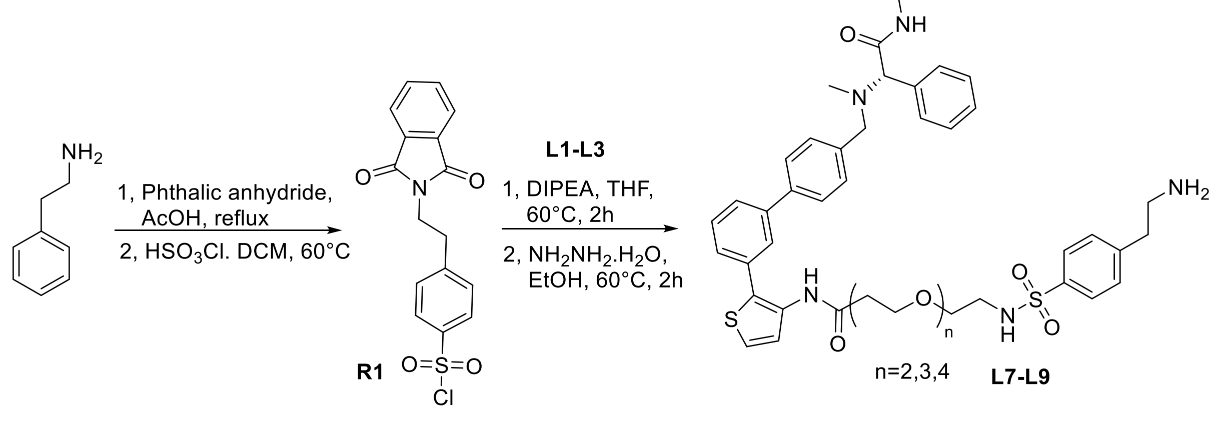

(*S*)-3-(2-(2-Aminoethoxy) ethoxy)-N-(2-(4'-((methyl (2-(methylamino)-2-oxo-1-phenylethyl) amino) methyl)-[1,1'-biphenyl]-3-yl) thiophen-3-yl) propenamide **(L1)**

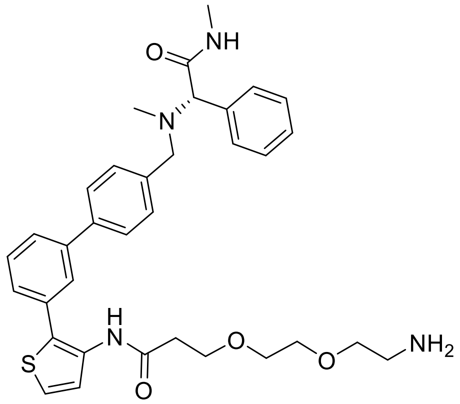

Following the **General Procedure A**, the title product **L1** was obtained as a light-yellow oil (28.9 mg, 48.1 µmol, 70.8% yield over two steps).

LC-MS (ESI+) *m/z* calculated for $[C_{34}H_{41}N_4O_4S]^+$: 601.3, found $[M + H]^+$: 601.2.

(*S*)-3-(2-(2-(2-Aminoethoxy) ethoxy) ethoxy)-N-(2-(4'-((methyl (2-(methylamino)-2-oxo-1-phenylethyl) amino) methyl)-[1,1'-biphenyl]-3-yl) thiophen-3-yl) propenamide **(L2)**

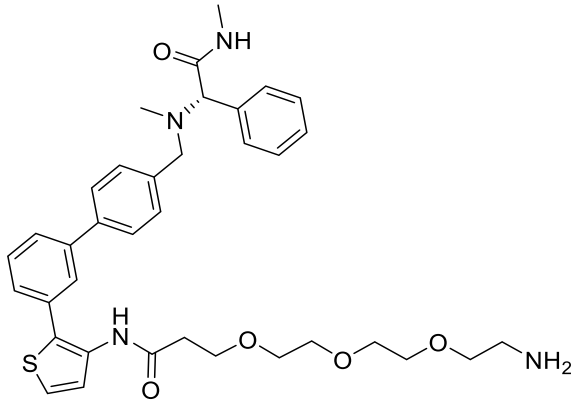

Following the **General Procedure A**, the title product **L2** was obtained as a light-yellow oil (32.6 mg, 50.6 µmol, 74.4% yield over two steps).

LC-MS (ESI+) *m/z* calculated for $[C_{36}H_{45}N_4O_5S]^+$: 645.3; found, 645.2.

(*S*)-1-Amino-*N*-(2-(4'-((methyl(2-(methylamino)-2-oxo-1-phenylethyl) amino) methyl)-[1,1'-biphenyl]-3-yl) thiophen-3-yl)-3,6,9,12-tetraoxapentadecan-15-amide **(L3)**

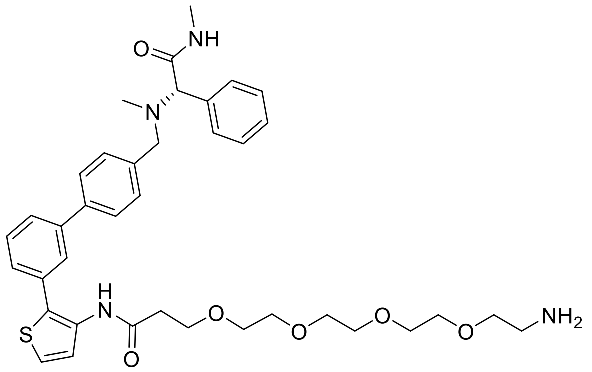

Following the **General Procedure A**, the title product **L3** was obtained as a light-yellow oil (42.3 mg, 61.4 µmol, 90.4% yield over two steps).

LC-MS (ESI+) *m/z* calculated for [$C_{38}H_{49}N_4O_6S$] $^+$: 689.3; found, 689.2.

(*S*)-3-(2-(3-((2-(4'-((Methyl (2-(methylamino)-2-oxo-1-phenylethyl) amino) methyl)-[1,1'-biphenyl]-3-yl) thiophen-3-yl) amino)-3-oxopropoxy) ethoxy) propanoic acid **(L4)**

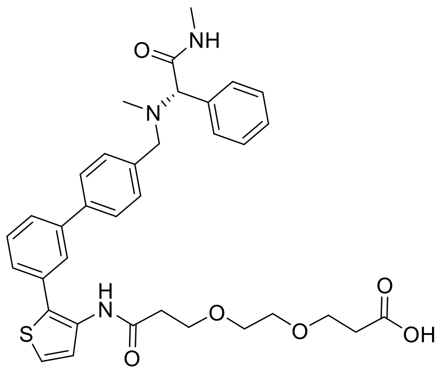

Following the **General Procedure A**, the title product **L4** was obtained as a light-yellow oil (34,6 mg, 54.9 µmol, 80,9% yield over two steps).

LC-MS (ESI+) *m/z* calculated for [$C_{35}H_{40}N_3O_6S$] $^+$: 630.3; found,630.0.

(*S*)-3-(2-(2-(3-((2-(4'-((Methyl (2-(methylamino)-2-oxo-1-phenylethyl) amino) methyl)-[1,1'-biphenyl]-3-yl) thiophen-3-yl) amino)-3-oxopropoxy) ethoxy) ethoxy) propanoic acid **(L5)**

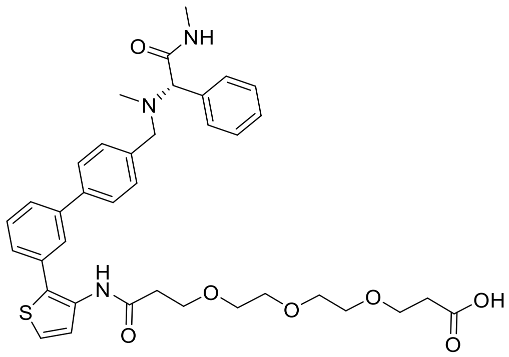

Following the **General Procedure A**, the title product **L5** was obtained as a light-yellow oil (38.8 mg, 57.6 µmol, 84.8% yield over two steps).

LC-MS (ESI+) *m/z* calculated for $[C_{37}H_{44}N_3O_7S]^+$: 674.3; found, 674.2.

(*S*)-16-((2-(4'-((Methyl (2-(methylamino)-2-oxo-1-phenylethyl) amino) methyl)-[1,1'-biphenyl]-3-yl) thiophen-3-yl) amino)-16-oxo-4,7,10,13-tetraoxahexadecanoic acid **(L6)**

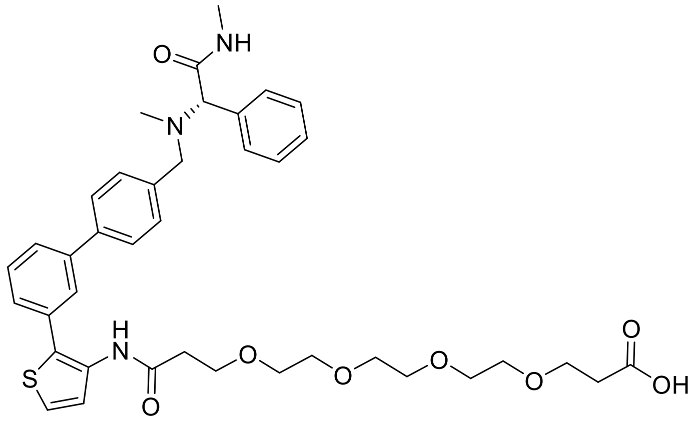

Following the **General Procedure A**, the title product **L6** was obtained as a light-yellow oil (39.4 mg, 54.9 µmol, 80.8% yield over two steps).

LC-MS (ESI+) *m/z* calculated for $[C_{39}H_{48}N_3O_8S]^+$: 718.3; found,718.2.

4-(2-(1,3-Dioxoisoindolin-2-yl) ethyl) benzenesulfonyl chloride **(R1)**

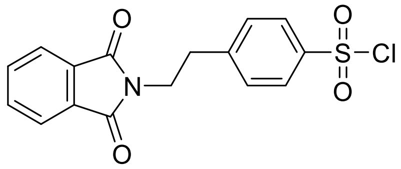

To a solution of 2-phenylethan-1-amine (3.0 g, 24.76 mmol) in acetic acid (30 mL) was added isobenzofuran-1,3-dione (4.65 g, 30.95 mmol). The reaction mixture was stirred under reflux for 3 hours. After checking for the completion of the reaction, the mixture was poured into the water (150 mL), the resulting white solid was filtered, and washed with H2O (3 × 20 mL). The filter residue was dried under the vacuum and used in the next step without further purification. The obtained residue was mixed with 2-phenethylisoindoline-1,3-dione (2.0 g, 7.96 mmol) and DCM (20 mL). Chlorosulfuric acid (3.71 g, 31.84 mmol) was added slowly (drop by drop) to the resulting reaction mixture, which was stirred at 60 °C for 2 hours. Upon completion of the reaction monitored by LC-MS, the reaction mixture was poured into crushed ice. The mixture

was extracted with DCM (3 × 50 mL). The combined organic phase was dried over $MgSO_4$ and the solvent was evaporated under the vacuum. The residue was purified by flash column chromatography (PE: EA= 2:1) to afford the desired product **R1** (2.46 g, 7.03 mmol, 88.4% yield).

LC-MS (ESI+) *m/z* calculated for $[C_{16}H_{12}ClNO_4S+Na]^+$: 372.2; found, 372.2.

^{1}H NMR (600 MHz, DMSO-d6) δ 7.81 (dd, J = 8.6, 5.9 Hz, 4H), 7.50 (d, J = 8.0 Hz, 2H), 7.16 (d, J = 7.9 Hz, 2H), 3.80 (t, J = 7.3 Hz, 2H), 2.92 (t, J = 7.3 Hz, 2H). **^{13}C NMR** (151 MHz, DMSO-d6) δ 167.79, 145.90, 139.11, 134.54, 131.55, 128.20, 125.81, 123.17, 38.80, 33.53.

General Procedure B (Sulfonamide formation and Succinamide de-protection):

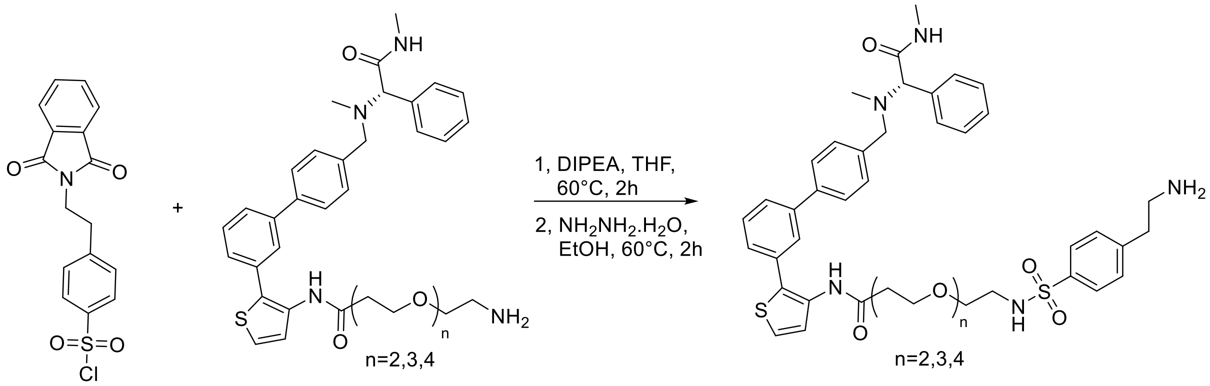

R1 **L1-L3** **L7-L9**

To a solution of the corresponding compound **L1-L3** (1.0 equiv.) in THF (5 mL) was added **R1** (20.0 mg, 31.02 µmol). The reaction mixture was stirred at 60 °C for 2 hours. Upon completion of the reaction monitored by LC-MS, the solvent was evaporated under vacuum and the residue was purified by flash column chromatograph (PE: EA= 2:1) to afford the desired sulfonamide intermediate.

The obtained sulfonamide intermediate (1.0 equiv.) was dissolved in EtOH (5 mL) and hydrazine (30.0 equiv.). The reaction mixture was stirred at 60 °C for 2 hours. Upon completion of the reaction monitored by LC-MS, the solvent was evaporated under vacuum. The residue suspended in water (15 mL) and the mixture was extracted with DCM (3 × 10 mL). The combined organic phases were removed under the high vacuum to obtain the desired product, which was used in the next step without further purification.

(*S*)-3-(2-(2-((4-(2-Aminoethyl) phenyl) sulfonamido) ethoxy) ethoxy)-N-(2-(4'-((methyl(2-(methylamino)-2-oxo-1-phenylethyl) amino) methyl)-[1,1'-biphenyl]-3-yl) thiophen-3-yl) propenamide **(L7)**

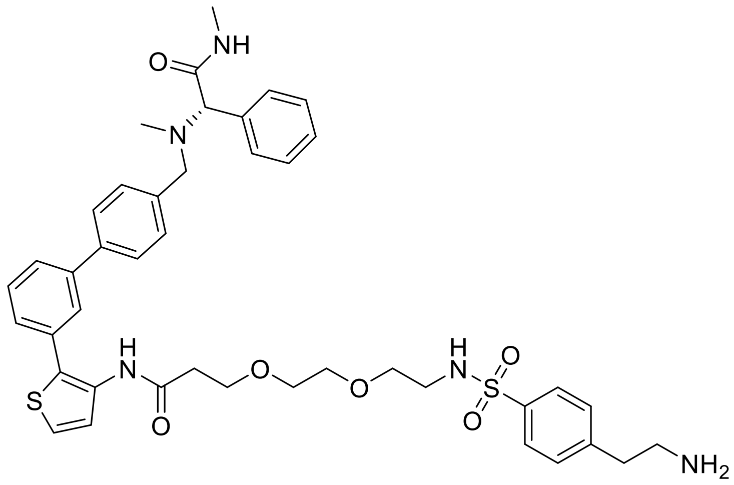

Following the **General Procedure B**, the title product **L7** was obtained as a colorless oil (17.8 mg, 22.7 µmol, 68.2% yield over two steps).

LC-MS (ESI+) *m/z* calculated for $[C_{42}H_{50}N_5O_6S_2]^+$: 784.3; found, 784.1.

(*S*)-3-(2-(2-(2-((4-(2-Aminoethyl) phenyl) sulfonamido) ethoxy) ethoxy) ethoxy)-N-(2-(4'-((methyl (2-(methylamino)-2-oxo-1-phenylethyl) amino) methyl)-[1,1'-biphenyl]-3-yl) thiophen-3-yl) propanamide **(L8)**

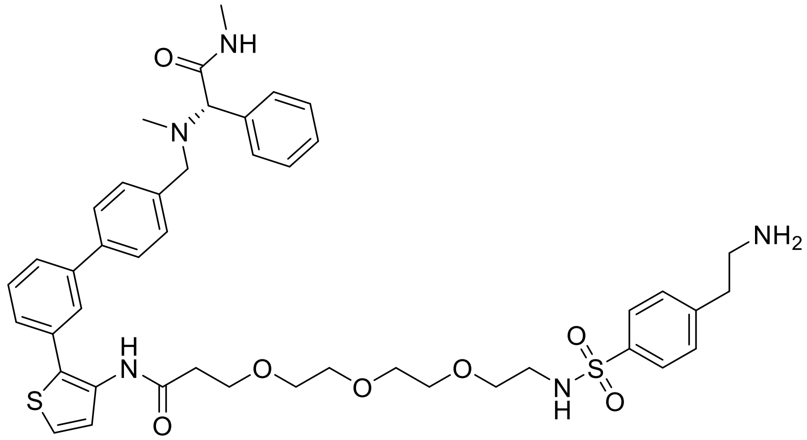

Following the **General Procedure B**, the title product **L8** was obtained as a colorless oil (21.6 mg, 21.1 µmol, 84.1% yield over two steps).

LC-MS (ESI+) *m/z* calculated for $[C_{44}H_{54}N_5O_7S_2]^+$: 828.3; found, 828.4.

(*S*)-1-((4-(2-Aminoethyl) phenyl) sulfonamido)-N-(2-(4'-((methyl(2-(methylamino)-2-oxo-1-phenylethyl) amino) methyl)-[1,1'-biphenyl]-3-yl) thiophen-3-yl)-3,6,9,12-tetraoxapentadecan-15-amide **(L9)**

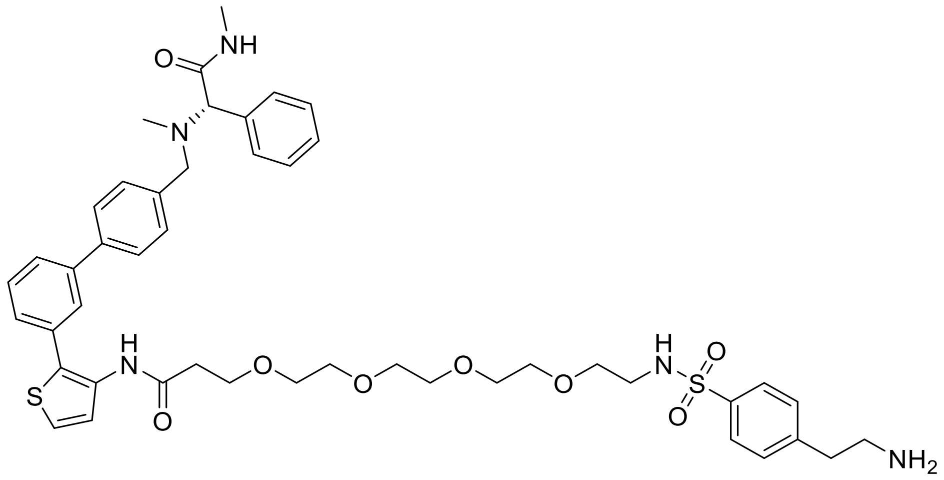

Following the **General Procedure B**, the title product **L9** was obtained as a colorless oil (19.8 mg, 19.8 µmol, 68.1% yield over two steps).

LC-MS (ESI+) *m/z* calculated for $[C_{46}H_{58}N_5O_8S_2]^+$: 872.4; found, 872.3.

Synthesis of NRAS RIBOTACs

Series SN

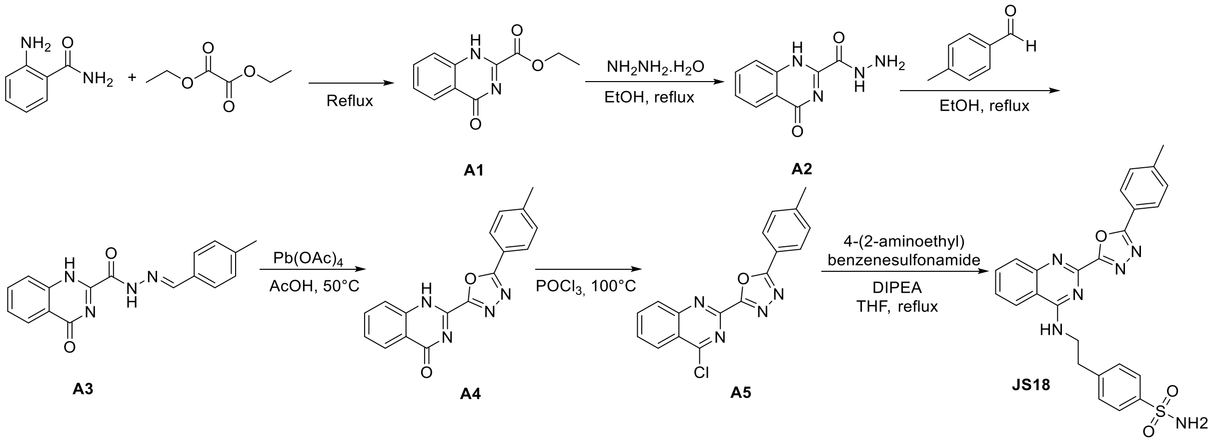

Ethyl 4-oxo-1,4-dihydroquinazoline-2-carboxylate **(A1)**

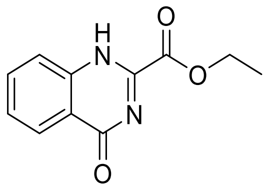

A reaction mixture of 2-aminobenzamide (10.0 g, 73.5 mmol) in diethyl oxalate (60 mL) was stirred for 8 hours in reflux. The solvent was partially removed under high

vacuum, followed by the addition of EtOH (80 mL). The resulting precipitate was then filtered to obtain **A1** as a white solid (12.6 g, 57.7 mol, 78.6% yield), which was used in next step without further purification.

LC-MS (ESI +) *m/z* calculated for $[C_{11}H_{11}N_2O_3]^+$: 219.1; found, 219.1.

^{1}H NMR (700 MHz, DMSO-d_6) δ 12.63 (s, 1H), 8.18 (dd, *J* = 8.0, 1.5 Hz, 1H), 7.90 (t, *J* = 8.4 Hz, 1H), 7.83 (dd, *J* = 8.2, 1.1 Hz, 1H), 7.65 (t, *J* = 8.1 Hz, 1H), 4.39 (q, *J* = 7.1 Hz, 2H), 1.36 (t, *J* = 7.1 Hz, 3H).**^{13}C NMR** (176 MHz, DMSO-d_6) δ 161.14, 160.14, 147.30, 143.52, 134.95, 128.82, 128.48, 126.15, 123.00, 62.81, 13.93.

4-Oxo-1,4-dihydroquinazoline-2-carbohydrazide **(A2)**

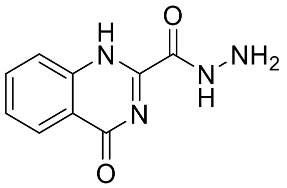

To a mixture of **A1** (10.0 g, 45.8 mmol) in EtOH (110 mL) was added hydrazine hydrate (100 mL). The resulting mixture was stirred at room temperature for 1 hour. Then, the temperature was raised to reflux for 2 hours. After the reaction mixture was cooled down to the room temperature, the resulting precipitate was filtered and washed with EtOH (3 × 20mL) to afford a white solid as **A2** (8.9 g, 43.6 mmol, 95.1% yield), which was used in the next step without further purification.

LC-MS (ESI +) *m/z* calculated for $[C_9H_9N_4O_2]^+$: 205.1; found, 205.0.

^{1}H NMR (700 MHz, DMSO-d_6) δ 12.25 (s, 1H), 10.27 (s, 1H), 8.17 (d, *J* = 7.8 Hz, 1H), 7.88 (t, *J* = 7.7 Hz, 1H), 7.75 (d, *J* = 8.1 Hz, 1H), 7.61 (t, *J* = 7.5 Hz, 1H). **^{13}C NMR** (176 MHz, DMSO-d_6) δ 160.96, 158.00, 147.27, 145.65, 134.85, 128.04, 127.81, 126.19, 122.69.

(*E*)-*N'*-(4-Methylbenzylidene)-4-oxo-1,4-dihydroquinazoline-2-carbohydrazide **(A3)**

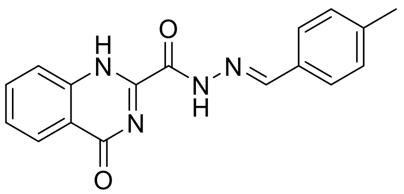

To a solution of **A2** (3.0 g, 14.7 mmol) in in EtOH (60 mL) was added *p*-Tolualdehyde (1.8 g, 14.7 mmol). The reaction mixture was stirred under reflux for 3 hours. Upon completion of the reaction monitored by LC-MS, the mixture was cooled to the room

temperature. The resulting white solid was filtrated and washed with EtOH (3 × 10 mL) to obtain the title product **A3** (4.2 g, 13.7 mmol, 93.3% yield).

LC-MS (ESI +) *m/z* calculated for [$C_{17}H_{15}N_4O_2$] $^+$: 307.1; found, 307.1.

^{1}H NMR (700 MHz, DMSO-*d*$_6$) δ 12.38 (s, 1H), 12.17 (s, 1H), 8.55 (s, 1H), 8.11 (dd, *J* = 8.0, 1.5 Hz, 1H), 7.86 – 7.80 (m, 1H), 7.77 (d, *J* = 7.9 Hz, 1H), 7.58 – 7.53 (m, 3H), 7.21 (d, *J* = 7.9 Hz, 2H), 2.27 (s, 3H). **^{13}C NMR** (176 MHz, DMSO-*d*$_6$) δ 161.43, 156.67, 151.23, 147.44, 146.11, 141.05, 135.33, 131.71, 130.03, 128.78, 128.30, 127.89, 126.71, 123.35, 21.56.

2-(5-(*p*-Tolyl)-1,3,4-oxadiazol-2-yl) quinazolin-4(1H)-one **(A4)**

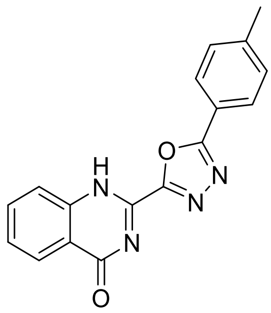

To a solution of **A3** (4.20 g, 13.8 mmol) that was partially dissolved in AcOH (100 mL) was added lead (VI) acetate (9.76 g, 22.0 mmol). The reaction mixture was stirred at 50 °C for 12 hours. Upon completion of the reaction monitored by LC-MS, the solvent was removed and a filtration with DCM (30 mL) was performed to afford a light-yellow solid. The resulting solid was stirred with water (30 mL) for 30 min and then filtered to afford the desired product **A4** (2.20 g, 7.25 mmol, 52.7% yield) as a white solid, which was used in the next step without further purification.

LC-MS (ESI +) *m/z* calculated for [$C_{17}H_{13}N_4O_2$] $^+$: 305.1; found, 305.2.

^{1}H NMR (700 MHz, Chloroform-*d*) δ 8.30 (dd, *J* = 7.9, 1.5 Hz, 1H), 8.07 (d, *J* = 8.2 Hz, 2H), 7.86 (dd, *J* = 8.1, 1.0 Hz, 1H), 7.79 (ddd, *J* = 8.3, 7.1, 1.5 Hz, 1H), 7.54 (ddd, *J* = 8.3, 7.1, 1.2 Hz, 1H), 7.31 (d, *J* = 8.0 Hz, 2H), 2.40 (s, 3H). **^{13}C NMR** (176 MHz, $CDCl_3$) δ 167.07, 160.84, 158.41, 147.83, 143.63, 138.88, 135.00, 129.95, 128.71, 128.53, 127.65, 126.85, 122.94, 119.96, 21.76.

2-(4-Chloroquinazolin-2-yl)-5-(*p*-tolyl)-1,3,4-oxadiazole **(A5)**

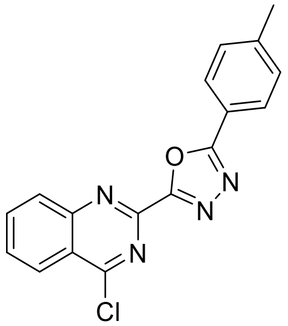

A mixture of **A4** (2.08 g, 6.84 mmol) dissolved in phosporus oxychloride (30 mL) was stirred at 100 °C for 12 hours. Upon completion of the reaction monitored by LC-MS, the reaction mixture was cooled to room temperature. After carefully poured on ice, the formation of a precipitate was observed. The precipitate was then filtered and purified by silica gel column chromatography (DCM: MeOH, 99:1) to obtain the title product **A5** (1.85 g, 5.73 mmol, 83.9% yield).

LC-MS (ESI +) *m/z* calculated for $[C_{17}H_{12}ClN_4O]^+$: 323.1; found, 323.0.

^{1}H NMR (700 MHz, Chloroform-*d*) δ 8.31 (dd, *J* = 8.4, 1.3 Hz, 1H), 8.24 (d, *J* = 8.4 Hz, 1H), 8.11 (d, *J* = 8.2 Hz, 2H), 8.01 (ddd, *J* = 8.4, 6.9, 1.4 Hz, 1H), 7.79 (ddd, *J* = 8.2, 7.0, 1.1 Hz, 1H), 7.30 (d, *J* = 7.8 Hz, 2H), 2.39 (s, 3H). **^{13}C NMR** (176 MHz, $CDCl_3$) δ 166.55, 163.79, 162.15, 151.22, 148.16, 143.10, 135.97, 130.60, 129.85, 129.63, 127.63, 126.17, 123.80, 120.61, 21.76.

4-(2-((2-(5-(p-tolyl)-1,3,4-Oxadiazol-2-yl) quinazolin-4-yl) amino) ethyl) benzenesulfonamide (**JS18**)

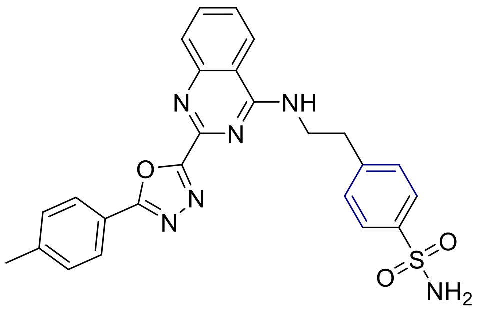

To a solution of **A5** (30.0 mg, 93.0 µmol) in THF (5 mL) was added DIPEA (18.0 mg, 139.4 µmol), followed by 4-(2-aminoethyl) benzenesulfonamide (18.6 mg, 93.0 µmol). The reaction mixture was stirred under reflux for 3 hours. Upon completion of the reaction monitored by LC-MS, the solvent was evaporated under the vacuum and the residue was purified by silica gel column chromatography (DCM: MeOH; 99:1-20:1) to afford the desired product **JS18** (42.3 mg, 87.0 µmol, 93.5% yield) as a white solid.

LC-MS (ESI +) *m/z* calculated for [$C_{25}H_{23}N_6O_3S$] $^+$: 487.2; found, 487.6.

^{1}H NMR (600 MHz, DMSO-*d*$_6$) δ 8.33 (dt, *J* = 8.4, 1.0 Hz, 1H), 8.03 (d, *J* = 8.1 Hz, 2H), 7.91 – 7.86 (m, 2H), 7.65 (ddd, *J* = 8.2, 6.0, 2.2 Hz, 1H), 7.58 (d, *J* = 8.3 Hz, 2H), 7.48 (d, *J* = 7.8 Hz, 2H), 7.46 (d, *J* = 8.2 Hz, 2H), 3.89 (dt, *J* = 8.3, 6.0 Hz, 2H), 3.16 (t, *J* = 7.4 Hz, 2H), 2.44 (s, 3H).

(S)-*N*-(2-(4'-((Methyl(2-(methylamino)-2-oxo-1-phenylethyl) amino) methyl)-[1,1'-biphenyl]-3-yl) thiophen-3-yl)-3-(2-(2-((4-(2-((2-(5-(p-tolyl)-1,3,4-oxadiazol-2-yl) quinazolin-4-yl) amino) ethyl) phenyl) sulfonamido) ethoxy) ethoxy) propenamide **(1)**

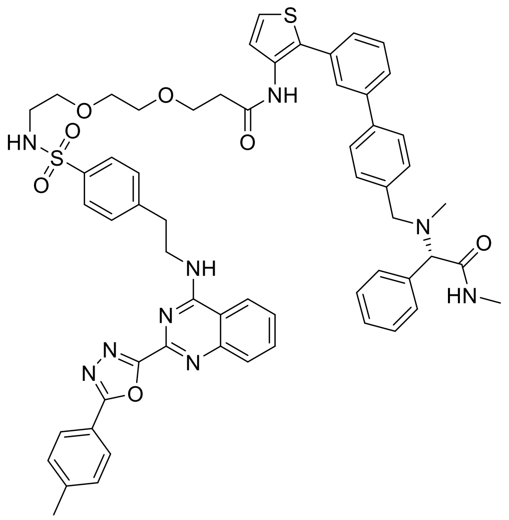

To a solution of **A5** (10.0 mg, 12.8 µmol) in DMF (3 mL) were added DIPEA (3.3 mg, 25.5 µmol) followed by **L7** (4.9 mg, 15.3 µmol). The reaction mixture was stirred at room temperature for 1 hour. Upon completion of the reaction monitored by LC-MS, the solvent was evaporated under the high vacuum. The residue was purified by preparative HPLC which described in **General Purification Procedure for NRAS RIBOTACs** to afford the desired product **1** (4.2 mg, 3.9 µmol, 30.7% yield) as a yellow solid.

HRMS: Calculated for [$C_{59}H_{60}N_9O_7S_2$]$^+$: 1070.4052; found, 1070.4077.

^{1}H NMR (700 MHz, Methanol-*d*$_4$) δ 8.04 (d, *J* = 8.1 Hz, 1H), 8.00 (d, *J* = 8.0 Hz, 2H), 7.83 (d, *J* = 8.1 Hz, 1H), 7.79 (ddd, *J* = 8.2, 6.8, 1.3 Hz, 1H), 7.65 – 7.60 (m, 3H), 7.58 (d, *J* = 8.3 Hz, 2H), 7.55 (ddd, *J* = 8.2, 6.8, 1.3 Hz, 1H), 7.52 – 7.45 (m, 5H), 7.43 (d, *J* = 8.2 Hz, 5H), 7.39 – 7.33 (m, 4H), 7.23 (d, *J* = 5.4 Hz, 1H), 7.20 (d, *J* = 5.4 Hz, 1H), 4.83 (s, 1H), 4.57-4.38 (br, 1H), 4.23-4.14 (br, 1H), 3.95 (t, *J* = 7.3 Hz, 2H), 3.62 (t, *J* = 6.0 Hz, 2H), 3.37 – 3.32 (m, 2H), 3.25 (s, 3H), 3.19 (t, *J* = 5.6 Hz, 2H), 3.07 (t, *J* =

7.3 Hz, 2H), 2.74 (t, J = 5.5 Hz, 2H), 2.69 (s, 3H), 2.49 (t, J = 6.0 Hz, 2H), 2.42-2.29 (br, 2H), 2.37 (s, 3H).

(S)-*N*-(2-(4'-((Methyl(2-(methylamino)-2-oxo-1-phenylethyl) amino) methyl)-[1,1'-biphenyl]-3-yl) thiophen-3-yl)-3-(2-(2-(2-((4-(2-((2-(5-(p-tolyl)-1,3,4-oxadiazol-2-yl) quinazolin-4-yl) amino) ethyl) phenyl) sulfonamido) ethoxy) ethoxy) ethoxy) propanamide **(2)**

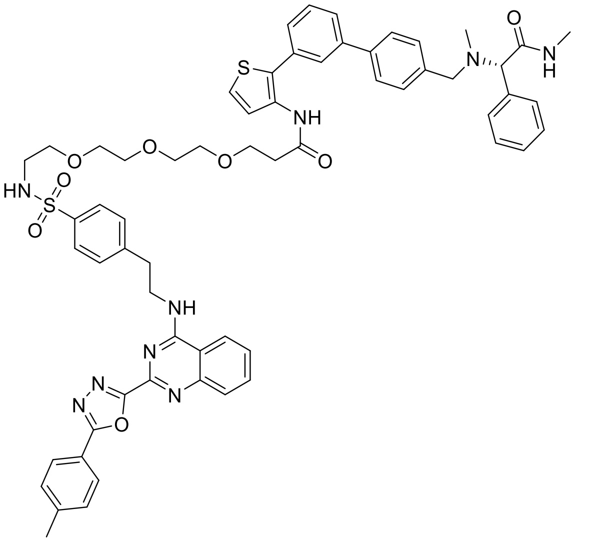

Following the same procedure of **1** in which **L1** was replaced with **L2**, the desired product **2** (5.8 mg, 5.2 µmol, 43.1% yield) was obtained as a yellow solid.

HRMS: Calculated for $[C_{61}H_{64}N_9O_8S_2]^+$: 1114.4314; found, 1114.4355.

^{1}H NMR (600 MHz, Methanol-d_4) δ 8.01 – 7.98 (m, 3H), 7.81 (dd, J = 8.7, 1.1 Hz, 1H), 7.75 (ddd, J = 8.3, 6.8, 1.3 Hz, 1H), 7.64 – 7.58 (m, 5H), 7.52 – 7.50 (m, 1H), 7.49 – 7.42 (m, 7H), 7.41 – 7.37 (m, 4H), 7.35 (dd, J = 10.4, 7.8 Hz, 3H), 7.24 (d, J = 5.4 Hz, 1H), 7.20 (d, J = 5.4 Hz, 1H), 4.20-4.06 (br, 1H), 3.93 (t, J = 7.3 Hz, 2H), 3.64 (t, J = 6.0 Hz, 2H), 3.40 (dd, J = 5.8, 3.4 Hz, 2H), 3.34 – 3.27 (m, 4H), 3.19 (d, J = 5.2 Hz, 3H), 3.06 (t, J = 7.3 Hz, 2H), 2.78 (t, J = 5.4 Hz, 2H), 2.69 (s, 3H), 2.49 (t, J = 6.0 Hz, 2H), 2.41-2.30 (br, 2H), 2.36 (s, 3H).

(S)-*N*-(2-(4'-((Methyl(2-(methylamino)-2-oxo-1-phenylethyl) amino) methyl)-[1,1'-biphenyl]-3-yl) thiophen-3-yl)-1-((4-(2-((2-(5-(p-tolyl)-1,3,4-oxadiazol-2-yl) quinazolin-4-yl) amino) ethyl) phenyl) sulfonamido)-3,6,9,12-tetraoxapentadecan-15-amide **(3)**

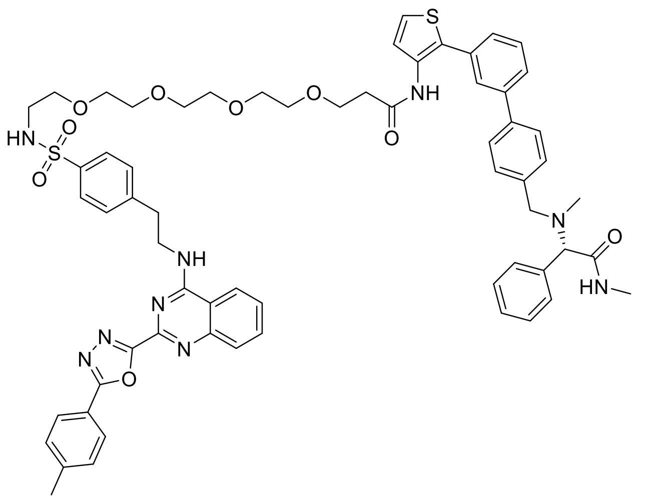

Following the same procedure of **1** in which **L1** was replaced with **L3**, the desired product **3** (3.9 mg, 3.4 µmol, 29.4% yield) was obtained as a yellow solid.

HRMS: Calculated for $[C_{63}H_{67}N_9O_9S_2+Na]^+$: 1180.4395; found $[M+Na]^+$, 1180.4406.

^{1}H NMR (700 MHz, Methanol-d_4) δ 8.04 (d, *J* = 8.2 Hz, 1H), 8.01 (d, *J* = 8.0 Hz, 2H), 7.83 (d, *J* = 8.1 Hz, 1H), 7.79 (ddd, *J* = 8.3, 6.8, 1.3 Hz, 1H), 7.67 – 7.63 (m, 3H), 7.61 (d, *J* = 8.2 Hz, 2H), 7.55 (ddd, *J* = 8.3, 6.8, 1.3 Hz, 1H), 7.48 (d, *J* = 7.3 Hz, 5H), 7.46 – 7.38 (m, 7H), 7.36 (d, *J* = 7.9 Hz, 2H), 7.26 (d, *J* = 5.4 Hz, 1H), 7.21 (d, *J* = 5.4 Hz, 1H), 4.83 (s, 1H), 4.64-4.38 (br, 1H), 4.27-4.14 (br, 1H), 3.97 (t, *J* = 7.3 Hz, 2H), 3.63 (t, *J* = 5.9 Hz, 2H), 3.39 (dd, *J* = 6.0, 3.5 Hz, 2H), 3.36 (dd, *J* = 6.1, 3.2 Hz, 2H), 3.35 – 3.30 (m, 6H), 3.26 – 3.22 (m, 6H), 3.08 (t, *J* = 7.3 Hz, 2H), 2.81 (t, *J* = 5.4 Hz, 2H), 2.69 (s, 3H), 2.48 (t, *J* = 5.9 Hz, 2H), 2.44-2.30 (br, 2H), 2.37 (s, 3H).

Series PC

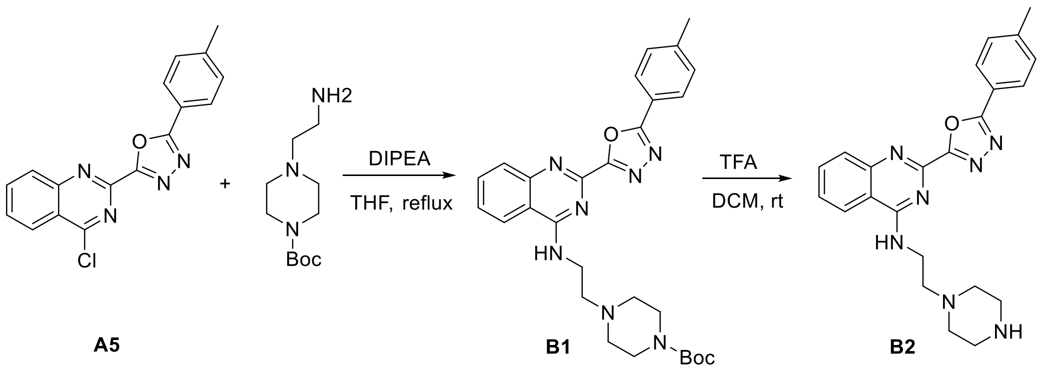

tert-Butyl 4-(2-((2-(5-(p-tolyl)-1,3,4-oxadiazol-2-yl) quinazolin-4-yl) amino) ethyl) piperazine-1-carboxylate **(B1)**

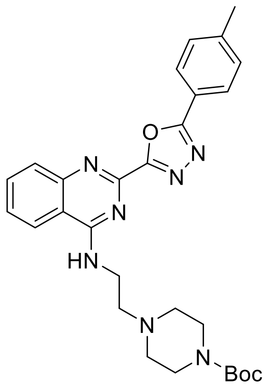

To a solution of **A5** (100.0 mg, 0.31 mmol) in THF (5 mL) were added tert-butyl 4-(2-aminoethyl) piperazine-1-carboxylate (85.3 mg, 0.37 mmol) and DIPEA (60.1 mg, 0.46 mmol). The reaction mixture was stirred under reflux for 3 hours. Upon completion of the reaction monitored by LC-MS, the solvent was evaporated under the vacuum and then the residue was purified by silica gel column chromatography (DCM: MeOH= 20:1) to afford the **B1** as a white solid (116.3 mg, 0.23 mmol, 72.8% yield).

LC-MS (ESI +) *m/z* calculated for [$C_{28}H_{34}N_7O_3$]: 516.3; found, 516.2.

N-(2-(Piperazin-1-yl) ethyl)-2-(5-(*p*-tolyl)-1,3,4-oxadiazol-2-yl) quinazolin-4-amine **(B2)**

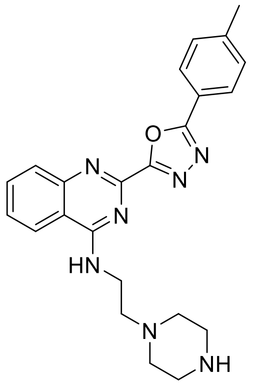

To a solution of **B1** (116.3 mg, 0.23 mmol) in DCM (10 mL) was added TFA (2 mL). The reaction mixture was stirred at room temperature for 3 hours. Upon completion of the reaction monitored by LC-MS, the solvent was removed through the vacuum to yield **B2** as a yellow oil (91.6 mg, 0.22 mmol, 97.7% yield), which was used in the next step without further purification.

LC-MS (ESI +) *m/z* calculated for [$C_{23}H_{26}N_7O$]: 416.2; found, 416.3.

(S)-*N*-(2-(4'-((Methyl(2-(methylamino)-2-oxo-1-phenylethyl) amino) methyl)-[1,1'-biphenyl]-3-yl) thiophen-3-yl)-3-(2-(3-oxo-3-(4-(2-((2-(5-(p-tolyl)-1,3,4-oxadiazol-2-yl) quinazolin-4-yl) amino) ethyl) piperazin-1-yl) propoxy) ethoxy) propanamide **(4)**

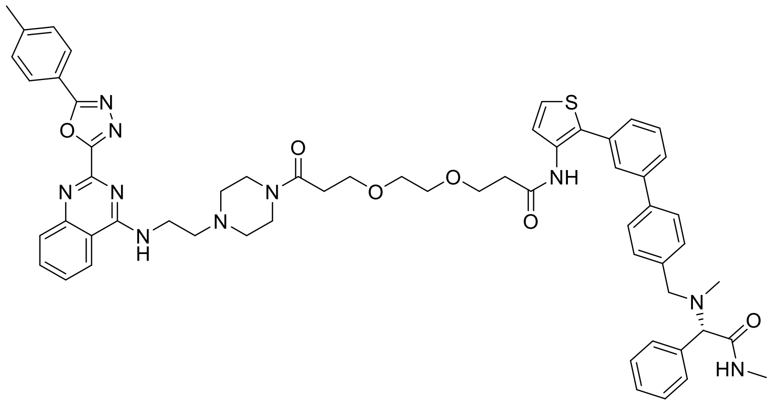

To a solution of **L4** (15.2 mg, 24.1 µmol) in DMF (3 mL) was added DIPEA (15.4 mg, 0.12 mmol) followed by HATU (13.7 mg, 36.1 µmol). After stirring the mixture at room temperature for 5 min, **B2** (10.0 mg, 24.1 µmol) was added to the reaction mixture, which was stirred for another hour. Upon completion of the reaction monitored by LC-MS, the solvent was evaporated under high vacuum and the residue was purified by preparative HPLC which described in **General Purification Procedure for NRAS RIBOTACs** to afford the desired product **4** (8.5 mg, 8.3 µmol, 34.4% yield) as a white solid.

HR-MS: Calculated for $[C_{58}H_{63}N_{10}O_6S]^+$: 1027.4642; found, 1027.4639.

^{1}H NMR (600 MHz, Methanol-*d*4) δ 8.08 (dt, *J* = 8.3, 0.9 Hz, 1H), 8.01 (d, *J* = 8.2 Hz, 2H), 7.93 – 7.89 (m, 1H), 7.86 (ddd, *J* = 8.3, 6.9, 1.3 Hz, 1H), 7.67 – 7.59 (m, 4H), 7.53 – 7.46 (m, 5H), 7.46 – 7.39 (m, 4H), 7.39 – 7.34 (m, 3H), 7.23 (d, *J* = 5.4 Hz, 1H), 7.11 (d, *J* = 5.4 Hz, 1H), 4.85 (s, 1H), 4.55-4.27 (br, 1H), 4.22-4.15 (br, 1H), 3.94 (t, *J* = 5.2 Hz, 2H), 3.62 (t, *J* = 5.7 Hz, 2H), 3.54 (q, *J* = 5.8 Hz, 2H), 3.44 (dq, *J* = 8.9, 4.8, 4.1 Hz, 4H), 3.40 – 3.37 (m, 2H), 2.70 (s, 3H), 2.47-2.41 (m, 4H), 2.37 (s, 3H). **^{13}C NMR** (151 MHz, MeOD) δ 173.18, 172.98, 168.37, 168.02, 164.74, 162.70, 162.54, 162.31, 150.27, 149.54, 145.21, 143.78, 142.00, 135.73, 135.02, 133.46, 133.15, 132.73, 132.25, 131.41, 130.97, 129.95, 129.78, 129.44, 128.93, 128.57, 128.10, 127.75, 124.85, 124.02, 121.84, 116.59, 73.00, 71.53, 71.40, 68.99, 68.05, 59.29, 53.68, 53.50, 43.76, 39.12, 37.79, 37.71, 34.52, 26.85, 21.88.

(S)-*N*-(2-(4'-((Methyl(2-(methylamino)-2-oxo-1-phenylethyl) amino) methyl)-[1,1'-biphenyl]-3-yl) thiophen-3-yl)-3-(2-(2-(3-oxo-3-(4-(2-((2-(5-(p-tolyl)-1,3,4-oxadiazol-2-

yl) quinazolin-4-yl) amino) ethyl) piperazin-1-yl) propoxy) ethoxy) ethoxy) propanamide **(5)**

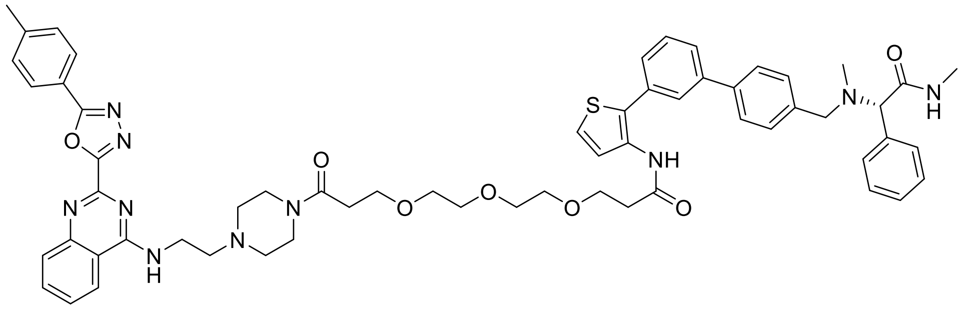

Following the same procedure of **4** in which **L4** was replaced with **L5**, the desired product **5** (5.8 mg, 5.4 µmol, 22.5% yield) was obtained as a white solid.

HR-MS: Calculated for $[C_{60}H_{67}N_{10}O_7S]^+$: 1071.4909; found, 1071.4869.

^{1}H NMR (500 MHz, Methanol-d_4) δ 8.08 – 8.05 (m, 1H), 8.04 – 8.01 (m, 2H), 7.92 (dd, *J* = 8.4, 1.3 Hz, 1H), 7.86 (ddd, *J* = 8.2, 6.9, 1.3 Hz, 1H), 7.64 (d, *J* = 7.9 Hz, 2H), 7.63 – 7.59 (m, 2H), 7.54 – 7.46 (m, 5H), 7.46 – 7.41 (m, 4H), 7.40 (d, *J* = 7.6 Hz, 1H), 7.37 – 7.34 (m, 2H), 7.24 (d, *J* = 5.4 Hz, 1H), 7.14 (d, *J* = 5.4 Hz, 1H), 4.84 (s, 1H), 4.54-4.30 (br, 1H), 4.24-4.15 (br, 1H), 3.95 (t, *J* = 4.7 Hz, 3H), 3.63 (t, *J* = 5.8 Hz, 2H), 3.60 – 3.53 (m, 3H), 3.49 – 3.38 (m, 11H), 2.70 (s, 3H), 2.45 (t, *J* = 5.9 Hz, 4H), 2.40-2.30 (m, 2H), 2.37 (s,3H). **^{13}C NMR** (126 MHz, MeOD) δ 171.61, 171.30, 166.48, 163.21, 161.16, 148.75, 148.03, 143.66, 142.27, 140.42, 134.16, 133.59, 131.91, 131.64, 131.59, 131.14, 130.68, 129.85, 129.45, 129.41, 128.21, 127.90, 127.50, 127.34, 127.02, 126.43, 126.12, 123.30, 122.45, 120.30, 115.04, 69.97, 69.82, 67.33, 66.68, 58.08, 56.08, 55.90, 52.39, 52.00, 51.07, 48.11, 42.30, 37.82, 36.29, 36.11, 32.88, 25.29, 20.32.

(S)-*N*-(2-(4'-((Methyl(2-(methylamino)-2-oxo-1-phenylethyl) amino) methyl)-[1,1'-biphenyl]-3-yl) thiophen-3-yl)-16-oxo-16-(4-(2-((2-(5-(p-tolyl)-1,3,4-oxadiazol-2-yl) quinazolin-4-yl) amino) ethyl) piperazin-1-yl)-4,7,10,13-tetraoxahexadecanamide **(6)**

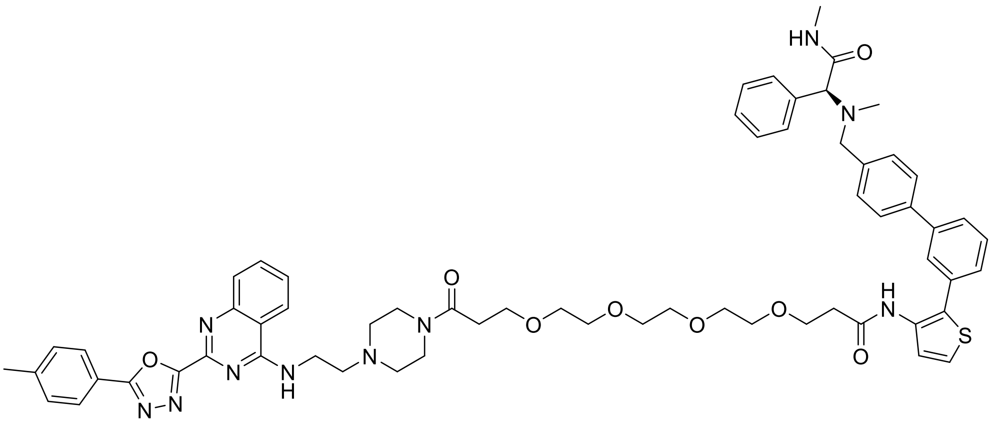

Following the same procedure of **4** in which **L4** was replaced with **L6**, the RIBOTAC **6** (8.8 mg, 7.9 µmol, 32.8% yield) was obtained as white solid.

HR-MS: Calculated for [$C_{62}H_{70}N_{10}O_8S$+Na] $^+$: 1137.4991; found [M+Na] $^+$, 1137.4951. **^{1}H NMR** (500 MHz, Methanol-d_4) δ 8.06 (dd, *J* = 8.3, 1.3 Hz, 1H), 8.05 – 8.02 (m, 2H), 7.92 (dd, *J* = 8.5, 1.3 Hz, 1H), 7.86 (ddd, *J* = 8.3, 6.9, 1.3 Hz, 1H), 7.65 (d, *J* = 7.9 Hz, 2H), 7.62 – 7.57 (m, 2H), 7.54 – 7.47 (m, 5H), 7.44 (ddq, *J* = 5.5, 3.9, 2.3 Hz, 4H), 7.40 (d, *J* = 7.6 Hz, 1H), 7.38 – 7.35 (m, 2H), 7.21 (d, *J* = 5.4 Hz, 1H), 7.11 (d, *J* = 5.4 Hz, 1H), 4.84 (s, 1H), 4.55-4.30 (br, 1H), 4.25-4.15 (br, 1H), 3.95 (s, 3H), 3.62 (q, *J* = 5.7 Hz, 5H), 3.49 – 3.38 (m, 16H), 2.70 (s, 3H), 2.47-2.40 (m, 4H), 2.38 (s, 3H).2.33-2.39 (m, 2H). **^{13}C NMR** (126 MHz, MeOD) δ 171.69, 171.32, 163.20, 161.19, 160.70, 160.41, 148.73, 148.06, 143.67, 140.40, 134.15, 133.62, 131.94, 131.76, 131.06, 130.71, 129.87, 129.42, 128.20, 127.88, 127.35, 127.02, 126.50, 126.39, 126.12, 123.26, 122.45, 120.30, 115.02, 70.12, 69.99, 69.91, 69.88, 69.81, 67.40, 66.63, 58.32, 52.56, 52.00, 48.11, 47.94, 47.77, 47.60, 47.43, 47.26, 47.09, 36.26, 36.03, 32.91, 25.30, 20.32.

Series BS

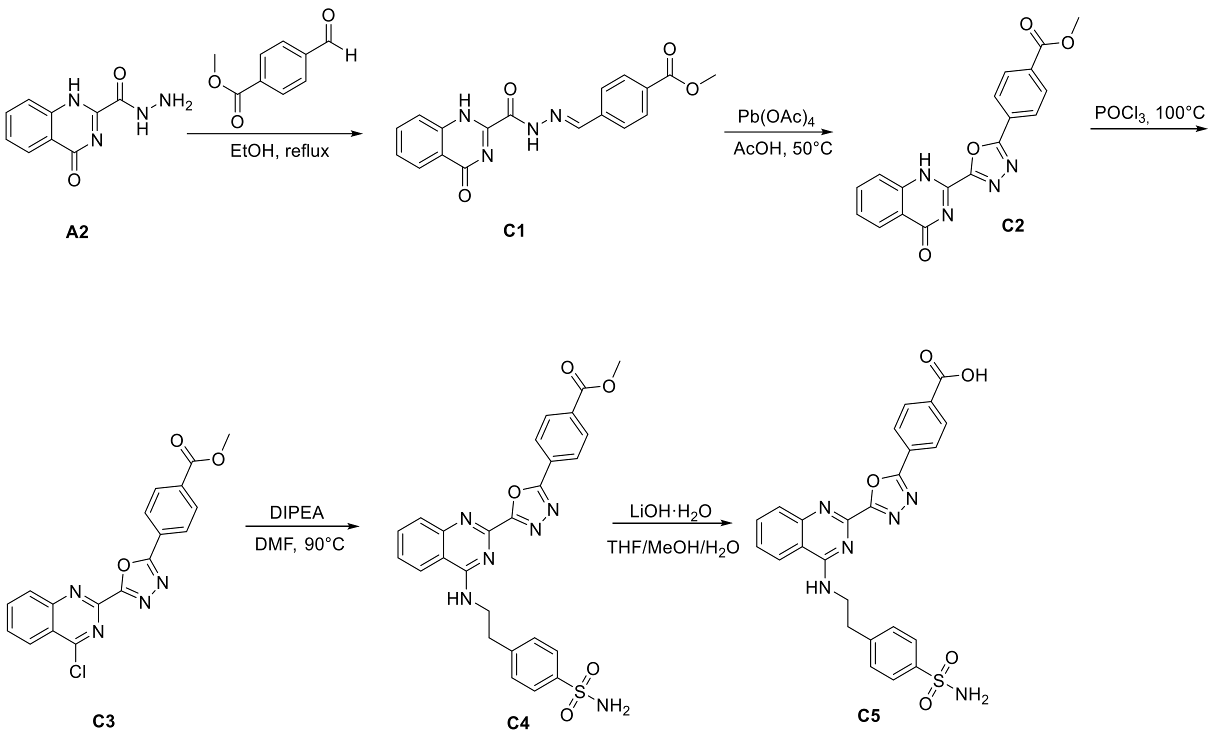

Methyl (*E*)-4-((2-(4-oxo-1,4-dihydroquinazoline-2 -carbonyl) hydrazineylidene) methyl) benzoate **(C1)**

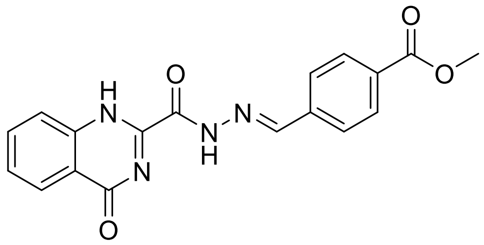

To a solution of **A2** (5.0 g, 24.5 mmol) in EtOH (100 mL) was added p-methyl 4-formylbenzoate (4.02 g, 24.5 mmol). The mixture was stirred under reflux for 3 hours. Upon completion of the reaction monitored by LC-MS, the reaction mixture was cooled to the room temperature, then the resulting solid was filtrated and washed with EtOH (3 × 10 mL) to obtain the title product **C1** (8.02 g, 22.86 mmol, 93.4% yield) as a white solid, which was used in the next step without further purification.

LC-MS (ESI+) *m/z* calculated for $[C_{18}H_{15}N_4O_4]^+$: 351.1; found, 351.0.

^{1}H NMR (600 MHz, DMSO-d6) δ 12.51 (s, 1H), 12.47 (s, 1H), 8.75 (s, 1H), 8.20 (d, J = 7.8 Hz, 1H), 8.04 (d, J = 8.3 Hz, 2H), 7.92 (t, J = 7.5 Hz, 1H), 7.89 – 7.86 (m, 3H), 7.64 (t, J = 7.5 Hz, 1H), 3.87 (s, 3H). **^{13}C NMR** (151 MHz, DMSO-d6) δ 165.78, 160.85, 156.46, 149.28, 146.92, 145.44, 138.36, 134.84, 130.83, 129.70, 128.37, 127.82, 127.53, 126.25, 123.01, 52.30.

Methyl 4-(5-(4-oxo-1,4-dihydroquinazolin-2-yl)-1,3,4-oxadiazol-2-yl) benzoate **(C2)**

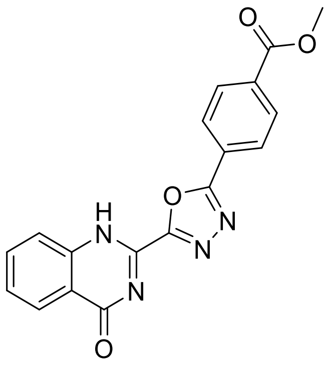

To a solution of **C1** (1.0 g, 2.86 mmol) in AcOH (20 mL) was added lead (VI) acetate (2.03 g, 4.58 mmol). The mixture was stirred at 50 °C overnight. Upon completion of the reaction monitored by LC-MS, the reaction mixture was cooled to the room temperature. The resulting solid was filtered and washed with ethyl acetate (3 × 10 mL). The filtrate was evaporated under the vacuum. Water (30 mL) was then added and extracted with DCM (3 × 30 mL). The combined organic phase was dried over MgSO4 and evaporated under the vacuum. Ethyl acetate (5 mL) was added into the residue, the mixture was stirred at room temperature for 10 min and then filtered to afford the title product **C2** (149.0 mg, 0.43 mmol, 14.9% yield) as a white solid.

LC-MS (ESI+) *m/z* calculated for $[C_{18}H_{13}N_4O_4]^+$: 349.1; found, 349.2.

Methyl 4-(5-(4-chloroquinazolin-2-yl)-1,3,4-oxadiazol-2-yl) benzoate **(C3)**

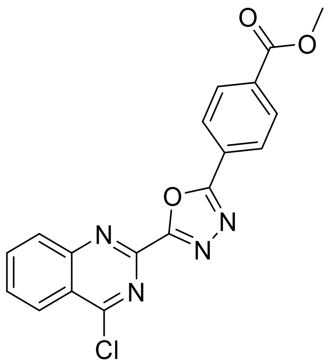

A mixture of **C2** (149.0 mg, 0.43 mmol) in phosporus oxychloride (5 mL) was stirred at 100 °C for 12 hours. The mixture was then cooled to room temperature and then carefully poured on ice. The formation of a precipitate could be observed. The precipitate was then filtered and purified by silica gel column chromatography (DCM: MeOH, 99:1) to obtain the title product **C3** (123.8 mg, 0.34 mmol, 78.9% yield).

LC-MS (ESI+) *m/z* calculated for $[C_{18}H_{12}ClN_4O_3]^+$: 367.1; found, 367.0.

^{1}H NMR (500 MHz, DMSO-d_6) δ 8.46 – 8.42 (m, 1H), 8.33 (dd, *J* = 8.6, 7.1 Hz, 3H), 8.28 (ddd, *J* = 8.4, 6.9, 1.4 Hz, 1H), 8.24 (d, *J* = 8.6 Hz, 2H), 8.05 (ddd, *J* = 8.2, 6.9, 1.2 Hz, 1H), 3.93 (s, 3H).

Methyl 4-(5-(4-((4-sulfamoylphenethyl) amino) quinazolin-2-yl)-1,3,4-oxadiazol-2-yl) benzoate **(C4)**

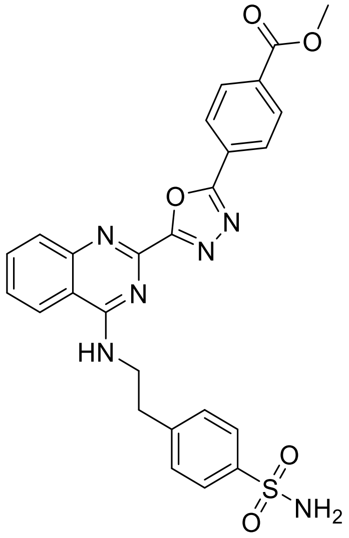

To a solution of **C3** (15.0 mg, 0.04 mmol) in DMF (3 mL) was added DIPEA (10.6 mg, 0.08 mmol) followed by 4-(2-aminoethyl) benzenesulfonamide (9.0 mg, 0.04 mmol). The reaction mixture was stirred at 90 °C for 3 hours. Upon completion of the reaction monitored by LC-MS, the solvent was evaporated under vacuum and the residue was purified by flash column chromatograph (DCM: MeOH= 40:1 to 20:1) to afford the desired product **C4** as a white solid (18.2 mg, 0.03 mmol, 83.9% yield).

LC-MS (ESI+) *m/z* calculated for $[C_{26}H_{23}N_6O_5S]^+$: 531.1; found, 531.1.

4-(5-(4-((4-Sulfamoylphenethyl) amino) quinazolin-2-yl)-1,3,4-oxadiazol-2-yl) benzoic acid **(C5)**

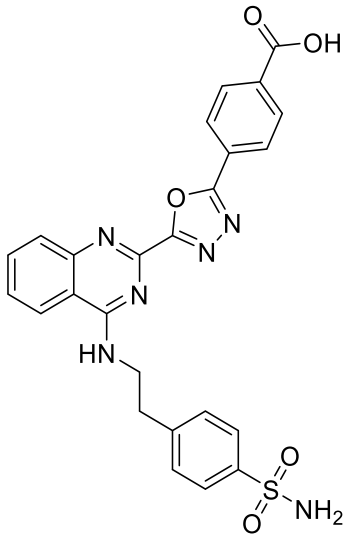

To a solution of **C4** (10.0 mg, 0.02 mmol) in THF/MeOH/H_2O (5 mL, 3:1:1) was added LiOH·H_2O (1.2 mg, 0.03 mmol). The reaction mixture was stirred at room temperature for 3 hours. Upon completion of the reaction monitored by LC-MS, the solvent was evaporated under the vacuum. Water (2 mL) was added to the residue and 1 M HCl was dropped to adjust the pH value to 5. The resulting solid was filtered and dried under the vacuum to afford the desired product **C5** (7.9 mg, 0.02 mmol, 81.1% yield) as a light-yellow solid, which was used in the next step without further purification.

LC-MS (ESI+) *m/z* calculated for $[C_{25}H_{21}N_6O_5S]^+$: 517.1; found, 517.0.

(S)-*N*-(2-(2-(3-((2-(4'-((Methyl(2-(methylamino)-2-oxo-1-phenylethyl) amino) methyl)-[1,1'-biphenyl]-3-yl) thiophen-3-yl) amino)-3-oxopropoxy) ethoxy) ethyl)-4-(5-(4-((4-sulfamoylphenethyl) amino) quinazolin-2-yl)-1,3,4-oxadiazol-2-yl) benzamide **(7)**

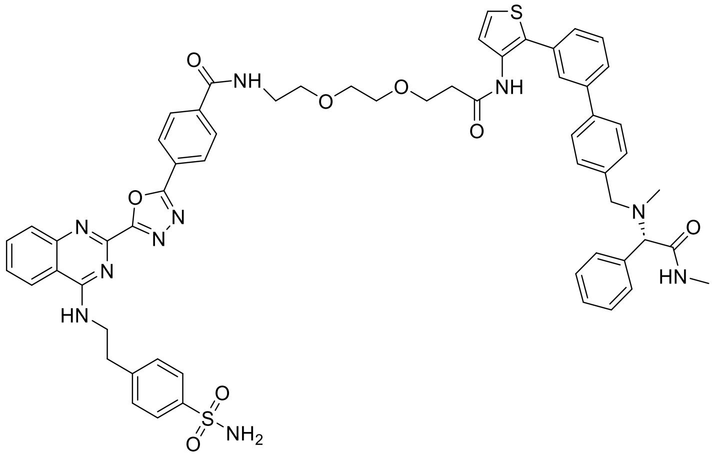

Following the same procedure of **8** in which **L2** was replaced with **L1**, the desired product **7** (7.6 mg, 6.9 µmol, 35.7% yield) was obtained as a white solid.

HR-MS: Calculated for $[C_{59}H_{59}N_{10}O_8S_2]^+$: 1099.3953; found, 1099.3925.

^{1}H NMR (700 MHz, Methanol-*d*$_4$) δ 8.09 (d, *J* = 8.0 Hz, 2H), 8.07 – 8.03 (m, 1H), 7.88 – 7.84 (m, 2H), 7.84 – 7.78 (m, 2H), 7.72 – 7.69 (m, 2H), 7.63 – 7.55 (m, 4H), 7.51 – 7.39 (m, 11H), 7.35 (t, *J* = 7.7 Hz, 1H), 7.26 (d, *J* = 5.4 Hz, 1H), 7.20 (d, *J* = 5.4 Hz, 1H), 4.83 (s, 1H), 4.54-4.44 (br, 1H), 4.22-4.13 (br, 1H), 4.01 – 3.95 (m, 2H), 3.71 (t, *J* = 5.9 Hz, 2H), 3.52 (td, *J* = 5.3, 2.8 Hz, 4H), 3.48 (dt, *J* = 4.4, 3.1 Hz, 2H), 3.43 (t, *J* = 5.5 Hz, 2H), 3.25 (s, 2H), 3.13 – 3.09 (m, 2H), 2.69 (s, 3H), 2.51 (t, *J* = 6.0 Hz, 2H), 2.44-2.29 (br, 2H).

(S)-*N*-(2-(2-(2-(3-((2-(4'-((Methyl(2-(methylamino)-2-oxo-1-phenylethyl) amino) methyl)-[1,1'-biphenyl]-3-yl) thiophen-3-yl) amino)-3-oxopropoxy) ethoxy) ethoxy) ethyl)-4-(5-(4-((4-sulfamoylphenethyl) amino) quinazolin-2-yl)-1,3,4-oxadiazol-2-yl) benzamide **(8)**

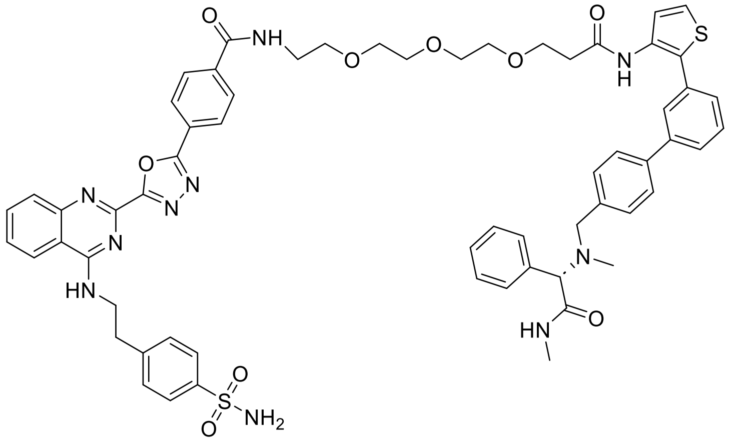

To a solution of **C5** (10.0 mg, 19.4 µmol) in DMF (3 mL) was added DIPEA (33.3 mg, 0.26 mmol) and HATU (9.5 mg, 29.0 µmol). The mixture was stirred at room temperature for 5 min, followed by the addition of **L2** (12.5 mg, 19.4 µmol). The reaction mixture was stirred for extra 1 hour. Upon completion of the reaction monitored by LC-MS, the solvent was evaporated under high vacuum and the residue was purified by preparative HPLC which described in **General Purification Procedure for NRAS RIBOTACs** to afford the desired product **8** (10.8 mg, 9.45 µmol, 48.8% yield) as a white solid.

HR-MS: Calculated for $[C_{61}H_{63}N_{10}O_9S_2]^+$: 1143.4215; found, 1143.4220.

^{1}H NMR (700 MHz, Methanol-*d*$_4$) δ 8.14 (d, *J* = 8.2 Hz, 2H), 8.05 (t, *J* = 7.8 Hz, 1H), 7.93 – 7.89 (m, 2H), 7.84 – 7.78 (m, 2H), 7.70 (d, *J* = 8.2 Hz, 2H), 7.65 – 7.59 (m, 3H), 7.56 (t, *J* = 7.5 Hz, 1H), 7.52 – 7.38 (m, 11H), 7.36 (t, *J* = 7.6 Hz, 1H), 7.24 (dd, *J* = 5.4, 2.0 Hz, 1H), 7.19 (d, *J* = 5.4 Hz, 1H), 4.83 (s, 1H), 4.57-4.38 (br, 1H), 4.23-4.13

(br, 1H), 3.97 (q, *J* = 9.2, 7.6 Hz, 2H), 3.65 (t, *J* = 6.0 Hz, 2H), 3.53 (t, *J* = 5.4 Hz, 2H), 3.47 (s, 2H), 3.47 – 3.45 (m, 4H), 3.43-3.41 (m, 4H), 3.12 – 3.07 (m, 2H), 2.77 (s, 3H), 2.69 (s, 3H), 2.49 (t, *J* = 6.0 Hz, 2H), 2.44-2.30 (br, 2H).

(S)-*N*-(15-((2-(4'-((Methyl(2-(methylamino)-2-oxo-1-phenylethyl) amino) methyl)-[1,1'-biphenyl]-3-yl) thiophen-3-yl) amino)-15-oxo-3,6,9,12-tetraoxapentadecyl)-4-(5-(4-((4-sulfamoylphenethyl) amino) quinazolin-2-yl)-1,3,4-oxadiazol-2-yl) benzamide **(9)**

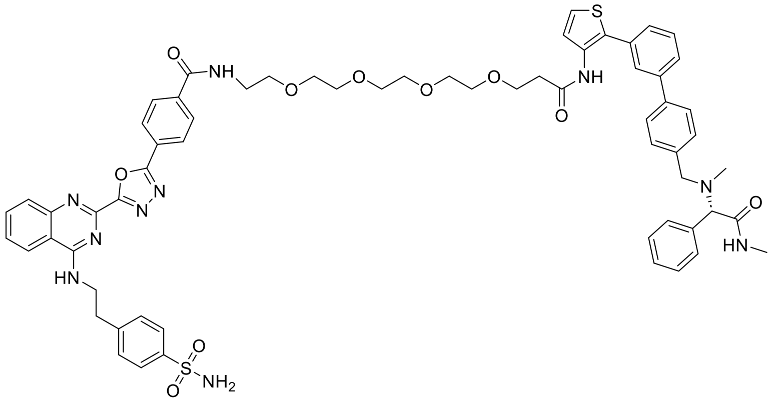

Following the same procedure of **7** in which **L2** was replaced with **L3**, **9** (9.5 mg, 8.0 µmol, 41.3% yield) was obtained as white solid.

HR-MS: Calculated for $[C_{59}H_{59}N_{10}O_8S_2]^+$: 1187.4478; found, 1187.4436.

^{1}H NMR (700 MHz, Methanol-*d*$_4$) δ 8.14 (d, *J* = 8.2 Hz, 2H), 8.06 – 8.02 (m, 1H), 7.92 (d, *J* = 8.4 Hz, 2H), 7.82 (dd, *J* = 8.4, 1.5 Hz, 1H), 7.79 (ddd, *J* = 8.2, 6.6, 1.3 Hz, 1H), 7.70 (d, *J* = 8.2 Hz, 2H), 7.65 – 7.60 (m, 3H), 7.55 (ddd, *J* = 8.2, 6.7, 1.4 Hz, 1H), 7.53 – 7.39 (m, 11H), 7.36 (t, *J* = 7.6 Hz, 1H), 7.24 (d, *J* = 5.4 Hz, 1H), 7.20 (d, *J* = 5.5 Hz, 1H), 4.84 (s, 1H), 4.60-4.40 (br, 1H), 4.26-4.12 (br, 1H), 3.97 – 3.93 (m, 2H), 3.63 (t, *J* = 5.9 Hz, 2H), 3.56 (t, *J* = 5.5 Hz, 2H), 3.52 – 3.47 (m, 6H), 3.43 – 3.41 (m, 2H), 3.39 (dq, *J* = 8.5, 4.0 Hz, 4H), 3.34 (dd, *J* = 5.9, 3.5 Hz, 2H), 3.25 (s, 2H), 3.08 (t, *J* = 7.5 Hz, 2H), 2.70 (s, 3H), 2.49 (t, *J* = 5.9 Hz, 2H), 2.46-2.28 (br, 2H). **^{13}C NMR** (176 MHz, MeOD) δ 171.64, 167.46, 166.76, 165.18, 163.34, 160.67, 160.47, 160.25, 148.34, 144.24, 142.35, 141.70, 140.30, 137.82, 133.77, 133.64, 131.94, 131.24, 131.19, 130.69, 129.42, 129.38, 129.33, 129.33, 128.15, 128.07, 127.77, 127.74, 127.37, 127.03, 126.59, 126.19, 126.08, 125.95, 125.78, 123.18, 122.26, 114.71, 70.17, 70.08, 70.05, 69.90, 69.87, 69.83, 68.98, 66.67, 47.97, 42.34, 39.79, 36.46, 34.58, 25.32.

Series BM

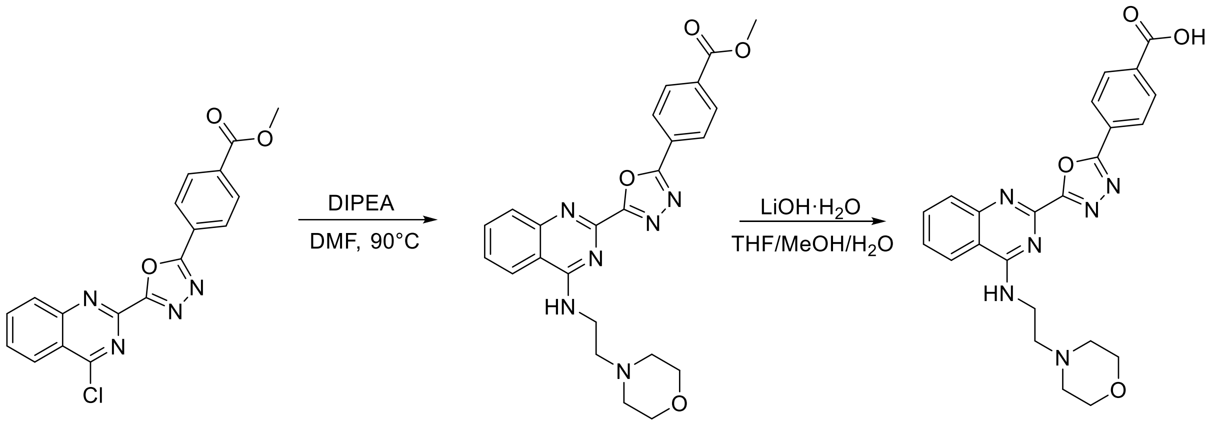

C3 D1 D2

Methyl 4-(5-(4-((2-morpholinoethyl) amino) quinazolin-2-yl)-1,3,4-oxadiazol-2-yl) benzoate **(D1)**

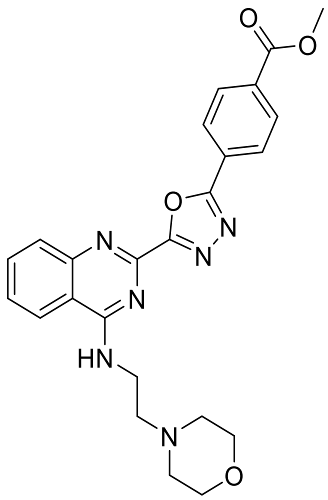

To a solution of **C3** (20.0 mg, 0.05mmol) in THF (5 mL) were added DIPEA (24.1mg, 0.16 mmol) and 2-morpholinoethan-1-amine (7.1 mg, 0.05 mmol). The reaction mixture was stirred at 90 °C for 3 hours. Upon completion of the reaction monitored by LC-MS, the solvent was evaporated and the residue was purified by flash column chromatograph to afford the desired product **D1** (21.6 mg, 46.9 µmol, 86.0% yield).

LC-MS (ESI+) *m/z* calculated for $[C_{24}H_{25}N_6O_4]^+$: 461.2; found, 416.2.

^{1}H NMR (500 MHz, Chloroform-*d*) δ 8.38 – 8.31 (m, 2H), 8.21 (d, *J* = 8.5 Hz, 2H), 8.06 (d, *J* = 8.8 Hz, 1H), 7.84 (t, *J* = 8.2 Hz, 2H), 7.64 – 7.57 (m, 1H), 7.00 (s, 1H), 3.98 (s, 3H), 3.94 (q, *J* = 5.3 Hz, 2H), 3.80 (t, *J* = 4.7 Hz, 4H), 2.81 (t, *J* = 5.9 Hz, 2H), 2.62 (t, *J* = 4.7 Hz, 4H). **^{13}C NMR** (126 MHz, $CDCl_3$) δ 166.20, 164.88, 164.21, 160.00, 149.43, 133.35, 133.04, 130.21, 129.40, 127.67, 127.59, 127.42, 120.95, 114.87, 66.98, 56.48, 53.28, 52.51, 37.17, 33.29.

4-(5-(4-((2-Morpholinoethyl) amino) quinazolin-2-yl)-1,3,4-oxadiazol-2-yl) benzoic acid **(D2)**

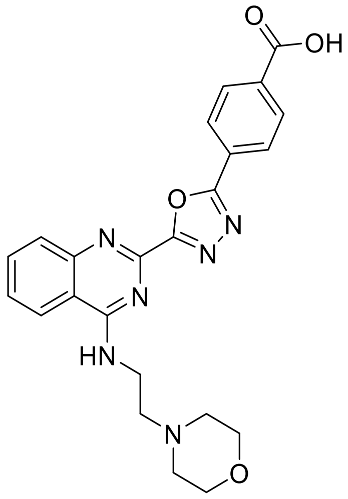

To a solution of **D1** (10.0 mg, 21.7 µmol) in THF/MeOH/H_2O (5 mL, 3:1:1) was added LiOH·H_2O (1.4 mg, 32.6 µmol). The reaction mixture was stirred at room temperature for 3 hours. Upon completion of the reaction monitored by LC-MS, the solvent was evaporated under the vacuum. Water (2 mL) was added to the residue and 1 M HCl was dropped to adjust the pH value to 5, the resulting solid was filtered and dried under the vacuum to afford desired product **C5** as a light-yellow solid (6.9 mg, 15.4 µmol, 71.2% yield), which was used in the next step without further purification.
LC-MS (ESI+) *m/z* calculated for $[C_{23}H_{23}N_6O_4]^+$: 447.2; found, 447.0.

(S)-*N*-(2-(2-(3-((2-(4'-((methyl(2-(methylamino)-2-oxo-1-phenylethyl) amino) methyl)-[1,1'-biphenyl]-3-yl) thiophen-3-yl) amino)-3-oxopropoxy) ethoxy) ethyl)-4-(5-(4-((2-morpholinoethyl) amino) quinazolin-2-yl)-1,3,4-oxadiazol-2-yl) benzamide **(10)**

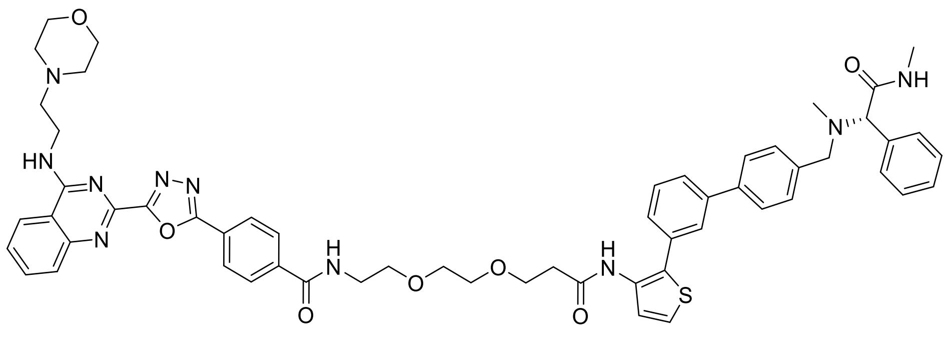

Following the same procedure of **11** in which **L2** was replaced with **L1**, the desired product **10** (12.8 mg, 12.4 µmol, 55.5% yield) was obtained as a white solid.
HR-MS: Calculated for $[C_{57}H_{61}N_{10}O_7S]^+$: 1029.4440; found, 1029.4415.

^{1}H NMR (600 MHz, Methanol-*d*$_4$) δ 8.33 – 8.29 (m, 2H), 8.23 (dt, *J* = 8.1, 0.9 Hz, 1H), 8.03 (td, *J* = 7.5, 6.8, 1.4 Hz, 3H), 7.99 (ddd, *J* = 8.3, 6.9, 1.3 Hz, 1H), 7.81 – 7.73 (m, 4H), 7.70 – 7.59 (m, 5H), 7.59 – 7.51 (m, 5H), 7.41 (d, *J* = 5.4 Hz, 1H), 7.33 (d, *J* = 5.4 Hz, 1H), 4.96 (s, 1H), 4.39-4.29 (br, 1H), 4.19 – 4.14 (m, 2H), 4.14-3.90 (m, 6H), 3.82 (t, *J* = 6.0 Hz, 2H), 3.66 – 3.59 (m, 6H), 3.58 – 3.52 (m, 4H), 2.82 (s, 3H), 2.63 (t, *J* = 6.0 Hz, 2H), 2.59-2.40 (br, 2H). **^{13}C NMR** (151 MHz, MeOD) δ 173.17, 169.01, 168.41, 167.08, 165.31, 162.81, 162.74, 162.51, 150.39, 149.67, 143.96, 141.95, 139.41, 135.69, 135.26, 133.49, 133.09, 132.71, 132.23, 130.96, 130.94, 129.83, 129.60, 129.49, 129.30, 128.94, 128.62, 128.18, 127.81, 127.67, 127.22, 124.83, 123.96, 116.63, 73.00, 71.58, 71.37, 70.56, 68.27, 64.75, 59.61, 53.94, 41.23, 37.92, 37.32, 26.85.

(*S*)-*N*-(2-(2-(2-(3-((2-(4'-((Methyl(2-(methylamino)-2-oxo-1-phenylethyl) amino) methyl)-[1,1'-biphenyl]-3-yl) thiophen-3-yl) amino)-3-oxopropoxy) ethoxy) ethoxy) ethyl)-4-(5-(4-((2-morpholinoethyl) amino) quinazolin-2-yl)-1,3,4-oxadiazol-2-yl) benzamide **(11)**

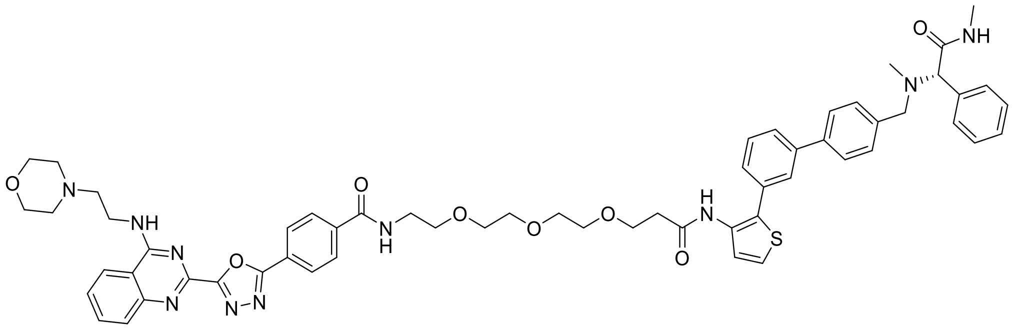

To a solution of **D2** (10.0 mg, 22.4 µmol) in DMF (3 mL) were added DIPEA (14.36 mg, 0.12 mmol) and HATU (12.8 mg, 33.6 µmol). After stirring the reaction mixture at room temperature for 5 min, **L2** (14.4 mg, 22.4 µmol) was added to the reaction mixture, which was stirred for another 1 hour. Upon completion of the rection monitored by LC-MS, the solvent was evaporated under high vacuum and the residue was purified by preparative HPLC which described in **General Purification Procedure for NRAS RIBOTACs** to afford the desired product **10** (9.4 mg, 8.76 µmol, 39.1% yield) as a white solid.

HRMS: Calculated for [$C_{59}H_{65}N_{10}O_8S$] $^{+}$: 1073.4702; found, 1073.4662.

¹H NMR (600 MHz, Methanol-d_4) δ 8.24 – 8.20 (m, 2H), 8.10 (dt, *J* = 8.2, 0.9 Hz, 1H), 7.96 – 7.92 (m, 2H), 7.92 – 7.89 (m, 1H), 7.86 (ddd, *J* = 8.3, 6.9, 1.3 Hz, 1H), 7.70 – 7.65 (m, 3H), 7.63 (ddd, *J* = 8.3, 6.9, 1.3 Hz, 1H), 7.57 – 7.47 (m, 5H), 7.47 – 7.39 (m, 5H), 7.27 (d, *J* = 5.4 Hz, 1H), 7.20 (d, *J* = 5.4 Hz, 1H), 4.85 (s, 1H), 4.26-4.17 (br, 1H), 4.04 (t, *J* = 5.4 Hz, 2H), 4.00-3.80 (m, 6H), 3.65 (t, *J* = 6.0 Hz, 2H), 3.53 – 3.49 (m, 4H), 3.46-3.44 (m, 2H), 3.45 (s, 3H), 3.45 – 3.42 (m, 2H), 3.42 – 3.40 (m, 2H), 2.70 (s, 3H), 2.49 (t, *J* = 6.0 Hz, 2H), 2.46-2.32 (br, 2H). **¹³C NMR** (151 MHz, MeOD) δ 173.17, 169.01, 168.41, 167.07, 165.30, 162.80, 162.73, 162.50, 150.36, 149.66, 143.97, 141.97, 139.49, 135.68, 135.24, 133.49, 132.99, 132.72, 132.23, 130.96, 129.90, 129.82, 129.64, 129.47, 129.27, 128.96, 128.63, 128.17, 127.81, 127.68, 127.25, 124.76, 123.96, 116.62, 72.97, 71.70, 71.47, 71.43, 71.42, 70.55, 68.23, 64.75, 59.60, 53.93, 41.26, 37.98, 37.32, 26.85.

(S)-*N*-(15-((2-(4'-((Methyl(2-(methylamino)-2-oxo-1-phenylethyl) amino) methyl)-[1,1'-biphenyl]-3-yl) thiophen-3-yl) amino)-15-oxo-3,6,9,12-tetraoxapentadecyl)-4-(5-(4-((2-morpholinoethyl) amino) quinazolin-2-yl)-1,3,4-oxadiazol-2-yl) benzamide **(12)**

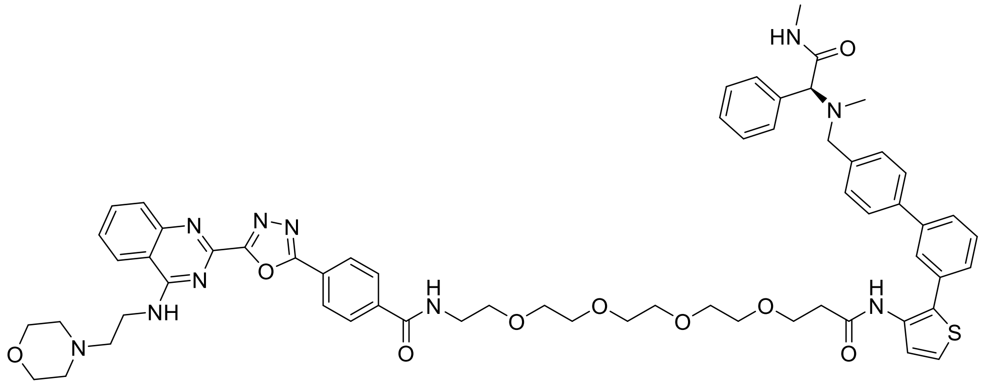

Following the same procedure of **11** in which **L2** was replaced with **L3**, the desired product **12** (10.5 mg, 9.4 µmol, 42.0% yield) was obtained as a white solid.

HR-MS: Calculated for [$C_{61}H_{68}N_{10}O_9S$+Na] ⁺: 1139.4784; found [M+Na] ⁺, 1139.4744.

¹H NMR (600 MHz, Methanol-d_4) δ 8.23 (d, *J* = 8.4 Hz, 2H), 8.10 (d, *J* = 8.2 Hz, 1H), 7.96 (d, *J* = 8.4 Hz, 2H), 7.92 (dd, *J* = 8.3, 1.2 Hz, 1H), 7.86 (ddd, *J* = 8.3, 6.9, 1.2 Hz, 1H), 7.72 – 7.65 (m, 3H), 7.63 (ddd, *J* = 8.2, 6.9, 1.2 Hz, 1H), 7.58 – 7.47 (m, 5H), 7.47 – 7.39 (m, 5H), 7.27 (d, *J* = 5.4 Hz, 1H), 7.21 (d, *J* = 5.4 Hz, 1H), 4.85 (s, 1H), 4.29-4.16 (br, 1H), 4.03 (t, *J* = 5.3 Hz, 2H), 4.00-3.80 (m, 6H), 3.64 (t, *J* = 6.0 Hz, 2H), 3.55 (t, *J* = 5.5 Hz, 2H), 3.53 – 3.46 (m, 9H), 3.44 – 3.38 (m, 6H), 3.36 (dd, *J* = 5.9, 3.4 Hz, 2H), 2.70 (s, 3H), 2.48 (t, *J* = 5.9 Hz, 2H), 2.46-2.32 (br, 2H). **¹³C NMR** (151

MHz, MeOD) δ 173.18, 169.02, 168.38, 167.09, 165.32, 162.80, 162.52, 162.29, 150.36, 149.66, 143.99, 141.98, 139.52, 135.69, 135.23, 133.50, 132.86, 132.76, 132.25, 130.97, 129.83, 129.67, 129.49, 129.31, 128.98, 128.65, 128.22, 127.75, 127.71, 127.26, 124.77, 123.96, 116.63, 73.00, 71.72, 71.63, 71.62, 71.44, 71.43, 71.40, 70.56, 68.23, 64.74, 59.65, 53.93, 41.30, 38.00, 37.33, 26.86.

NMR Spectra

^{1}H NMR Spectrum of 1 (700 MHz, Methanol-d_4):

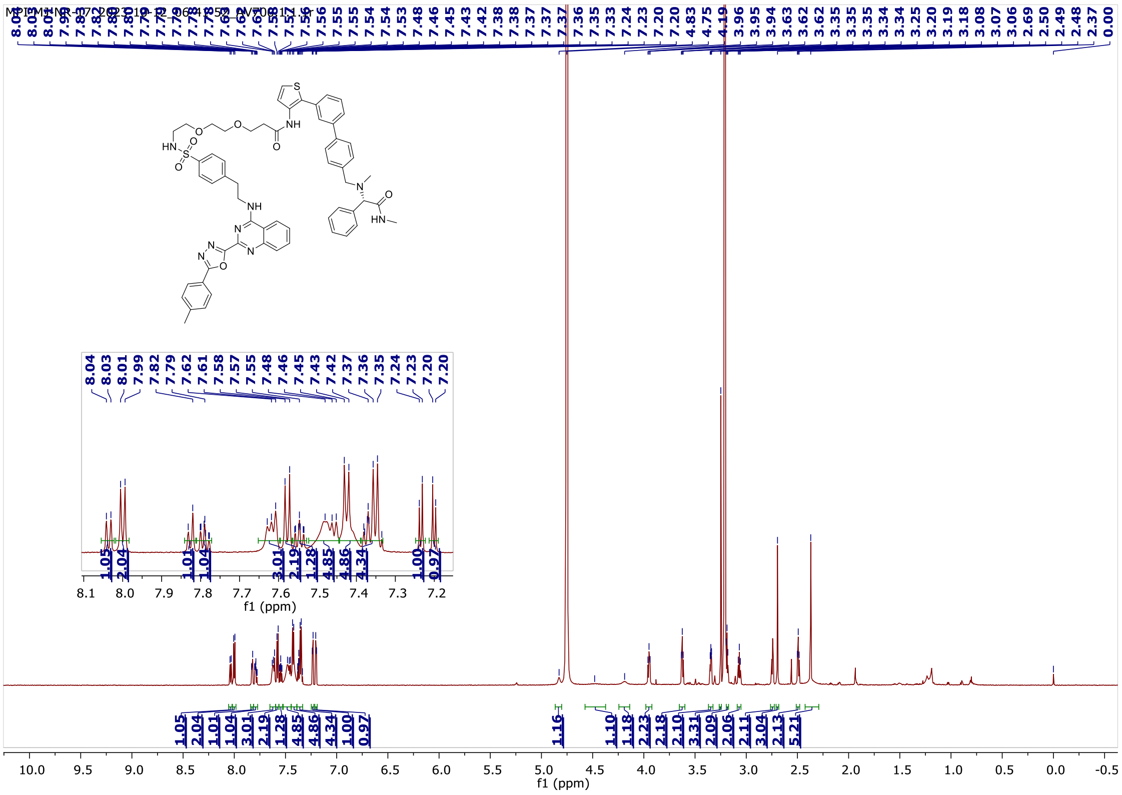

^{1}H NMR Spectrum of 2 (700 MHz, Methanol-d_4):

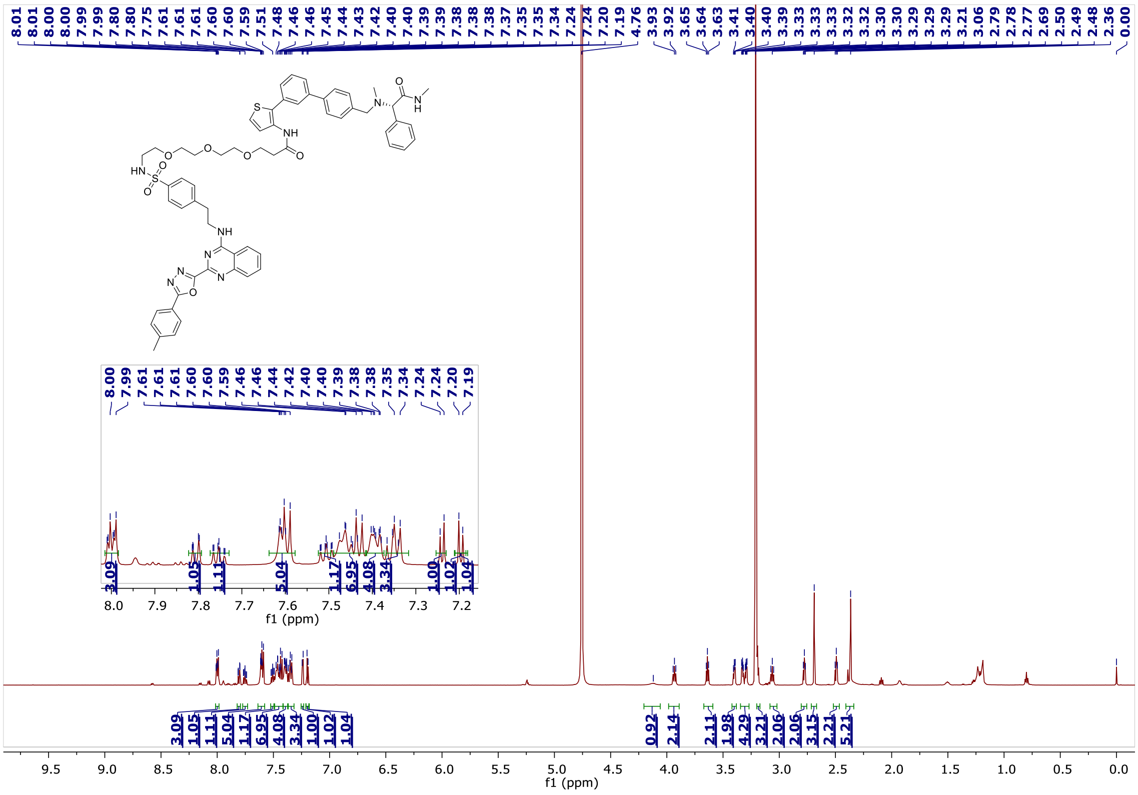

^{1}H NMR Spectrum of **3** (700 MHz, Methanol-d_4):

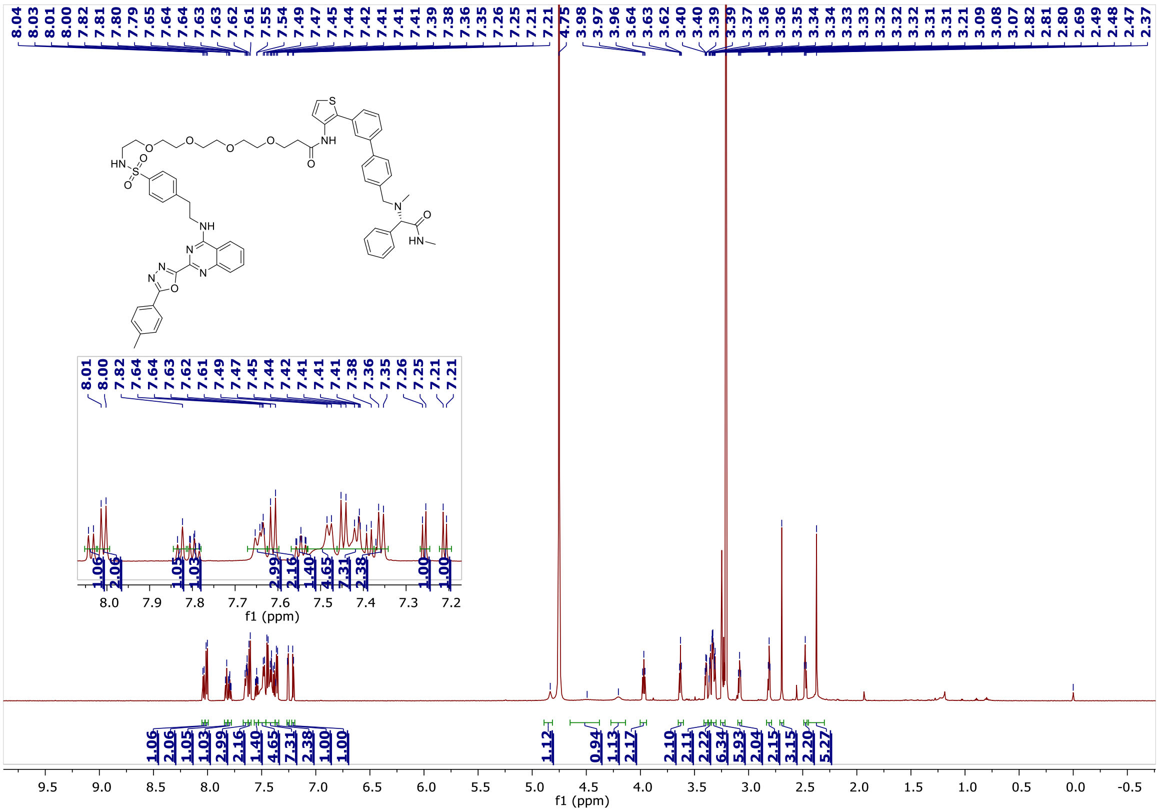

^{1}H NMR Spectrum of **4** (600 MHz, Methanol-d_4):

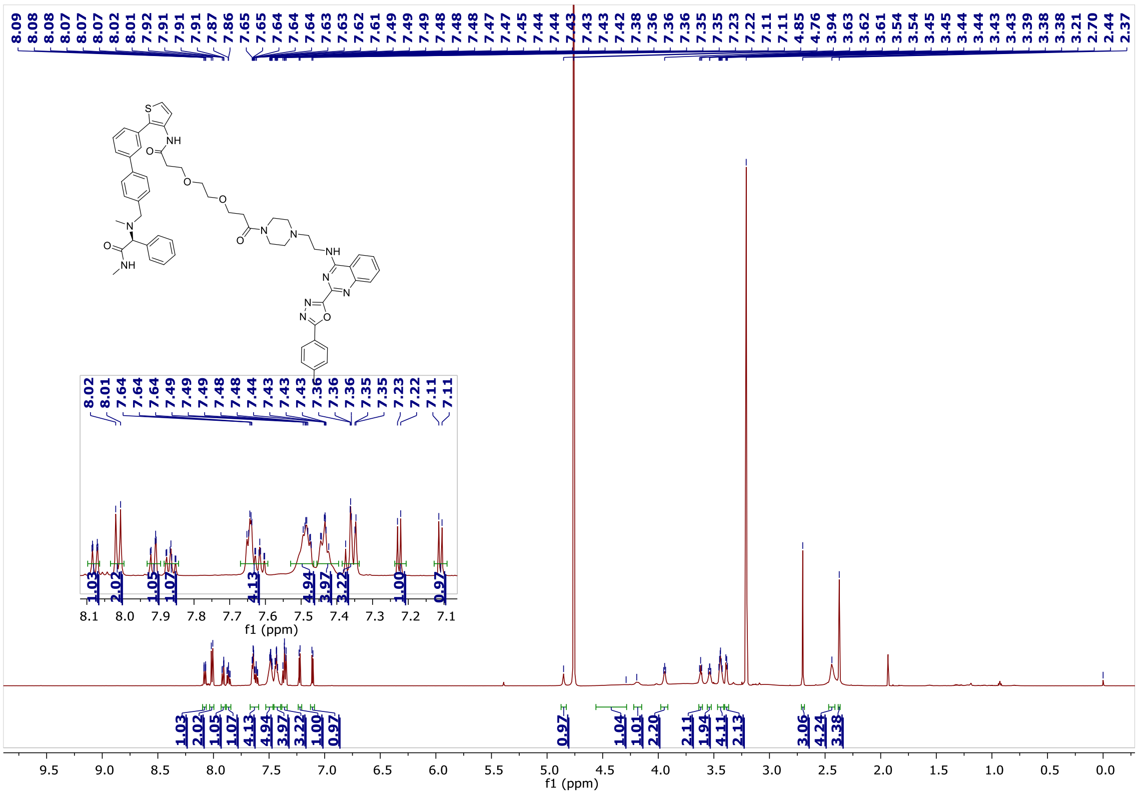

(

^{13}C NMR Spectrum of 4 (151 MHz, Methanol-d_4):

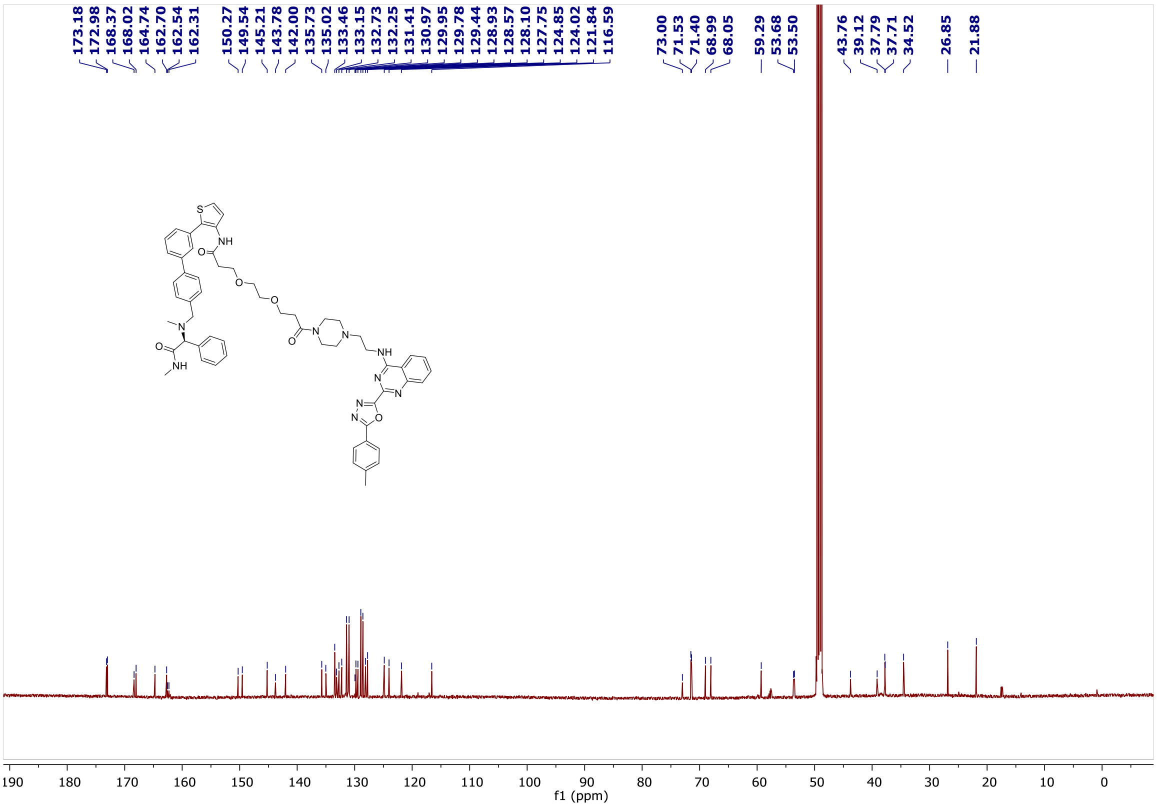

^{1}H NMR Spectrum of 5 (600 MHz, Methanol-d_4):

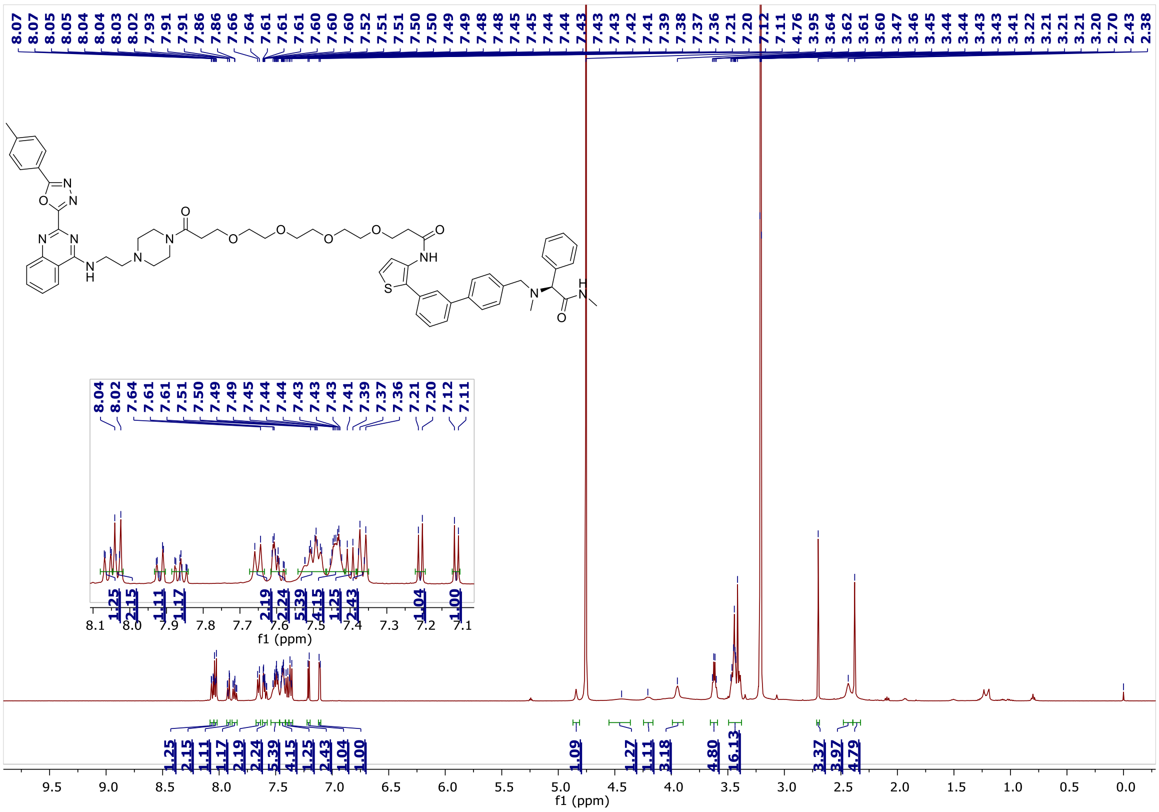

^{13}C NMR Spectrum of **5** (151 MHz, Methanol-*d*$_4$):

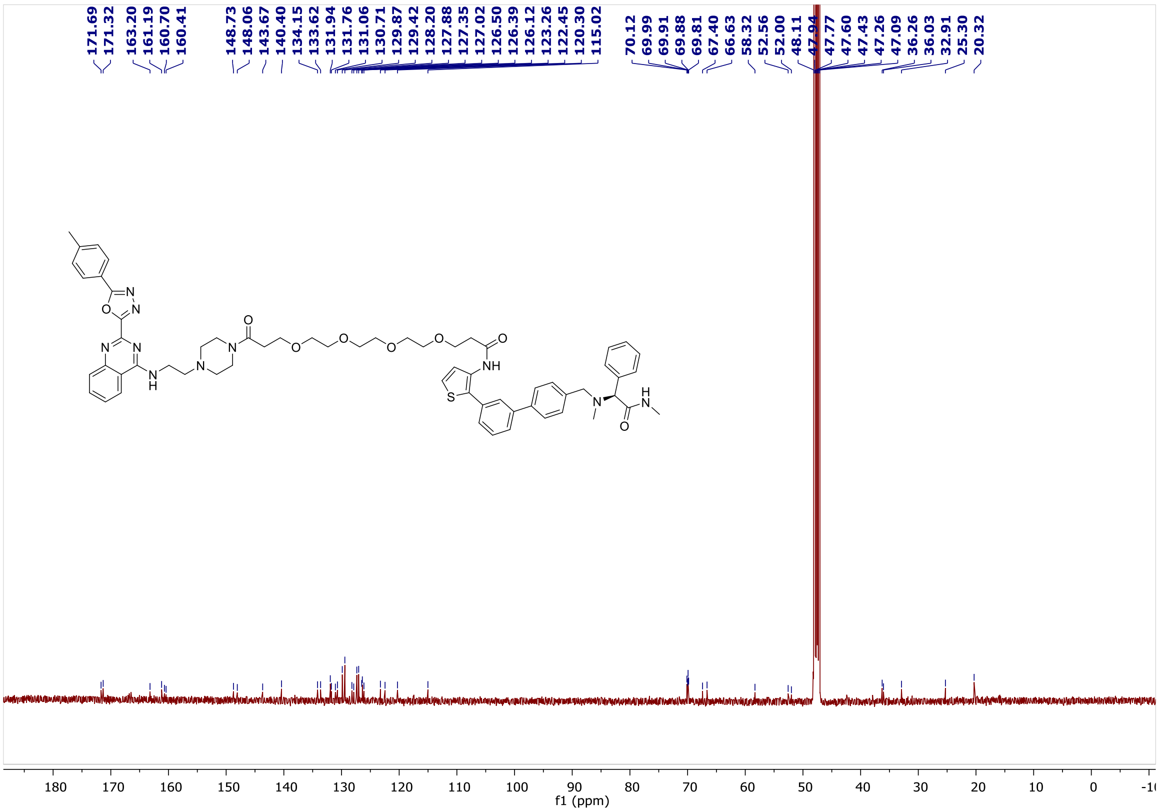

^{1}H NMR Spectrum of **6** (600 MHz, Methanol-*d*$_4$):

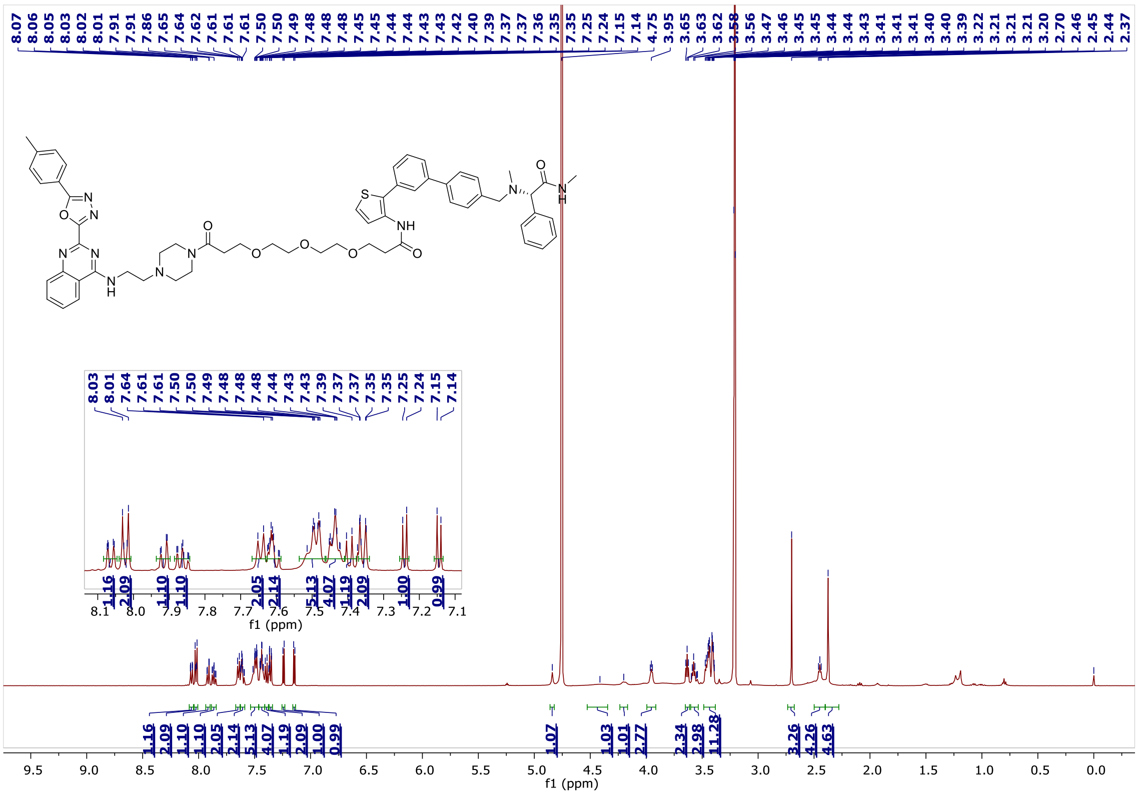

^{13}C NMR Spectrum of **6** (151 MHz, Methanol-d_4):

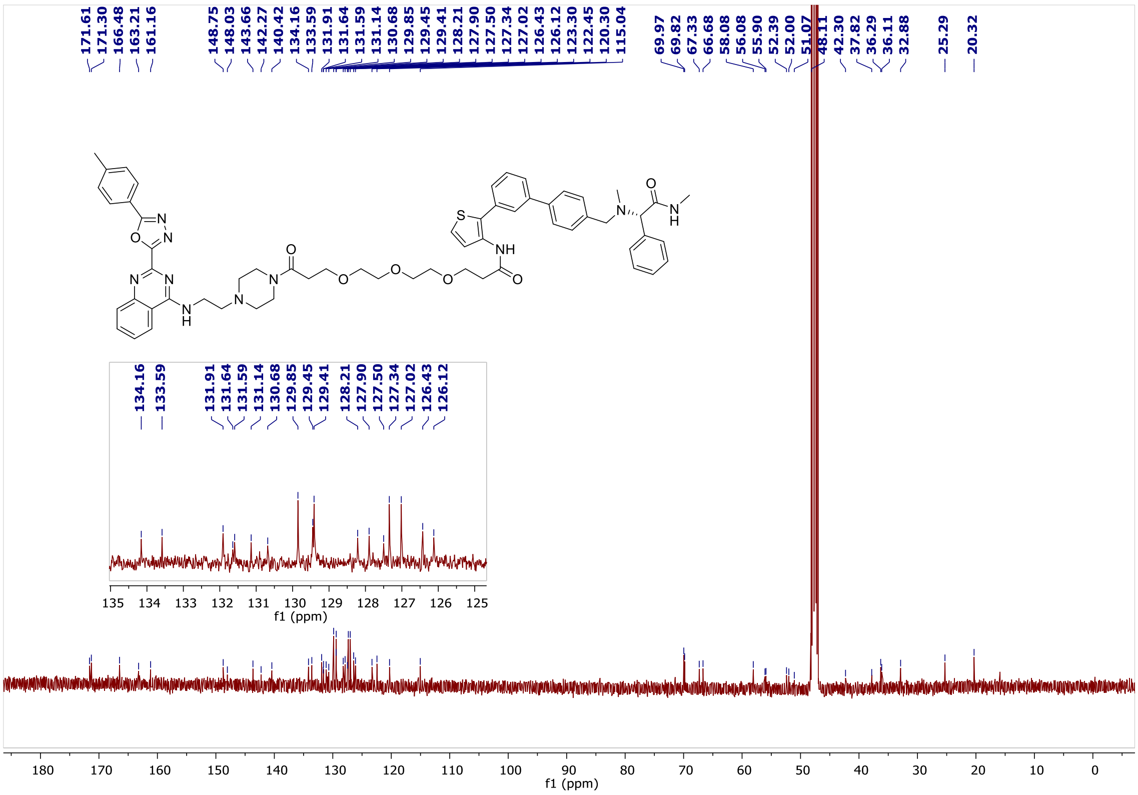

^{1}H NMR Spectrum of **7** (700 MHz, Methanol-d_4):

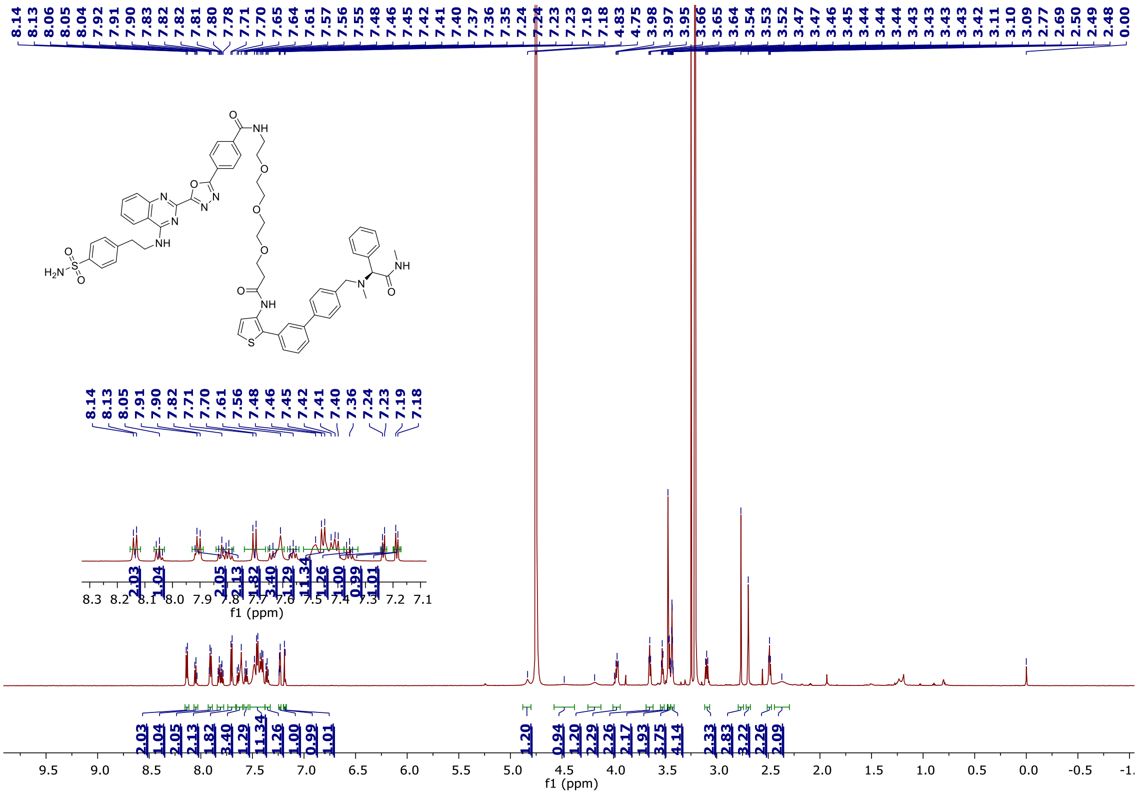

^{1}H NMR Spectrum of **8** (700 MHz, Methanol-d_4):

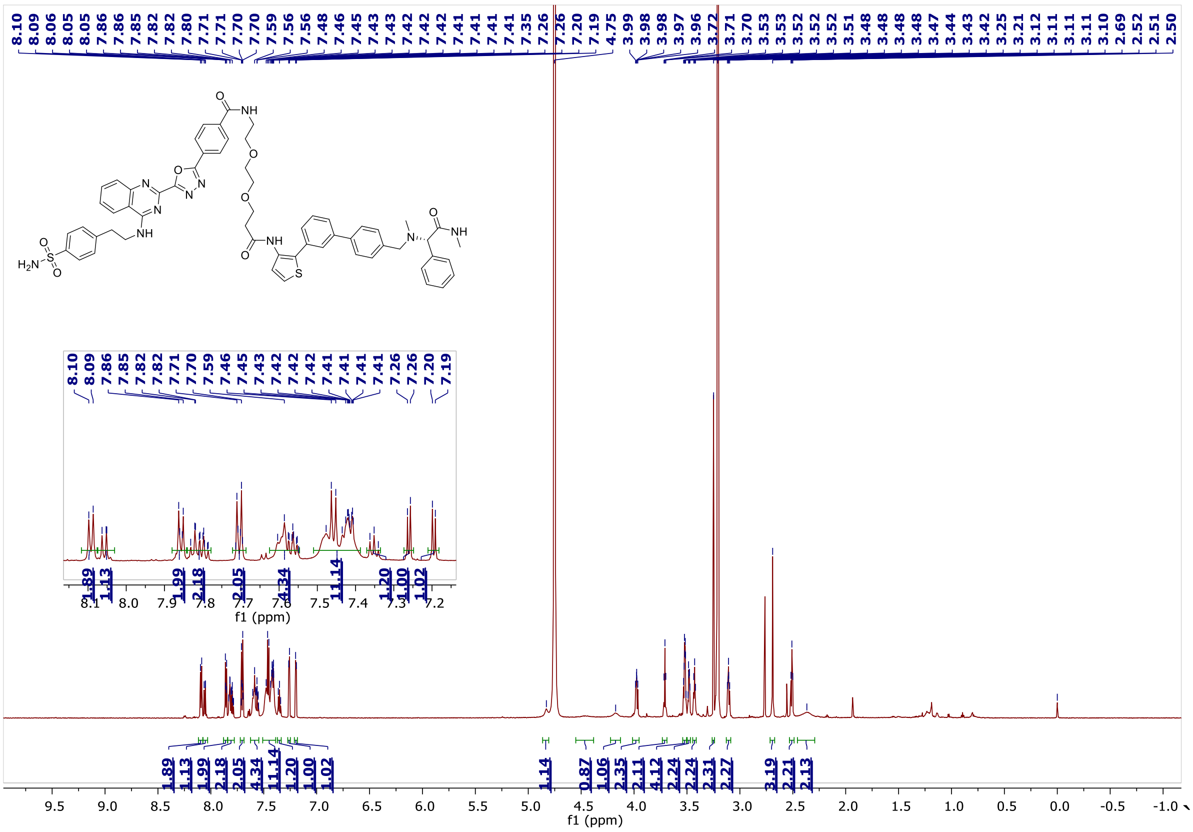

^{1}H NMR Spectrum of **9** (700 MHz, Methanol-d_4):

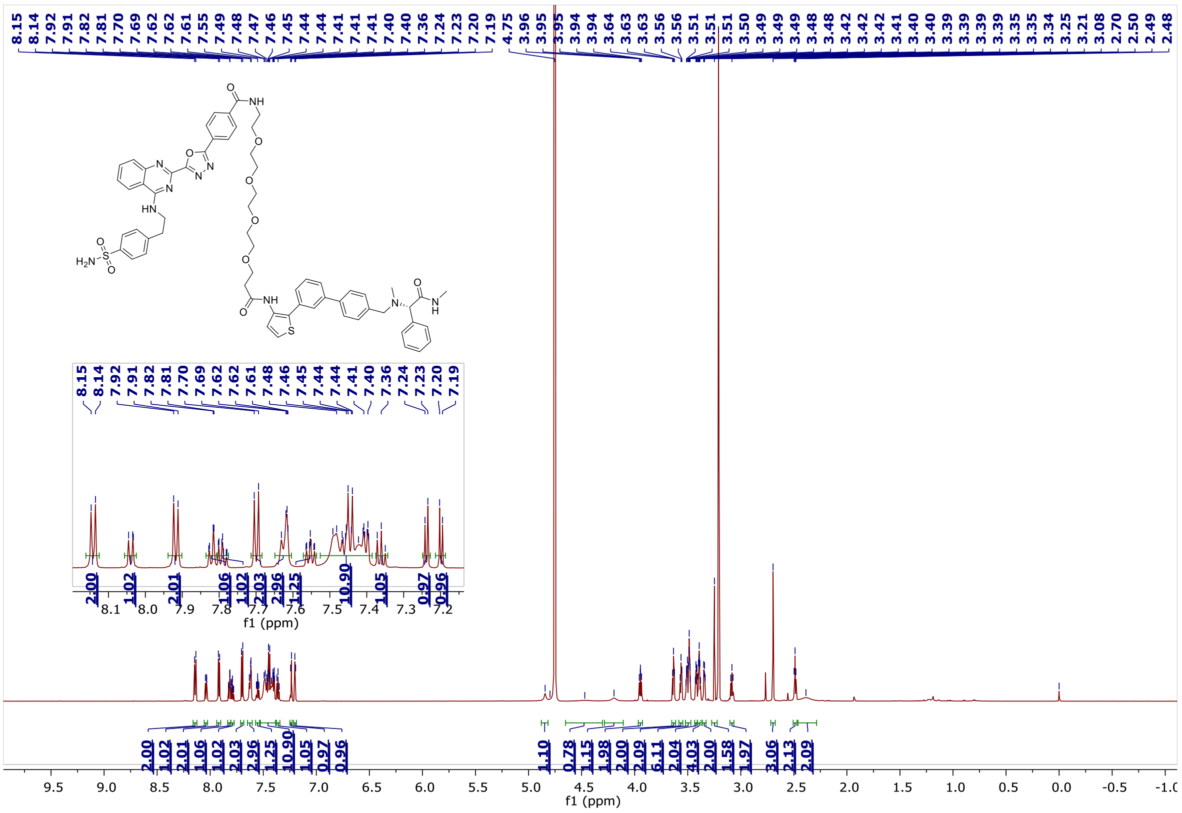

^{13}C NMR Spectrum of **9** (176 MHz, Methanol-*d*4):

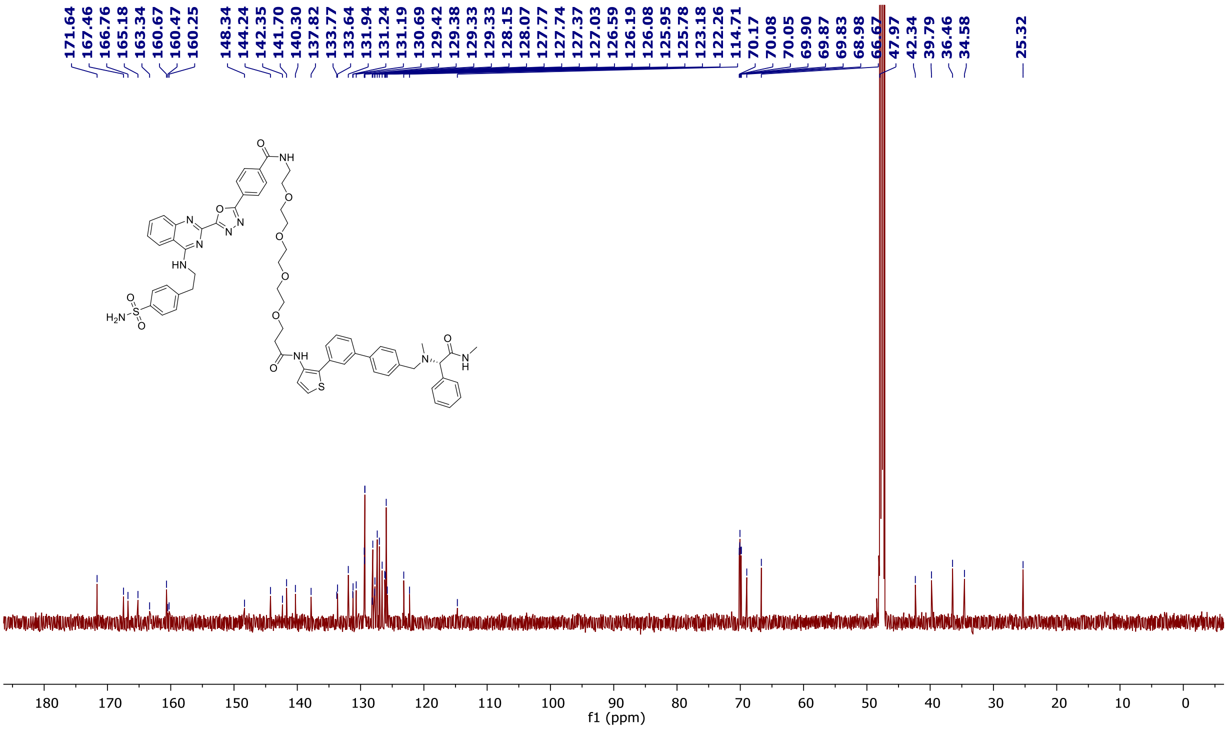

^{1}H NMR Spectrum of **10** (600 MHz, Methanol-*d*4):

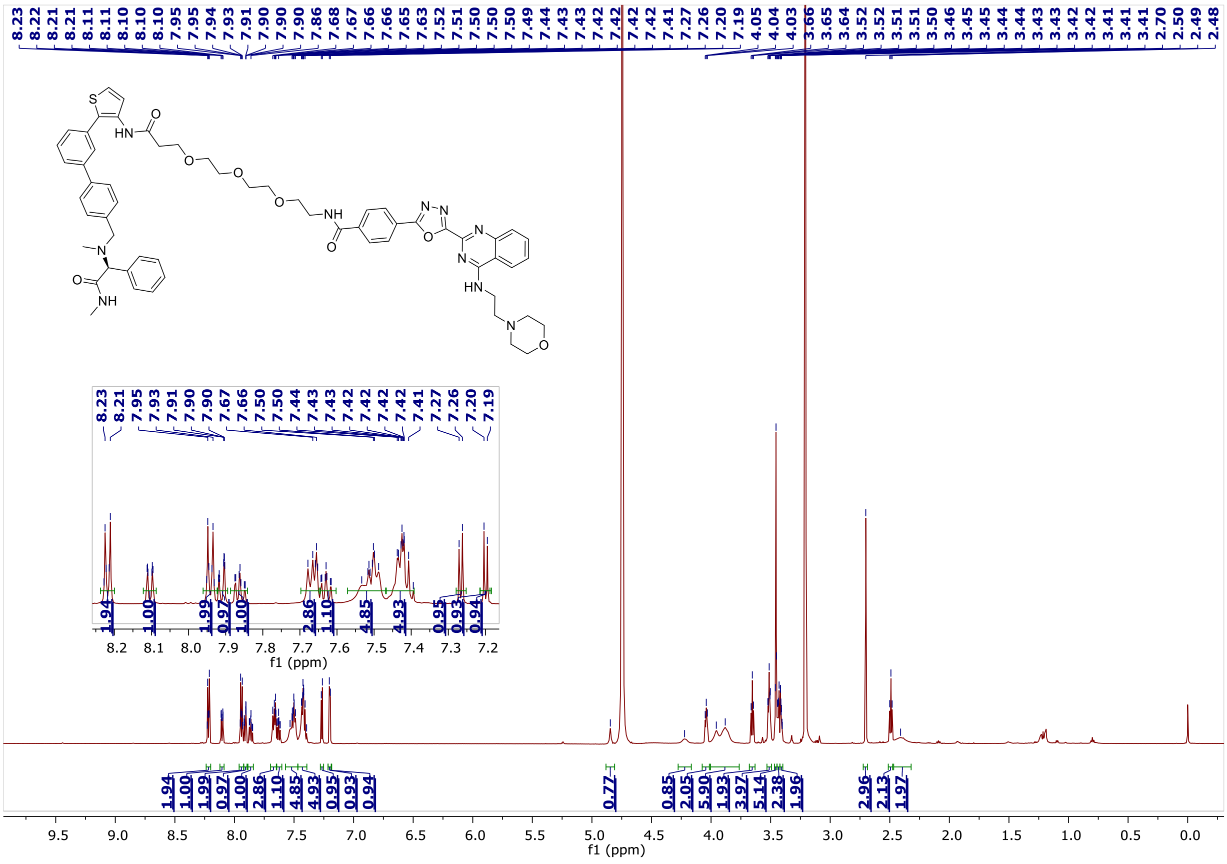

^{13}C NMR Spectrum of **10** (151 MHz, Methanol-*d*$_4$):

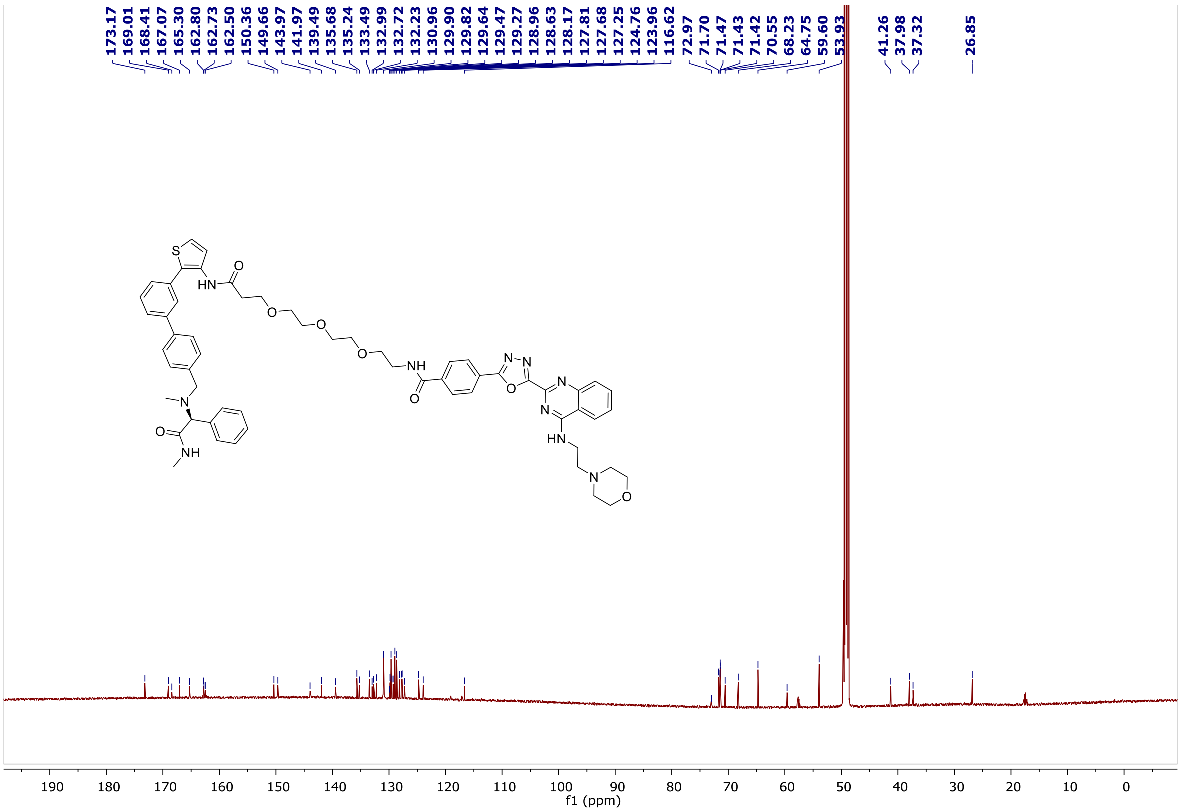

^{1}H NMR Spectrum of **11** (600 MHz, Methanol-*d*$_4$):

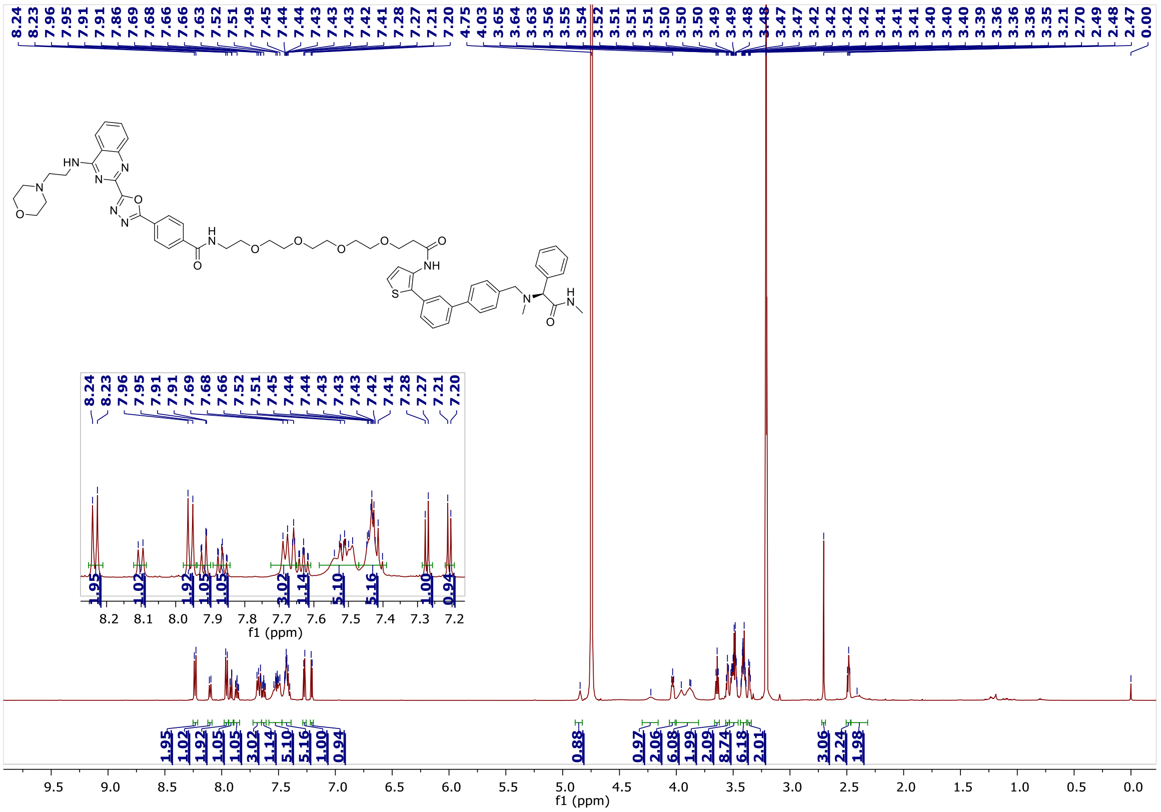

^{13}C NMR Spectrum of 11 (151 MHz, Methanol-d_4):

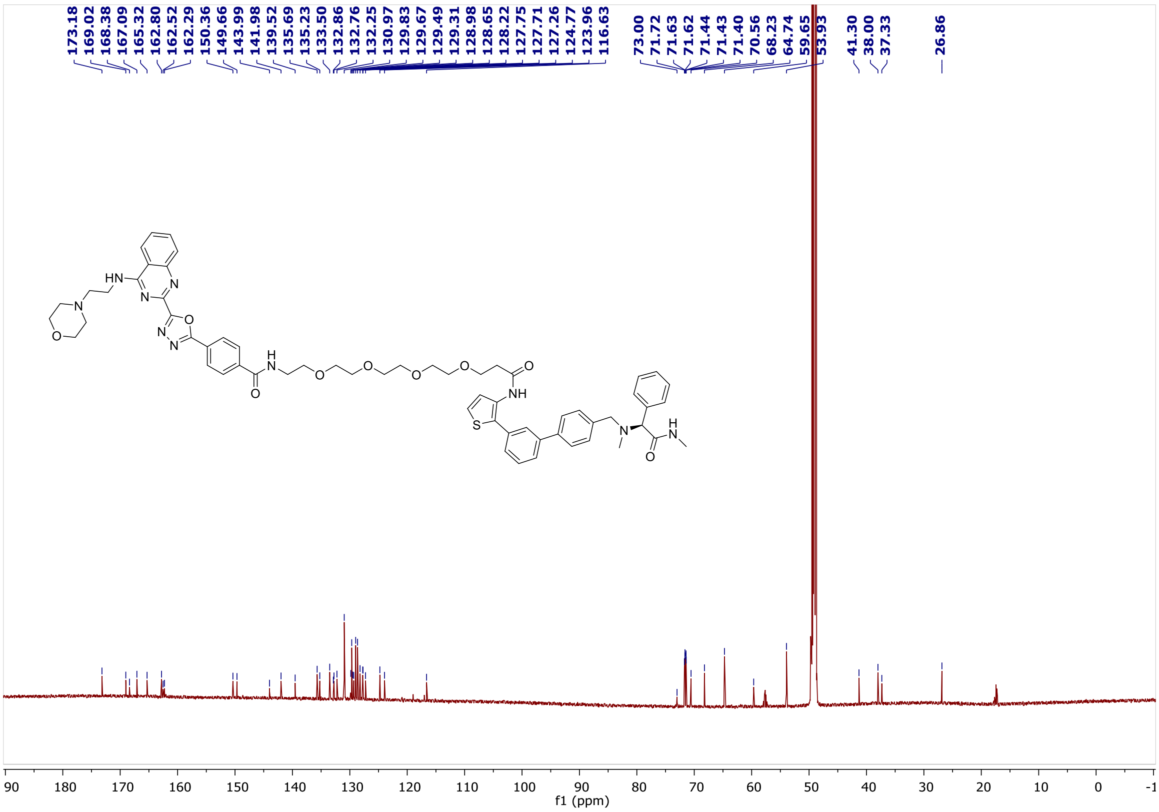

^{1}H NMR Spectrum of 12 (600 MHz, Methanol-d_4):

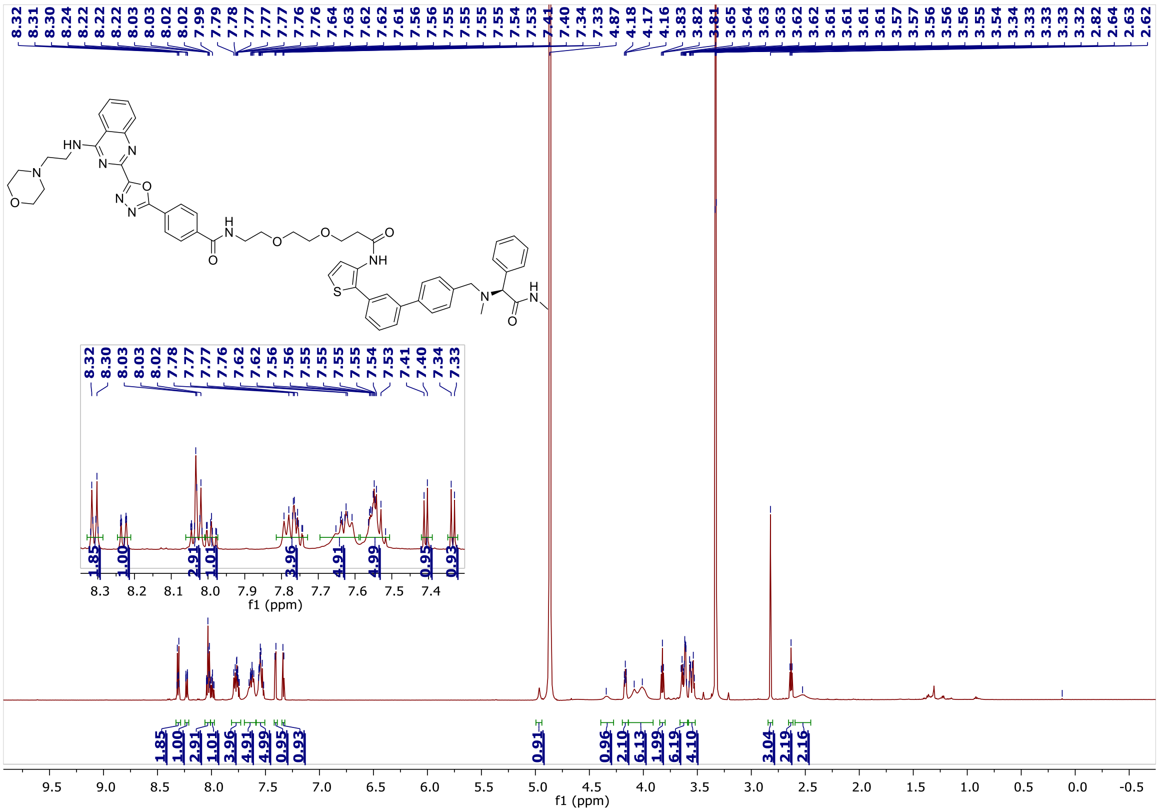

^{13}C NMR Spectrum of **12** (151 MHz, Methanol-d_4):

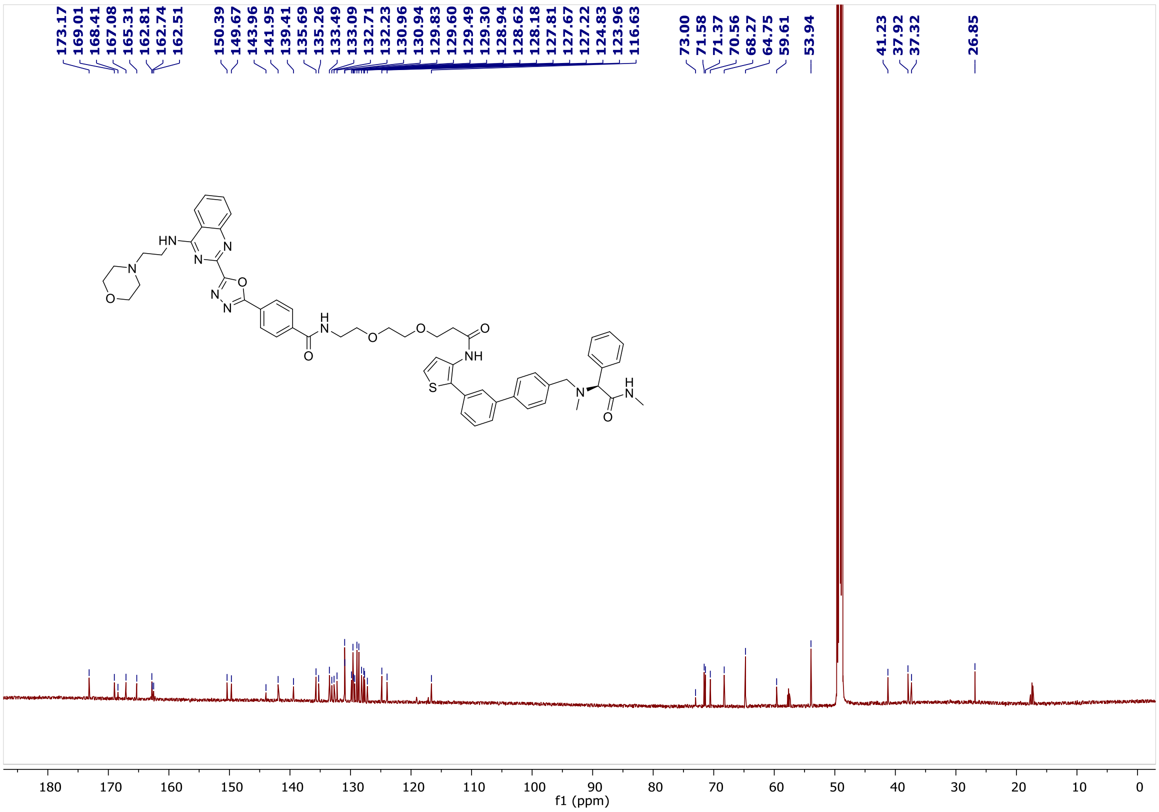

UPLC-MS Spectra

Analytical UHPLC of **1**

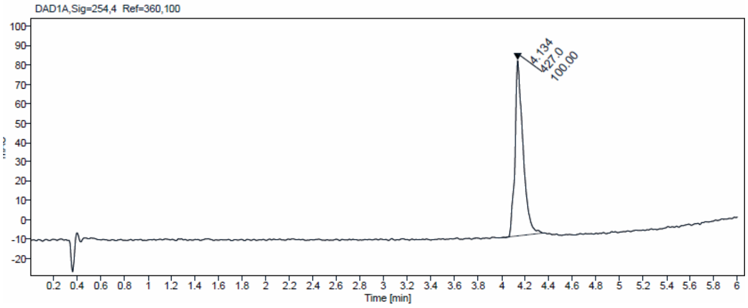

Mass Spectrum of **1**

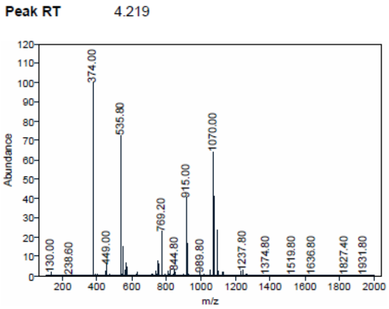

Analytical UHPLC of **2**

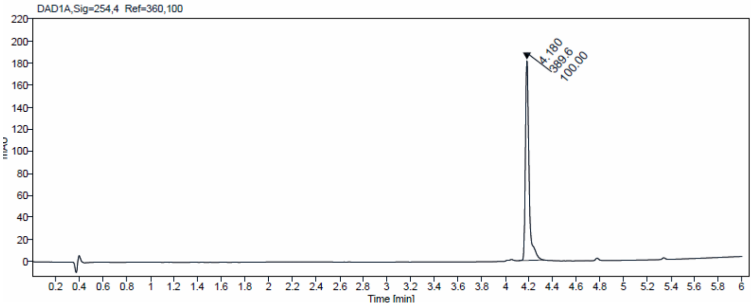

Mass Spectrum of **2**

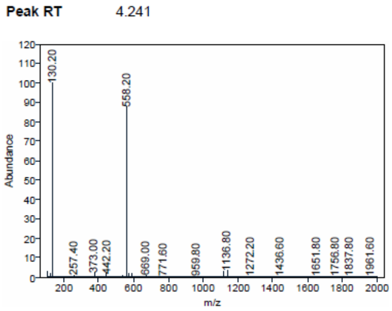

Analytical UHPLC of **3**

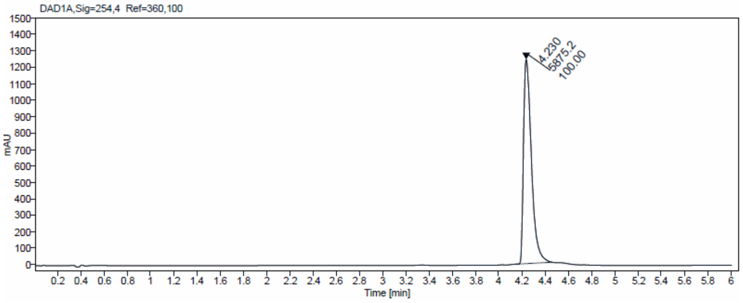

Mass Spectrum of **3**

Peak RT 4.307

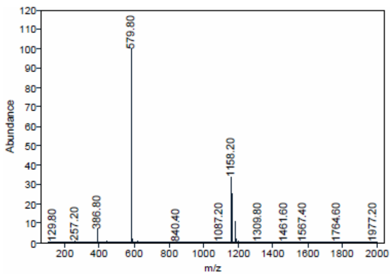

Analytical UHPLC of **4**

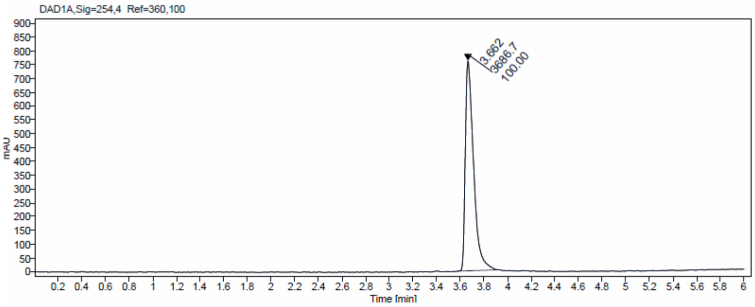

Mass Spectrum of **4**

Peak RT 3.734

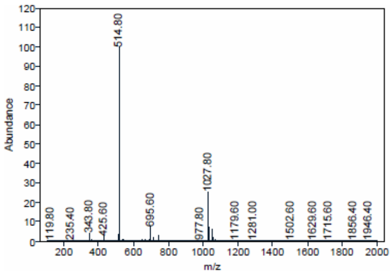

Analytical UHPLC of **5**

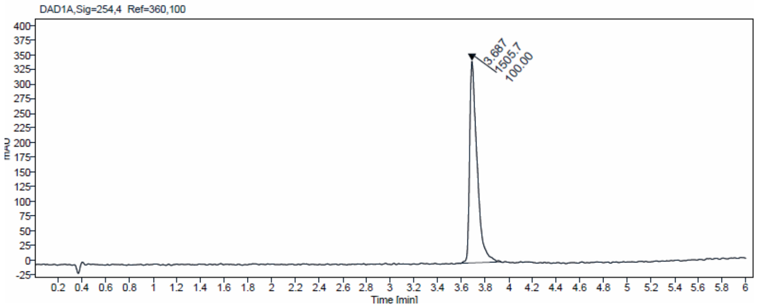

Mass Spectrum of **5**

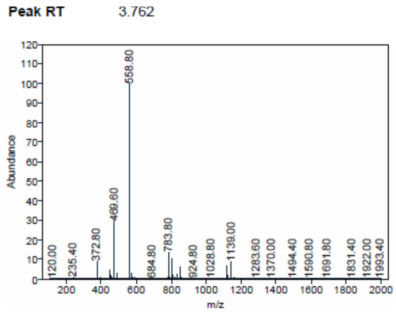

Analytical UHPLC of **6**

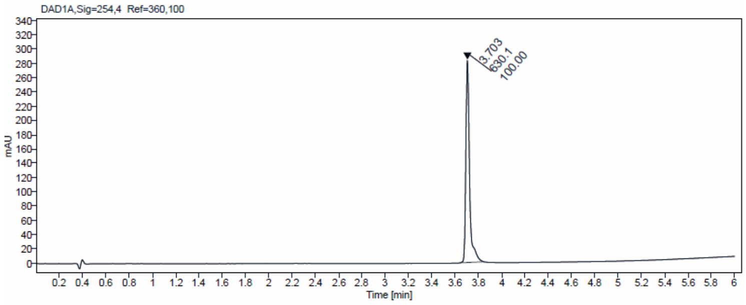

Mass Spectrum of **6**

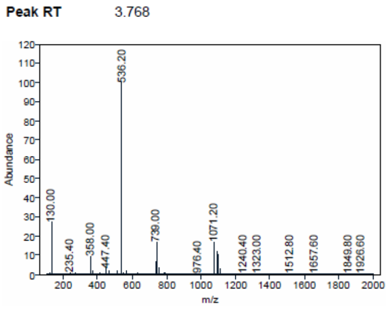

Analytical UHPLC of **7**

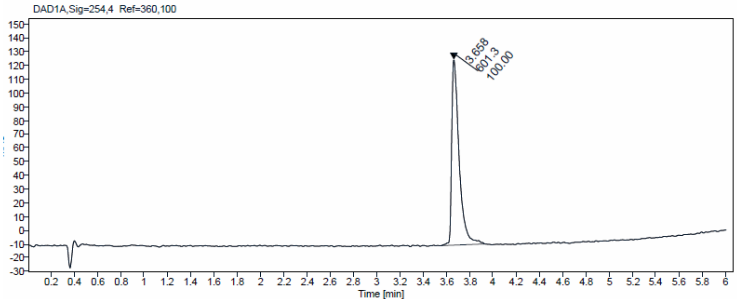

Mass spectrum of **7**

Peak RT 3.772

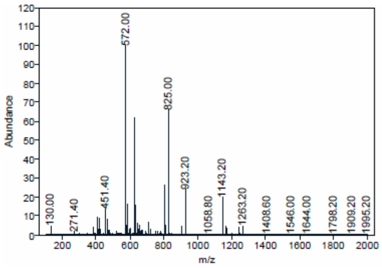

Analytical UHPLC of **8**

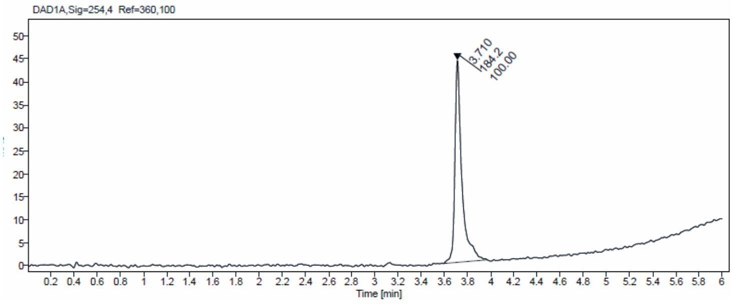

Mass Spectrum of **8**

Peak RT 3.774

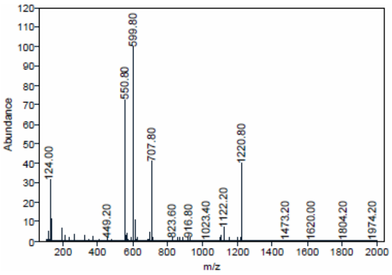

Analytical UHPLC of **9**

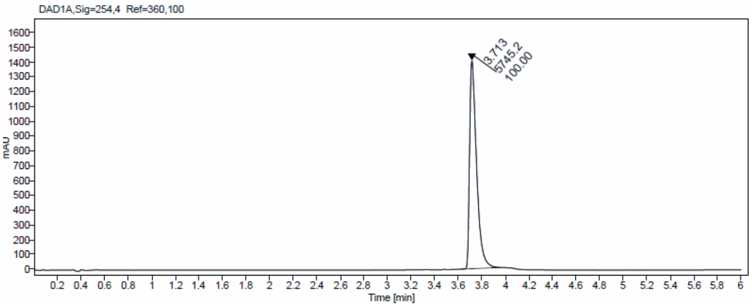

Mass Spectrum of **9**

Peak RT 3.780

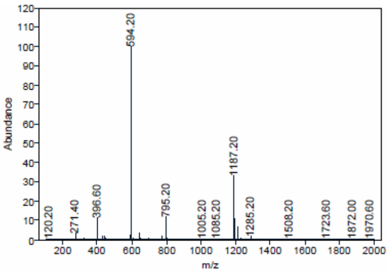

Analytical UHPLC of **10**

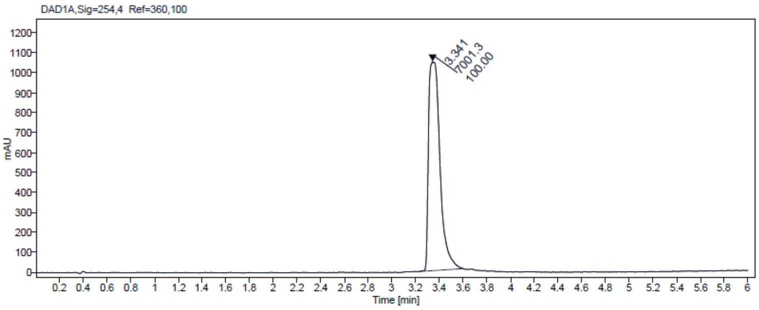

Mass Spectrum of **10**

Peak RT 3.412

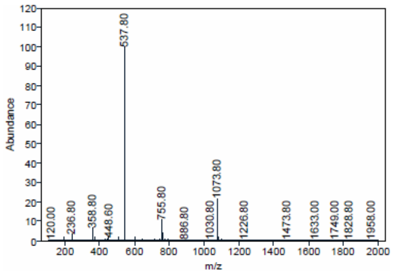

Analytical UHPLC of **11**

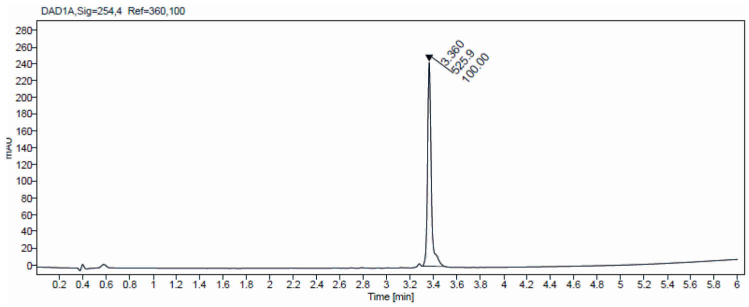

Mass Spectrum of **11**

Peak RT 3.421

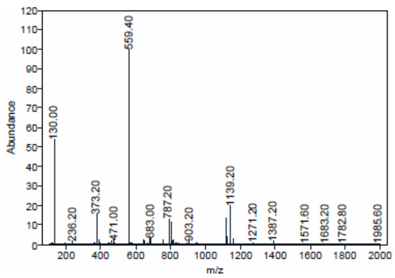

Analytical UHPLC of **12**

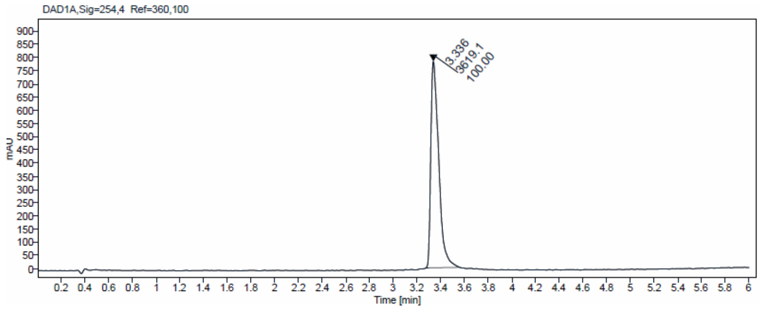

Mass Spectrum of **12**

Peak RT 3.408

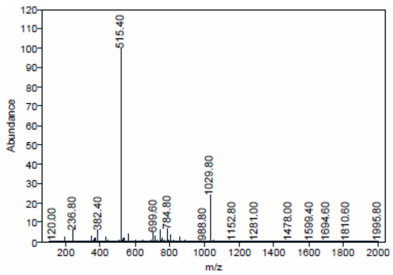

References

(1) Meyer SM, Tanaka T, Zanon PRA, Baisden JT, Abegg D, Yang X, Akahori Y, Alshakarchi Z, Cameron MD, Adibekian A, Disney MD. DNA-Encoded Library Screening To Inform Design of a Ribonuclease Targeting Chimera (RiboTAC). *J. Am. Chem. Soc*. 2022, 144, 46, 21096–21102.

(2) Balaratnam S, Torrey ZR, Calabrese DR, Banco MT, Yazdani K, Liang X, Fullenkamp CR, Seshadri S, Holewinski RJ, Andresson T, Ferré-D'Amaré AR, Incarnato D, Schneekloth JS Jr. Investigating the NRAS 5' UTR as a target for small molecules. *Cell Chem Biol*. 2023, June 15; 30(6): 643-657.e8.
